# Cluster Analysis in Patients with GOLD 1 Chronic Obstructive Pulmonary Disease

**DOI:** 10.1371/journal.pone.0123626

**Published:** 2015-04-23

**Authors:** Philippe Gagnon, Richard Casaburi, Didier Saey, Janos Porszasz, Steeve Provencher, Julie Milot, Jean Bourbeau, Denis E. O’Donnell, François Maltais

**Affiliations:** 1 Centre de Recherche, Institut Universitaire de Cardiologie et de Pneumologie de Québec, Université Laval, Québec, Québec, Canada; 2 Rehabilitation Clinical Trials Center, Los Angeles Biomedical Research Institute at Harbor-UCLA Medical Center, Torrance, California, United States of America; 3 Respiratory Epidemiology and Clinical Research Unit, Montréal Chest Institute, McGill University, Montréal, Québec, Canada; 4 Queen’s University and Kingston General Hospital, Kingston, Ontario, Canada; University of Athens Medical School, GREECE

## Abstract

**Background:**

We hypothesized that heterogeneity exists within the Global Initiative for Chronic Obstructive Lung Disease (GOLD) 1 spirometric category and that different subgroups could be identified within this GOLD category.

**Methods:**

Pre-randomization study participants from two clinical trials were symptomatic/asymptomatic GOLD 1 chronic obstructive pulmonary disease (COPD) patients and healthy controls. A hierarchical cluster analysis used pre-randomization demographics, symptom scores, lung function, peak exercise response and daily physical activity levels to derive population subgroups.

**Results:**

Considerable heterogeneity existed for clinical variables among patients with GOLD 1 COPD. All parameters, except forced expiratory volume in 1 second (FEV_1_)/forced vital capacity (FVC), had considerable overlap between GOLD 1 COPD and controls. Three-clusters were identified: cluster I (18 [15%] COPD patients; 105 [85%] controls); cluster II (45 [80%] COPD patients; 11 [20%] controls); and cluster III (22 [92%] COPD patients; 2 [8%] controls). Apart from reduced diffusion capacity and lower baseline dyspnea index versus controls, cluster I COPD patients had otherwise preserved lung volumes, exercise capacity and physical activity levels. Cluster II COPD patients had a higher smoking history and greater hyperinflation versus cluster I COPD patients. Cluster III COPD patients had reduced physical activity versus controls and clusters I and II COPD patients, and lower FEV_1_/FVC versus clusters I and II COPD patients.

**Conclusions:**

The results emphasize heterogeneity within GOLD 1 COPD, supporting an individualized therapeutic approach to patients.

**Trial registration:**

www.clinicaltrials.gov. NCT01360788 and NCT01072396.

## Introduction

According to the Global Initiative for Chronic Obstructive Lung Disease (GOLD) [1] spirometric classification, mild airflow obstruction is defined by a post-bronchodilator forced expired volume in 1 second (FEV_1_) to forced vital capacity (FVC) ratio at a fixed cut-off of <0.70 and an FEV_1_ ≥80% predicted [2]. Although this grading severity system has proved to be of value in the assessment of chronic obstructive pulmonary disease (COPD), it is a simplistic approach, poorly representing the complexity of COPD [3].

According to the Burden of Obstructive Lung Disease (BOLD) study [4], which used the 2006 GOLD consensus report [5], patients with mild COPD represent nearly 45% of patients with COPD, the remainder being GOLD stage 2 to 4. Paradoxically, there is limited information on patients with mild COPD even though they represent a large portion of patients with COPD. While the latest GOLD statement places emphasis on a more broad assessment of the disease [2], there is still a need to refine the GOLD classification, to avoid misclassification of patients with mild COPD. Accordingly, phenotypes could be one promising approach to the clinical heterogeneity of COPD [6]; potentially helping to identify a better type of approach to use for patients with mild disease.

We took advantage of a cohort of symptomatic and asymptomatic patients with mild COPD to explore possible heterogeneity in GOLD 1 COPD and to evaluate whether different subtypes of patients could be identified within this GOLD category. We used cluster analysis to divide our population into subgroups (clusters) according to the clinical parameters included in the study. The participants were characterized in five different domains: 1) baseline characteristics; 2) symptoms; 3) baseline lung function; 4) peak exercise response; and 5) levels of physical activity (steps/day, daily energy expenditure >3 metabolic equivalents [METs], daily time >3 METs). Since the clinical significance of this relatively new category of patients with mild COPD has been questioned [7–9], we also included healthy control subjects in the cluster analysis to investigate how the GOLD 1 patients would be differentiated. Based on the notion that considerable heterogeneity exists within GOLD 2 to 4 COPD [10], we hypothesized that a similar phenomenon would be seen within the GOLD 1 category and that different clinical phenotypes could be identified.

## Methods

### Study Design and Subjects

Data for this study were obtained, pre-randomization, from a single-center study, aimed at characterizing mild COPD and its exercise response to bronchodilation (ClinicalTrials.gov identifier: NCT01360788) and a multicenter clinical study involving 14 investigation sites, aimed at evaluating exercise response to bronchodilation in mild-to-moderate COPD (NCT01072396). The protocols for the individual trials are available in S1 Protocol (NCT01360788) and S2 Protocol (NCT01072396). The patient characterization phase of NCT01072396 has been published by O’Donnell et al [11].

A total of 85 patients meeting the GOLD 1 COPD spirometric classification criteria (post-bronchodilator FEV_1_ ≥80% predicted and FEV_1_/FVC <0.70) [2] and a smoking history ≥10 pack-years were included in the study; 118 healthy subjects with normal spirometry (FEV_1_ >80% predicted and FEV_1_/FVC ≥0.7) served as controls. All subjects must have been in a stable condition for at least 6 weeks before study enrolment. Patients with COPD treated with short or long-acting β-adrenergic bronchodilators were asked to withdraw from their medication from 8 and 36 hours prior to the visit, respectively; similarly, short or long-acting anticholinergic bronchodilators were discontinued 8 hours and 2 weeks prior to the visit, respectively. This was done in order to avoid any confounding effects on exercise testing or pulmonary function. In all groups, subjects were excluded if they presented with any medical condition, other than COPD, likely to influence exercise testing as well as participation in physical activities of daily life (i.e. cardiovascular, neurological, musculoskeletal, locomotor or other respiratory diseases as well as β-blocker therapy).

### Symptoms

The baseline dyspnea index (BDI) [12] was used to quantify the degree of dyspnea on a scale of 0 to 12, where a lower score denotes worse severity. Cough was considered present when occurring daily for 3 months per year, during at least 2 consecutive years.

### Pulmonary Function Testing

Standard pulmonary function tests, including spirometry, lung volumes (by plethysmography) and diffusion capacity (*D* lco) were obtained according to previously described guidelines [13] and related to predicted normal values [14]. The FEV_1_/FVC ratio was compared with the lower limit of the normal (LLN) range according to the National Health and Nutrition Examination Survey (NHANES) III predicted values [15]. The predicted value for inspiratory capacity (IC) was obtained by subtracting the functional residual capacity (FRC) predicted value from the total lung capacity (TLC) predicted value. Maximum voluntary ventilation (MVV) was estimated by multiplying FEV_1_ by 35 [16].

### Exercise Testing

Peak exercise capacity was determined using a walking exercise test, either an incremental shuttle walk test (ISWT) (NCT01360788) or an incremental treadmill exercise test (NCT01072396).

#### Incremental shuttle walking test

The originally described test [17], was modified to add three additional speed steps in order to reach symptom limitation in all participants [18]. Subjects were allowed to run in order to attain maximal exercise capacity. During the ISWT, subjects breathed through a facemask, connected to a portable gas exchange analyzer (Oxycon Mobile, Viasys Healthcare, Jaeger, Germany), which measured oxygen consumption (V′o_2_), carbon dioxide output (V′co_2_) and minute ventilation (V′e). Dyspnea and leg fatigue Borg scores [19] were obtained at baseline and at end of exercise; with higher scores indicating worse severity. Finally, the locus of symptom limitation was determined by asking whether participants stopped exercise because of dyspnea/leg fatigue/both or for another reason.

#### Incremental treadmill exercise test

The incremental treadmill test was performed in a ramp-fashion adapted from the protocol established by Porszasz et al. [20], with a 10 W•min^–1^ and a 15 W•min^–1^ increase for patients with GOLD 1 COPD and control subjects, respectively. As for the ISWT, subjects were connected to a gas exchange analyzer using a mouthpiece and a nose clip. Finally, the same procedure as for the ISWT was implemented for effort perception and locus of symptom limitation.

### Levels of Physical Activity

Physical activity in daily life was monitored during 7 to 14 consecutive days via a monitor (SenseWear ArmBand, BodyMedia Inc., Pittsburgh, PA, USA), which was worn on the right upper arm for at least 12 hours per day. This device produced estimates of the steps taken per day, as well as daily time and energy expenditure associated with at least moderate intensity (>3 METs). We report the mean daily values over the period of measure.

### Cluster Analysis

Hierarchical cluster analysis was used to define homogeneous groups of individuals based on given parameters [21]. This analysis was performed using Ward’s minimum-variance method and distances between individuals were measured in the metric of the pooled within-cluster covariance matrix as proposed by Art and colleagues [22]. The analysis results in groups (clusters) of members who share strong associations, while these associations are weak between members of different clusters [23]. Hierarchical clustering methods first assigned each individual to their own cluster. Then the most similar pairs of clusters (in terms of the chosen distance metric) were merged into a new cluster, so that there was one less cluster. The iteration process continued by merging the next two similar clusters, or new clusters, until all individuals could be included in a cluster. The parameters included in the analysis are shown in Table 1. The number of clusters was determined by using three statistics (pseudo F statistic, pseudo t^2^ statistic and cubic clustering criterion), which performed best in the simulation study of Milligan and Cooper [24].

**Table 1:** Clinical parameters included in the analysis.

Domain	Parameters
Baseline characteristics	Age, sex, weight, height, BMI, smoking status (never/former/active), smoking history (pack-years)
Symptoms	Cough with sputum production for 3 months/year during ≥2 consecutive years (yes/no)
	BDI score (scale 0–12)
Lung function	% predicted value: FEV_1_, FVC, IC, TLC, FRC, RV, *D* lco
	Ratio: FEV_1_/FVC, IC/TLC, RV/TLC
	Lower limit of normal status: FEV_1_/FVC (over/under)
Peak exercise response	V′o_2__peak_ (mL•kg^-1^•min^-1^), V′e_peak_ (L•min^-1^), V′e_peak_/MVV, Vt (% predicted VC), B*f*, V′e/V′o_2_, V′e/V′co_2_, dyspnea _peak_ (Borg score; scale 0–10), leg fatigue _peak_ (Borg score; scale 0–10), limiting factor (dyspnea/leg fatigue/both/other)
Levels of physical activity	Steps per day, daily energy expenditure >3 METs (kcal), daily time >3 METs (min)

BMI: body mass index; BDI: Baseline Dyspnea Index; FEV_1_: forced expiratory volume in 1 second; FVC: forced vital capacity; IC: inspiratory capacity; TLC: total lung capacity; FRC: functional residual capacity; RV: residual volume; *D* lco: diffusion capacity; V′o_2_: oxygen uptake; V′e: minute ventilation; MVV: maximal voluntary ventilation by multiplying FEV_1_ by 35; Vt: tidal volume; VC: vital capacity; B*f*: breathing frequency; V′co_2_: carbon dioxide output; METs: metabolic equivalents.

### Ethics Statement

The parent clinical trials, from which data were obtained for this cluster analysis study, were carried out in compliance with the approved protocols, the principles laid down in the Declaration of Helsinki version as of October 1996 and in accordance with the International Conference on Harmonisation Tripartite Guidelines for Good Clinical Practice. Written informed consent was obtained from all participants; the study protocols, informed consent and patient information were reviewed and approved by local Institutional Review Boards/Independent Ethics Committees. NCT01360788: Comité d’Éthique de la Recherche de I’Institut Universitaire de Cardiologie et de Pneumologie de Quebéc (Québec, QC). NCT01072396: Chesapeake Research Review, Inc. (Columbia, MD); The John F. Wolf, M.D. Human Subjects Committee of the Los Angeles Biomedical Research Institute at Harbor UCLA Medical Center (Torrance, CA); Partners Human Research Committee (Boston, MA); Western Institutional Review Board (Olympia, WA); Springfield Committee for Research Involving Human Subjects (Springfield, IL); Trustees of Dartmouth College, Dartmouth–Hitchcock Medical Center, Committee for the Protection of Human Subjects (Hanover, NH); Saint Francis Hospital and Medical Center Institutional Review Board (Hartford, CT); McGill University Health Center Research Ethics Office (Montreal, QC); Comité d’Éthique de la Recherche de I’Institut Universitaire de Cardiologie et de Pneumologie de Quebéc (Québec, QC); Queen’s University, Health Sciences and Affiliated Teaching Hospitals Research Ethics Board (Kingston, ON); and Centre Hospitalier de l’Université de Montreal (CHUM) Research Ethics Committee (Montreal, QC).

### Statistical Analysis

Results obtained in all patients with GOLD 1 COPD and controls were first shown as frequency distributions and compared between the two groups using Pearson’s Chi-squared statistic tests. Second, comparisons were made between the clusters, which were identified through the cluster analysis. Quantitative variables, expressed as mean ± standard deviation (SD), were compared among clusters using an analysis of variance (ANOVA) model. Following a significant finding, Tukey’s post hoc multiple comparisons technique was used to compare each cluster with the other clusters. Qualitative variables, expressed as percentages, were compared among clusters using Pearson’s chi-squared statistic test. All analyses were done at the level of significance of p<0.05.

## Results

### Heterogeneity in GOLD 1 COPD

**Fig 1:**
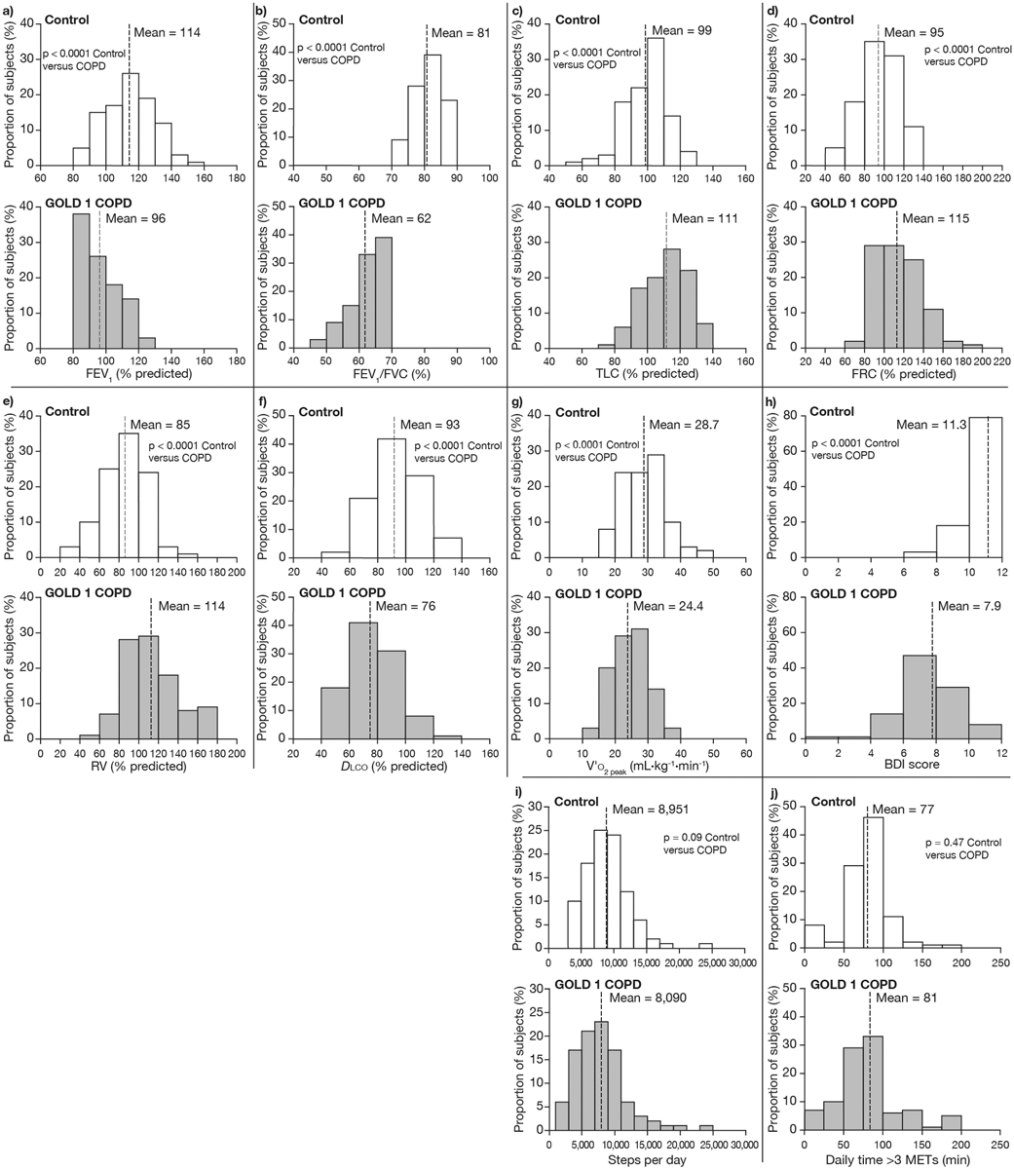
Frequency distributions for pulmonary function, peak V′o_2_, BDI score and physical activity. a) forced expiratory volume in 1 second (FEV_1_); b) FEV_1_/forced vital capacity (FVC) ratio; c) total lung capacity (TLC); d) functional residual capacity (FRC); e) residual volume (RV); f) diffusion capacity (*D* lco); g) peak oxygen uptake (V′o_2_ peak); h) baseline dyspnea index (BDI) score; i) number of steps per day; j) and daily time spent in physical activity >3 metabolic equivalents (METs). GOLD: Global Initiative for Chronic Obstructive Lung Disease.

The frequency distributions for pulmonary function, peak V′o_2_, BDI score and physical activity are provided in Fig 1. FEV_1_% predicted, FEV_1_/FVC and *D* lco % predicted were lower while TLC, FRC and reserve volume (RV) were higher in patients with GOLD 1 COPD compared with controls (all p<0.001). For all these variables, with the exception of FEV_1_/FVC ratio, a considerable degree of overlap between GOLD 1 COPD and controls was seen. Compared with controls, peak V′o_2_ was lower on average by 15% in patients with COPD, who also expressed a lower BDI score. The number of steps per day tended to be reduced in COPD compared with control (p = 0.09). No difference was observed for daily time spent at physical activity >3 METs between the two groups (p = 0.47).

### Cluster Analysis

**Fig 2:**
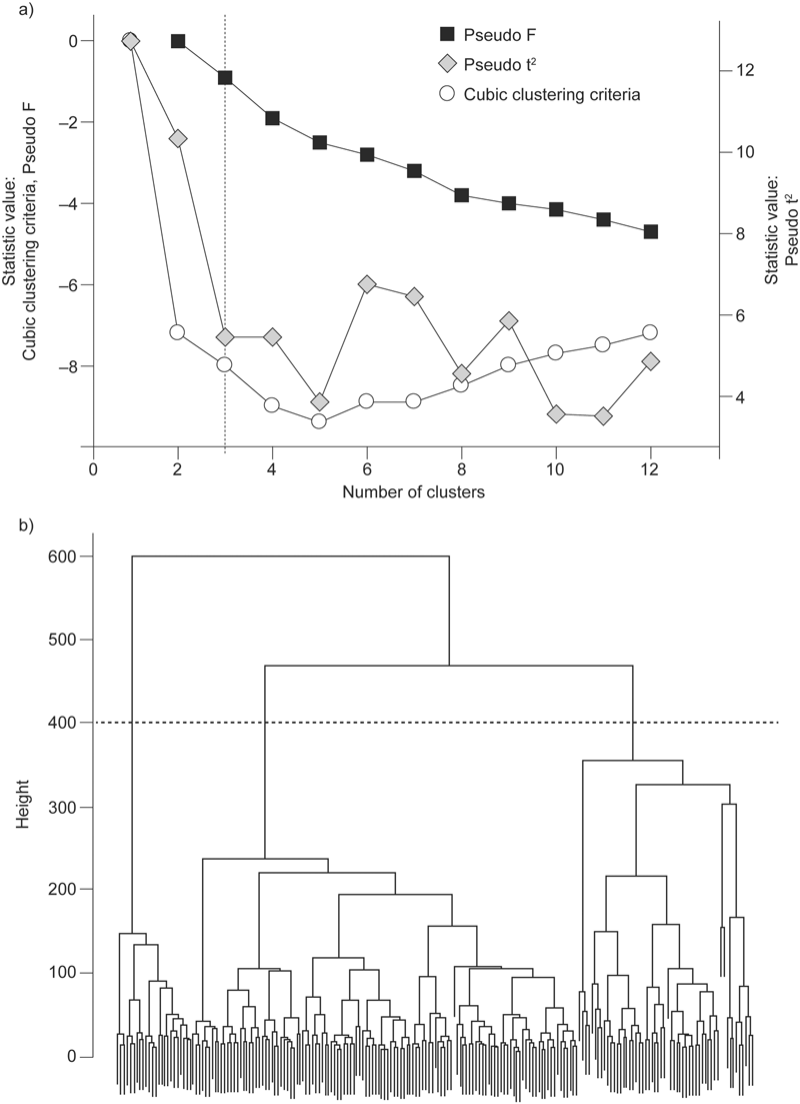
Determination of the number of clusters. a) Using three statistics (the statistical value of pseudo F statistic and cubic clustering criterion are reported on the left Y axis while the statistical value for pseudo t^2^ statistic is reported on the right Y axis) and b) hierarchical Ward’s clustering method.

**Fig 3:**
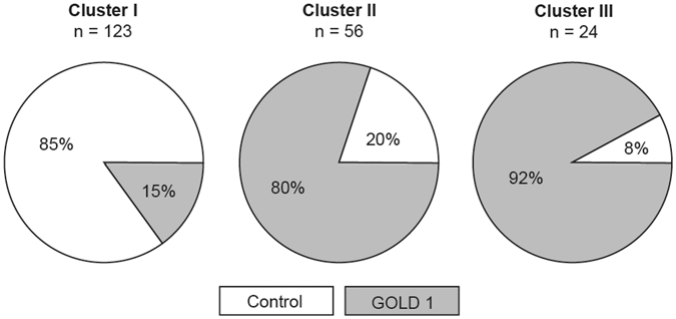
Proportion of controls subjects and patients with GOLD grade 1 COPD within each cluster.

We obtained a three-cluster solution, which best fitted the parameters and subjects included in the study; this decision was based on local peaks of the cubic clustering criterion and pseudo F statistic combined with a small value of the pseudo t^2^ statistic and a larger value for the next cluster fusion (Fig 2a) [24]. This was also in accordance with the dendrogram issued from the hierarchical Ward’s clustering method (Fig 2b) [25]. Cluster I included 105 controls and 18 patients with GOLD 1 COPD, while clusters II and III were mostly composed of patients with GOLD 1 COPD (Fig 3).

**Fig 4:**
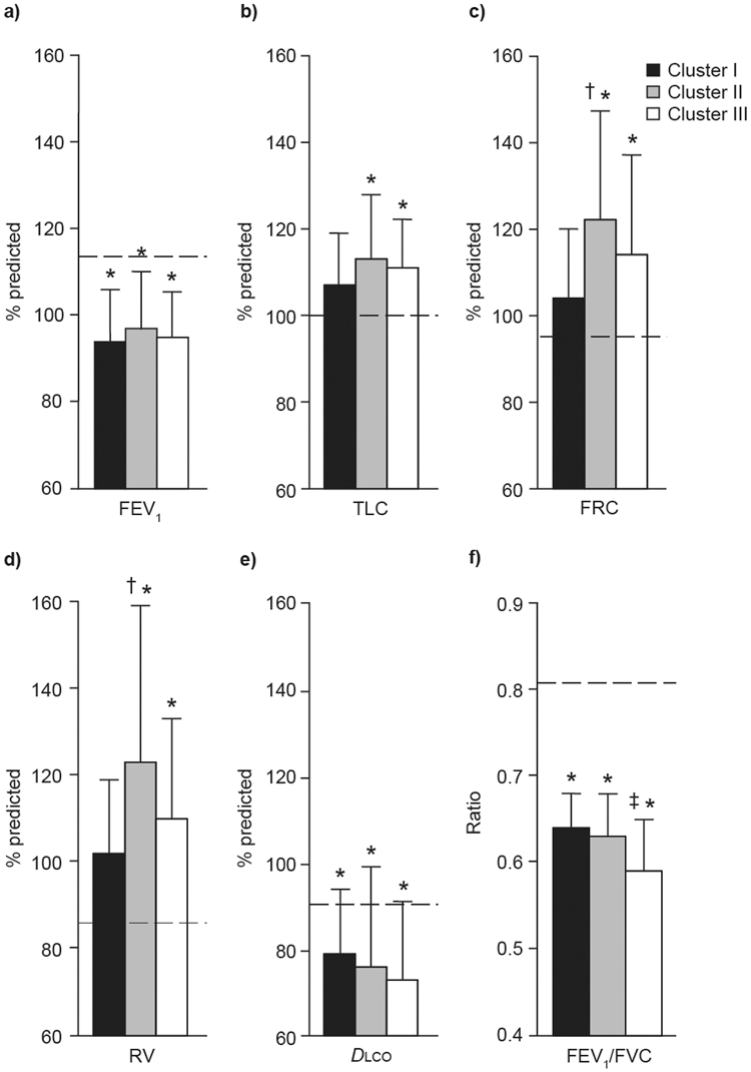
Pulmonary function parameters expressed as percentage of predicted values by cluster. a) forced expiratory volume in 1 second (FEV_1_); b) total lung capacity (TLC); c) functional residual volume (FRC); d) residual volume (RV); and e) diffusion capacity (*D* lco); as well as f) FEV_1_/forced vital capacity (FVC) ratio by cluster of patients with Global Initiative for Chronic Obstructive Lung Disease (GOLD) grade 1 chronic obstructive pulmonary disease. Values are mean ± SD. Dashed lines represent mean values in control subjects. *p<0.01 versus healthy controls; ^†^p<0.05 versus cluster I; ^‡^p<0.01 versus cluster I and II.

The characteristics of the patients with COPD in the three clusters, excluding the controls from this analysis, are presented in Table 2. Patients in the three clusters had a similar body mass index and sex distribution; patients in cluster III were older than those in cluster II (p = 0.03). Smoking history was significantly higher in patients belonging to cluster II than cluster I (p = 0.002). Prevalence of cough and dyspnea BDI scores was similar across the clusters (Table 2). Pulmonary function data are provided in Table 2 and Fig 4. Except for lung volumes in patients belonging to cluster I, other pulmonary function indices were impaired in patients in the three clusters compared with control values (p<0.01; Fig 4). The three clusters of patients with COPD had similar FEV_1_ (Fig 4), but FRC and RV were significantly increased in cluster II compared with cluster I (p = 0.01 and p = 0.04, respectively). Cluster II also tended to display a lower IC/TLC ratio compared with cluster I (0.43 ± 0.08 versus 0.48 ± 0.07; p = 0.07). Finally, cluster III was differentiated from clusters I and II by a significantly lower FEV_1_/FVC ratio (p = 0.002 and p = 0.008, respectively).

**Table 2:** Characteristics of patients with GOLD grade 1 COPD by cluster.

Domain/Parameters	Cluster I (n = 18)	Cluster II (n = 45)	Cluster III (n = 22)	p-value		
				Cluster I versus II	Cluster I versus III	Cluster II versus III
**Demographics**						
Age, years	64 ± 6 [61–66]	61 ± 8 [58–64]	66 ± 6 [63–69]	0.46	0.58	0.03
Male, n (%)	9 (50)	30 (67)	14 (64)	0.46		
BMI, kg•m^–2^	27 ± 3 [25–28]	27 ± 4 [26–28]	27 ± 4 [25–28]	0.68	0.96	0.84
Smoking status, former/active %	94/6	60/40	59/41	0.02		
Smoking history, pack–years	33 ± 15 [25–40]	51 ± 22 [45–58]	43 ± 15 [36–49]	0.002	0.21	0.20
**Symptoms**						
Cough, no/yes %	61/39	42/58	45/55	0.39		
BDI scale	8.5 ± 1.9 [7.6–9.4]	7.5 ± 2.1 [6.9–8.1]	8.3 ± 1.0 [7.8–8.7]	0.14	0.92	0.25
**Lung function**						
FEV_1_, L	2.60 ± 0.67 [2.26–2.93]	2.79 ± 0.69 [2.58–3.00]	2.54 ± 0.62 [2.26–2.81]	0.55	0.96	0.31
FEV_1_, % predicted	94 ± 12 [88–100]	97 ± 13 [94–101]	95 ± 10 [91–100]	0.63	0.95	0.82
FVC, L	4.00 ± 0.98 [3.51–4.49]	4.44 ± 1.14 [4.10–4.78]	4.30 ± 0.99 [3.86–4.75]	0.30	0.63	0.88
FVC, % predicted	118 ± 13 [111–124]	124 ± 14 [119–128]	130 ± 17 [122–137]	0.32	0.02	0.22
FEV_1_/FVC, %	65 ± 5 [63–67]	63 ± 5 [62–65]	59 ± 6 [56–62]	0.54	0.002	0.008
FEV_1_/FVC, <LLN %	61	76	86	0.18		
IC, L	3.09 ± 0.91 [2.64–3.55]	3.03 ± 1.02 [2.72–3.34]	2.99 ± 0.80 [2.64–3.35]	0.96	0.94	0.99
IC, % predicted	110 ± 20 [100–120]	104 ± 21 [98–110]	109 ± 19 [100–118]	0.57	0.99	0.63
TLC, L	6.33 ± 1.16 [5.75–6.90]	6.95 ± 1.62 [6.46–7.44]	6.60 ± 1.22 [6.06–7.14]	0.27	0.82	0.62
TLC, % predicted	107 ± 12 [101–113]	113 ± 15 [109–118]	111 ± 11 [106–116]	0.20	0.52	0.86
FRC, L	3.25 ± 0.45 [3.03–3.48]	3.92 ± 0.95 [3.64–4.22]	3.64 ± 0.83 [3.28–4.02]	0.01	0.32	0.40
FRC, % predicted	104 ± 16 [96–112]	122 ± 25 [114–129]	114 ± 23 [104–125]	0.02	0.37	0.45
RV, L	2.24 ± 0.41 [2.05–2.45]	2.71 ± 0.81 [2.47–2.95]	2.48 ± 0.50 [2.26–2.70]	0.04	0.53	0.38
RV, % predicted	102 ± 17 [93–110]	123 ± 36 [112–134]	110 ± 23 [100–120]	0.03	0.68	0.21
IC/TLC	0.48 ± 0.07 [0.45–0.52]	0.43 ± 0.08 [0.41–0.46]	0.45 ± 0.09 [0.41–0.49]	0.07	0.52	0.58
RV/TLC	0.36 ± 0.05 [0.33–0.39]	0.39 ± 0.08 [0.37–0.42]	0.38 ± 0.07 [0.35–0.42]	0.27	0.64	0.84
*D* lco, % predicted	79 ± 15 [72–87]	76 ± 23 [69–83]	73 ± 18 [65–82]	0.86	0.64	0.85
**Comorbidities**						
Hypertension, n (%)	8 (44)	16 (36)	6 (27)	–	–	–
Dyslipidemia, n (%)	7 (39)	16 (36)	7 (32)	–	–	–
Coronary heart disease, n (%)	1 (6)	5 (11)	0 (0)	–	–	–
Diabetes, n (%)	0 (0)	2 (4)	0 (0)	–	–	–
Neoplasia, n (%)	2 (11)	0 (0)	0 (0)	–	–	–

Data are mean ± SD [95% confidence interval], unless otherwise specified.

Reported p-values for the ordinal variables (distribution) refer to Chi-squared test values from the contingency analysis.

BMI: body mass index; BDI: baseline dyspnea index; FEV_1_: forced expiratory volume in 1 second; FVC: forced vital capacity; LLN: lower limit of normal; IC: inspiratory capacity; TLC: total lung capacity; FRC: functional residual capacity; RV: residual volume; *D* lco: diffusion capacity.

**Fig 5:**
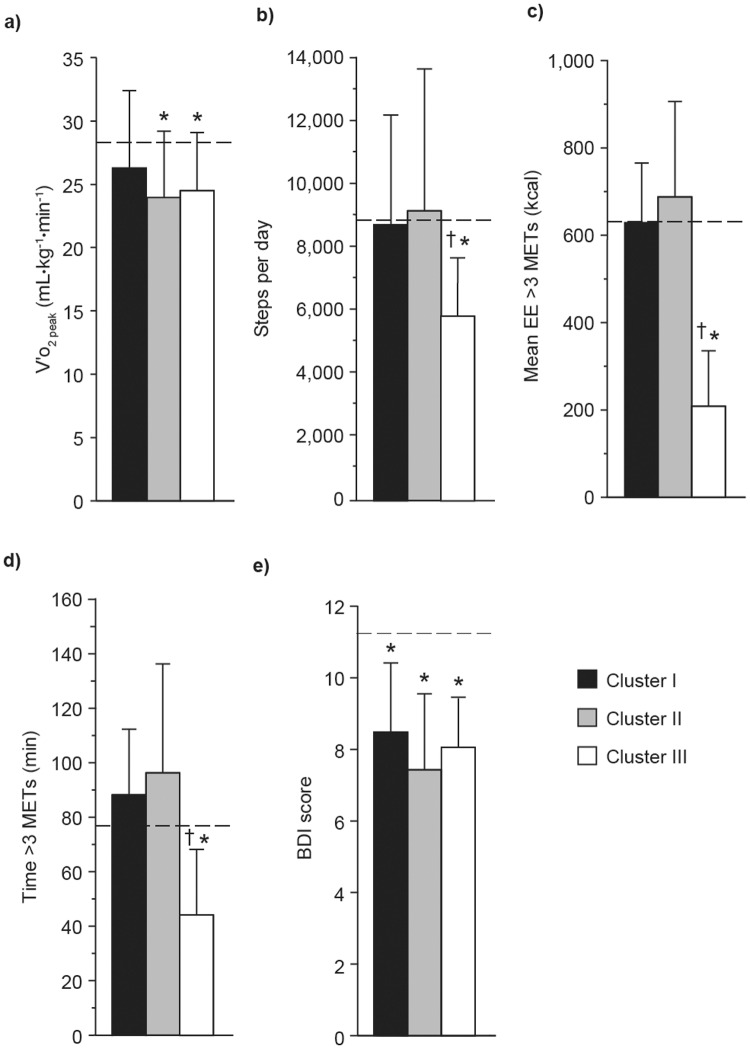
V′o_2 peak_ relative to body weight and daily physical activity levels by cluster. a) Peak oxygen consumption (V′o_2 peak_) relative to body weight; b) mean steps per day; c) mean daily energy expenditure (EE); d) mean daily time >3 metabolic equivalents (METs); and e) baseline dyspnea index (BDI) score by cluster of patients with Global Initiative for Chronic Obstructive Lung Disease (GOLD) grade 1 chronic obstructive pulmonary disease. Values are mean ± SD. Dashed lines represent mean values in control subjects. *p<0.01 versus controls subjects; ^†^p<0.05 versus cluster I and II.

Although patients in the three clusters had similar peak V′o_2_, only patients in clusters II and III showed a significantly reduced peak V′o_2_ compared with controls (p<0.01; Fig 5). The number of step per day and amounts of physical activity with energy expenditure >3 METs was significantly reduced in patients in cluster III compared with controls (p<0.01) and patients in clusters I (p<0.001) and II (p<0.001). As indicated in Table 3, patients belonging to cluster III differed from those of cluster II on the basis of a significantly higher V′e/MVV ratio at peak exercise (p<0.001). When compared with patients in clusters I and II, patients in Cluster III had significantly higher V′e/V′o_2_ ratio (p = 0.005 and p = 0.05, respectively), higher respiratory exchange ratio at peak exercise (p = 0.01 and p<0.001, respectively) and were mainly limited by dyspnea (p = 0.02; Table 3).

**Table 3:** Peak physiological response to exercise for patients with GOLD grade COPD by cluster.

Parameter	Cluster I (n = 18)	Cluster II (n = 45)	Cluster III (n = 22)	p-value		
				Cluster I versus II	Cluster I versus III	Cluster II versus III
Vʹo_2__peak_, mL•kg^-1^•min^-1^	26.3 ± 6.1 [23.2–29.3]	23.7 ± 5.5 [22.1–25.4]	24.5 ± 4.6 [22.4–26.5]	0.22	0.56	0.85
Vʹe, L•min^-1^	73 ± 25 [61–86]	72 ± 25 [64–80]	77 ± 19 [68–85]	0.98	0.89	0.73
Vʹe/MVV	80 ± 17 [72–89]	73 ± 15 [68–78]	87 ± 7 [83–90]	0.15	0.33	<0.001
Vt, % predicted VC	56 ± 11 [51–62]	54 ± 9 [52–57]	61 ± 9 [58–65]	0.71	0.23	0.01
Bƒ, breaths•min^-1^	39 ± 8 [35–43]	37 ± 7 [35–40]	40 ± 7 [38–44]	0.74	0.71	0.19
Vʹe/Vʹo_2_	36 ± 5 [34–38]	38 ± 7 [36–40]	42 ± 5 [40–44]	0.34	0.005	0.05
Vʹe/Vʹco_2_	33 ± 4 [31–35]	35 ± 6 [34–37]	34 ± 5 [32–37]	0.16	0.58	0.72
RER	1.11 ± 0.13 [1.04–1.17]	1.09 ± 0.16 [1.04–1.14]	1.23 ± 0.11 [1.18–1.28]	0.88	0.01	<0.001
Dyspnea _peak_ Borg score	5.7 ± 1.4 [5.1–6.5]	6.4 ± 2.5 [5.6–7.1]	6.8 ± 1.5 [6.1–7.4]	0.57	0.30	0.75
Leg fatigue _peak_ Borg score	4.8 ± 2.4 [3.6–5.9]	5.6 ± 2.7 [4.8–6.5]	5.3 ± 2.3 [4.3–6.3]	0.44	0.79	0.86
Limiting factor dyspnea/legs/both/other, %	28/55/17/0	24/38/38/0	59/23/18/0	0.02		

Data are mean ± SD [95% confidence interval], unless otherwise specified.

Reported p-values for the ordinal variables (distribution) refer to Chi-Squared test values from the contingency analysis.

V′o_2_: oxygen uptake; V′e: minute ventilation; MVV: maximal voluntary ventilation by multiplying FEV_1_ by 35; Vt: tidal volume; VC: vital capacity; B*f*: breathing frequency; V′co_2_: carbon dioxide output; RER: respiratory exchange ratio.

## Discussion

This study highlights heterogeneity in the clinical manifestations of GOLD 1 COPD, as defined by the 2014 GOLD consensus report [1]. Three clusters of patients with GOLD 1 COPD could be identified: cluster I was characterized by reduced *D* lco and decreased BDI dyspnea scores (compared with controls) with preserved lung volumes, exercise capacity and physical activity levels; cluster II showed more prominent static hyperinflation (FRC) and gas trapping (RV) but preserved levels of physical activity; and cluster III exhibited marked reduction in physical activity levels and higher V′e/MVV ratio, V′e/V′o_2_ and respiratory exchange ratio at peak exercise.

Heterogeneity in the clinical manifestations of COPD has been highlighted in patients involved in the ECLIPSE (Evaluation of COPD Longitudinally to Identify Predictive Surrogate Endpoints) cohort [10]. The present study extends these results by showing a similar phenomenon within the GOLD 1 COPD category. One potential implication of these findings is that all patients within the GOLD 1 COPD category should not be considered as having the same disease. Some of them (cluster I) may exhibit preserved functional capacity and physical activity levels despite evidence of airflow obstruction. It could be argued that patients belonging to cluster I were actually healthy subjects who were misclassified based on a fixed FEV_1_/FVC ratio [26]. This issue of misclassification is supported by the fact that the FEV_1_/FVC ratio was >LLN in 39% of subjects. Conversely, decreased BDI dyspnea scores and reduced *D* lco found in this cluster would argue that these patients were nevertheless showing some pathophysiological features of COPD. Clearly, the delineation between healthy smokers and mild COPD is not necessarily perfect, illustrating that the differentiation between health and disease is likely to be a continuum. However, by combining both healthy controls and patients with GOLD 1 COPD in a cluster analysis we have illustrated that the majority of GOLD 1 patients (67/85, 79%) stood out as being different from most healthy controls, which supports the notion that these individuals exhibit clinical features of a “true” disease.

Patients with GOLD 1 COPD included in cluster II were mostly characterized by a substantial smoking history (>50 pack-years) and, from a physiological standpoint, by FRC and RV >120% predicted and reduced exercise capacity. Surprisingly, the levels of physical activity were still preserved in cluster II. It is also interesting to consider that vital capacity was preserved in these individuals despite static hyperinflation and gas trapping. This finding in patients with mild airflow obstruction has been previously reported in cross-sectional studies and may have important implications in terms of maintaining ventilatory capacity during exercise [27–30].

Patients with mild COPD in cluster III were mainly characterized by a lower FEV_1_/FVC ratio, reduced exercise capacity and striking reduction in the level of physical activity compared with the other clusters. Reduced physical activity level has already been reported in patients with GOLD 1 COPD [31,32]. Our data add to the existing literature by showing that this reduction in physical activity may be occurring only in a subset of patients with GOLD 1 COPD. Considering the strong negative prognostic implications of low physical activity levels in COPD [33,34], our analysis may have identified a category of mild COPD that is at higher risk of poor outcomes. Taking into account that increasing physical activity represents an important objective of pulmonary rehabilitation [35], our results support a possible role of this intervention in mild COPD, particularly when FEV_1_/FVC is low. Interestingly, this profound reduction in physical activity level seen in cluster III in comparison with the other two clusters of patients with COPD was present despite similar peak V′o_2_. This dissociation between peak exercise capacity and level of physical activity is important because it illustrates that these two parameters are assessing different concepts and that although preserved peak exercise capacity is permissive to physical activity, it does not guarantee an active lifestyle [36]. We do not have a clear explanation for the reduced level of physical activity in cluster III. These patients had a higher erosion of the ventilatory reserve at peak exercise [37] in comparison with the other clusters and they were mostly limited by dyspnea at peak exercise. In the face of a similar V′e/V′co_2_, the higher V′e/V′o_2_ and respiratory exchange ratio observed at peak exercise in these patients may reflect greater metabolic acidosis, perhaps due to higher reliance of the limb muscles on glycolytic metabolism. Although we can only speculate on this issue, it is interesting to consider that evidence of limb muscle dysfunction has been reported in patients with mild COPD [38,39]. Being more physically inactive, this subset of patients may be at a greater risk of developing limb muscle dysfunction.

In this study, we used the GOLD classification [1] to stratify our patients because of its wide clinical application. However, we appreciate the fact that any attempt to categorize disease severity based on FEV_1_ cut-offs is arbitrary in nature and that, in fact, COPD severity is a continuum. Stratifying patients into subcategories is particularly useful when it helps in disease prognostication or in individualizing clinical management. We acknowledge that we have not reached this goal with the current study. The main purpose of the present cluster analysis was to highlight heterogeneity in GOLD 1 COPD patients; an information potentially useful for future studies in this specific patient population. Our results emphasize that the clinical manifestations of COPD are heterogeneous, even within the same GOLD severity category, and that the evaluation of a patient should not rely solely on FEV_1_. We appreciate that respiratory symptoms were measured only once and that they may fluctuate over time [40]. In order to avoid potential misclassifications for respiratory symptoms, all participants were studied in a stable condition. Given the majority of men in our study, caution should be taken before applying the findings to women with mild COPD. One further potential limitation was that exacerbations were not systematically recorded in this population. Patients involved in study NCT01360788 did not report any exacerbation in the year preceding their involvement in the study, whereas patients involved in study NCT01072396 had to be stable for 6 weeks before the trial. Therefore, we are confident that exacerbation was not a major issue in this population and that this information would not have had a substantial impact on the outcomes of the cluster analysis. It is acknowledged that the sample size for this study is relatively small, since larger sample sizes are not available. However, this is the first time that such a group of patients with GOLD 1 COPD has been thoroughly investigated, and the results should be followed up with a larger sample size when available.

Patient data used in this study were pooled from two clinical trials. Some patients were initially identified through a lung cancer screening study, during which spirometry was performed, and when they had completed their participation in the study, they were referred on for participation in NCT01360788. Patients in NCT01072396 were recruited through respirology clinics in order to evaluate the exercise response to bronchodilation in mild-to-moderate COPD. The resultant patient population for our cluster analysis included a mixture of asymptomatic and symptomatic patients; this is reflected in the heterogeneity that was found in this population. How truly representative this cohort is of the entire GOLD 1 COPD population is difficult to assess but we nevertheless believe that we covered a spectrum of the GOLD 1 COPD population.

## Conclusions

In conclusion, our study highlights heterogeneity within the GOLD grade 1 COPD category. Three clusters of patients with mild COPD were identified: 1) patients with reduced FEV_1_/FVC ratio and *D* lco who otherwise behave like healthy subjects; 2) patients with signs of static hyperinflation but preserved levels of physical activity; and 3) patients with profound reduction in the level of physical activity also exhibiting further reduced ventilatory reserve at peak exercise as well as higher dyspnea score and evidence of more profound metabolic acidosis compared with the other clusters. These results support a more individualized therapeutic approach to patients with GOLD 1 COPD, with some patients potentially only requiring smoking cessation while others being potential candidates for more intense interventions, such as bronchodilation, exercise training and physical activity promotion interventions. This hypothesis and the robustness of our findings will need confirmation in additional population samples.

## Supporting Information

S1 Dataset (XLS)

S1 Protocol NCT01360788 clinical trial protocol. (PDF)

S2 Protocol NCT01072396 clinical trial protocol. (PDF)
