# Supplementary material for: Cluster Analysis in Patients with GOLD 1 Chronic Obstructive Pulmonary Disease
Source: PLoS One. 2015 Apr 23;10(4):e0123626. doi: 10.1371/journal.pone.0123626 (PMC4407903; doi:10.1371/journal.pone.0123626)
Supplement: S1 Protocol — (PDF) [file pone.0123626.s002.pdf]

CENTRE DE RECHERCHE DE L'INSTITUT UNIVERSITAIRE DE CARDIOLOGIE ET DE PNEUMOLOGIE DE QUÉBEC

INSTITUT UNIVERSITAIRE
DE CARDIOLOGIE
ET DE PNEUMOLOGIE
DE QUÉBEC

GOLD stage I COPD: is it really a disease?

Exercise tolerance, muscle function and response to bronchodilation in GOLD 1 COPD

Investigators:

Dr François Maltais, MD
Philippe Gagnon, MSc
Dr Didier Saey, PhD

Exercise tolerance, muscle function and response to bronchodilation in GOLD I COPD

The *Global Initiative for Chronic Obstructive Lung Disease* (GOLD) classification states that a mild (stage I) chronic obstructive pulmonary disease (COPD) is present, in a smoker, when the postbronchodilation forced expired volume in 1 second (FEV_1) to forced vital capacity (FVC) ratio is < 0.7[1;2]. A major change that was introduced by the GOLD classification system was that COPD could be diagnosed despite having an FEV_1 within normal predicted values (above 80% predicted). Because it suggests diagnosing and detecting COPD earlier than done until very recently in medical practice, the GOLD classification brings in a new reality to clinicians. In fact, this novel COPD classification comes with new research challenges because the functional impacts and systemic consequences related to COPD are mostly documented in patients with moderate to severe stages with little information specifically in GOLD stage I COPD. This information is important if we are to convince physicians that GOLD stage I COPD needs to be diagnosed and eventually treated.

Symptomatology

In clinical practice, the diagnosis of COPD relies almost exclusively on bronchial obstruction (objectively determined by spirometry) without referring to symptoms perceived by the patients. However, the underlying symptomatology is likely to be important, especially in mild patients. Indeed, a recent study suggests that the time course of respiratory function in asymptomatic GOLD stage I patients does not differ from healthy individuals[3]. In the same study, the comparison between symptomatic and asymptomatic GOLD stage I patients revealed an accelerated drop in pulmonary function, a reduced quality of life and an increased respiratory care utilization in patients presenting respiratory symptoms[3]. Furthermore, for the same level of bronchial obstruction,

symptomatic mild COPD patients are exposed to a greater risk of mortality[4;5]. Therefore, it's imperative to truly understand clinical consequences linked to mild COPD before stigmatizing individuals with a diagnosis of chronic disease.

Exercise intolerance

In patients with moderate to severe COPD, exercise intolerance represents a hallmark of the disease, the latter being associated to a reduced quality of life and an increased mortality[6;7]. Actually, clinical trials investigating the impact of GOLD stage I COPD on exercise tolerance remains limited. However, some abnormalities to exercise have been described recently in symptomatic GOLD stage I patients : compared with control subjects, COPD patients had increased ventilatory requirements during exercise, associated with greater ventilation/perfusion abnormalities[8]. Nevertheless, we still don't know if these observations can be applied to an asymptomatic GOLD stage I population.

Muscle function

Pulmonary function is weakly related to the intensity of respiratory symptoms perceived by COPD patients[9]. This illustrate that some extra-pulmonary factors may be responsible for the symptomatology of this population. To this end, peripheral muscle dysfunction represents an important consequence of COPD and significantly contributes to reduce exercise tolerance[10;11]. Muscle dysfunction is principally characterized by atrophy, loss of endurance, loss of force, reduced oxidative capacity of the peripheral muscle and an increased fatigability[12-15]. Biopsy studies of the quadriceps in COPD showed that the muscle oxidative profile is reduced in this population[16]. This observation is primarily reflected by a reduced activity in mitochondrial enzymes like citrate synthase (CS) and 3-hydroxyacyl CoA dehydrogenase (HADH)[16]. A reduced proportion of oxidative muscular fibers (type I) in conjunction with a relative increase in glycolytic fibers (type IIB) and a reduced muscular capillarity was also

observed in patients with COPD[17]. However, the presence of these different muscular abnormalities has been highlighted in a moderate to severe COPD population and little is known in mild COPD patients. A study by Doucet and colleagues[18] did not show any particular alteration in peripheral muscular profile in moderate to severe COPD patients compared to a control group. Still, this latter study was interested in highly selected patients (candidates for a thoracic surgery) and the symptomatology of the patients was not considered. Finally, a recent study suggests that peripheral muscular fatigue is a predominant symptom limiting exercise tolerance in GOLD stage I COPD patients[8].

Response to treatments

In GOLD stage I COPD patients, guidelines on disease management basically recommend an intervention on two distinct levels: an active reduction of risk factors and a short-acting bronchodilator treatment when needed[1]. Concerning risks factors, smoking cessation remains the only known intervention to slow the irreversible and progressive decline in pulmonary function seen in COPD[19]. Concerning bronchodilator treatments, little is known on the long-term safety and efficacy of these medications in a mild COPD population[8]. For this reason, the response to bronchodilators becomes an interesting aspect to investigate in patients with mild COPD because making an earlier diagnosis may implicate that this type of medication is prescribed to the patient. Thus, it is pertinent to evaluate the response to bronchodilation in GOLD stage I COPD, not only for its impact on pulmonary function but also on its influence on exercise tolerance. A recent study evaluated the response to *ipratropium bromide* in GOLD stage I COPD [20]. Even though the treatment improved respiratory function, reduced dynamic hyperinflation and dyspnea, the bronchodilation did not show any improvement in endurance time following a symptom-limited constant work-rate cycle exercise. Based on our previous work, we suggest that peripheral muscle fatigue, frequently reported by COPD patients, may have prevented the bronchodilation to improve endurance time[21;22]. In this domain, a

study that we recently completed clearly demonstrated that walking is the exercise modality to prioritize when evaluating functional impact of the bronchodilators in COPD because of it induces less muscle fatigue compared to cycling exercise[21].

Physical activity

Studies using questionnaires or accelerometers have demonstrated a reduced level of physical activities in patients with COPD during daily living[9;23]. This phenomenon constitutes one of the first manifestations of the disease, inexorably conducting to a progressive deconditioning. Thus, COPD patients get trapped in the « dyspnea spiral » of the disease[24], reducing progressively exercise tolerance, which contributes in further reduction in quality of life[25]. A recent study reported a positive correlation between daily walking time and the 6-minute walking test, maximal exercise capacity, lung function, and muscle force[23]. Physical inactivity is associated with increased risks of mortality in various chronic conditions such as COPD[26]. Thus, sedentarity represents a major consequence of COPD, but little is known about its impact on a GOLD stage I disease. It seems that inactivity takes place early in the natural evolution of the disease, probably as early as in GOLD stage I[27;28]. Consequently, the study of COPD in the earlier stage of the disease offers a unique opportunity to clarify and understand the link between physical inactivity and muscle dysfunction in this pathology. Moreover, the relationship between these two features will eventually help in the investigation of muscle disease mechanisms in COPD.

Systemic inflammation and oxidative stress

Systemic inflammation plays a central role in the development and the progression of COPD, as it is associated with premature mortality in this population[29;30]. To this extent, the concentration in high sensibility C-reactive protein (hs-CRP), a blood marker, reflects systemic inflammation in COPD

patients[31]. Concretely, high levels of hs-CRP are associated with reduced physical activity in moderate to severe COPD[27].

COPD is also associated with increased inflammatory mediators such as interleukin-6 (IL-6), a pro-inflammatory cytokine[32]. These mediators could amplify the inflammatory profile in COPD and contribute to certain systemic effects observed in the disease[28].

Finally, oxidative stress represents an important aspect in the physiopathologic comprehension of COPD because of its association with peripheral muscle dysfunction[33;34]. The muscular oxidative stress induced by exercise results in a diminished muscular endurance of the quadriceps in COPD[35].

Consequently, the characterization of limb muscle function, inflammatory status, level of physical activity, exercise tolerance and the response to bronchodilation in GOLD stage I COPD constitutes a promising and interesting research avenue.

Hypotheses

- Exercise tolerance and physical activity level will be reduced in symptomatic GOLD stage I COPD but will be preserved in asymptomatic GOLD stage I COPD;

- Symptomatic GOLD stage I COPD will present abnormalities in peripheral muscle function (force, endurance, typology, capillarity, enzymatic profile);

- Symptomatic GOLD stage I COPD will present systemic inflammation (high levels of hs-CRP, IL-6 and increased oxidative stress);

- ❖ Exercise tolerance in symptomatic GOLD stage I COPD will be improved by bronchodilation;

- ❖ Muscle function and exercise tolerance will not be modified in asymptomatic GOLD stage I COPD when compared with healthy subjects.

Main objective

- ❖ To compare exercise tolerance, muscle function, hs-CRP, IL-6 and oxidative stress blood levels, physical activity level and the response to bronchodilation in symptomatic GOLD stage I COPD compared with asymptomatic GOLD stage I COPD and healthy controls.

Methods

Study design

The study will be conducted among three subgroups: symptomatic GOLD stage I patients with COPD, asymptomatic patients GOLD stage I COPD and healthy control subjects with normal lung function. Subjects will be paired for age, sex and smoking history. The project will require three visits. In the initial visit, assessment of the pulmonary function with respiratory symptoms quantification will allow to classify subjects in the proper. The *Medical Research Council (MRC)* dyspnea scale will serve as the reference outcome to determine whether COPD patients are symptomatic or not[36]. Patients with an MRC dyspnea score < 2 will be considered asymptomatic. A second questionnaire (*ATS-DLD-78)*[37] will serve to document cough, expectorations, wheezing and smoking history in every subjects. Body composition will be measured by bioimpedance and by waist circumference after a blood sample taken in the morning, in a fasting state. A six-

minute walk test (6MWT) will be performed by all subjects. After a resting period, a maximal incremental shuttle walking test will be realized in the same visit to quantify maximal exercise capacity. Measures of force and endurance of the quadriceps will be taken by magnetic stimulation of the femoral nerve before the maximal walking test. Finally, subjects will receive a portable device to monitor physical activity for a period of 7 days.

In the 2nd and 3rd visits, subjects will realize an endurance walking test at 80 % of their predetermined maximal capacity. Before each endurance test, a brondilatator or a placebo will be administered following a randomized double-blind design. Measures of force and endurance of the quadriceps will be taken by magnetic stimulation of the femoral nerve before and after endurance exercise tests. A needle biopsy of the *vastus lateralis* will be performed at the end of the third visit (see experimental plan).

General criteria

Inclusion criteria: Age > 40 yrs, smoking history (≥ 10 pack/year)

Exclusion criteria: Anticoagulation or coagulation defect, hypoxemia or exercise-induced desaturation (SpO_2 < 85%), antibiotic or oral corticosteroid therapy (end of treatment < 1 month prior to study), anti-inflammatory treatment, exacerbation (< 3 months), myopathy, neuromuscular or locomotor diseases, recent cancer, unstable cardiac condition, asthma.

Specific criteria

Inclusion criteria :

❶ Symptomatic GOLD stage I COPD: (GOLD I S_x)

☑ FEV_1/FVC < 70 % (post BD*), FEV_1 > 80 % of predicted

☑ Dyspnea symptoms: score ≥ 2/5 on *Medical Research Council* dyspnea scale (MRC)[36]

---

❷ Asymptomatic GOLD stage I COPD: (GOLD I AS_x)

☑ FEV_1/FVC < 70 % (post BD*), FEV_1 > 80 % of predicted

☑ MRC dyspnea score < 2/5

---

❸ Control group

☑ FEV_1/FVC > 70 % of predicted (post BD*), FEV_1 > 80 % of predicted

* BD : Bronchodilation

Experimental plan

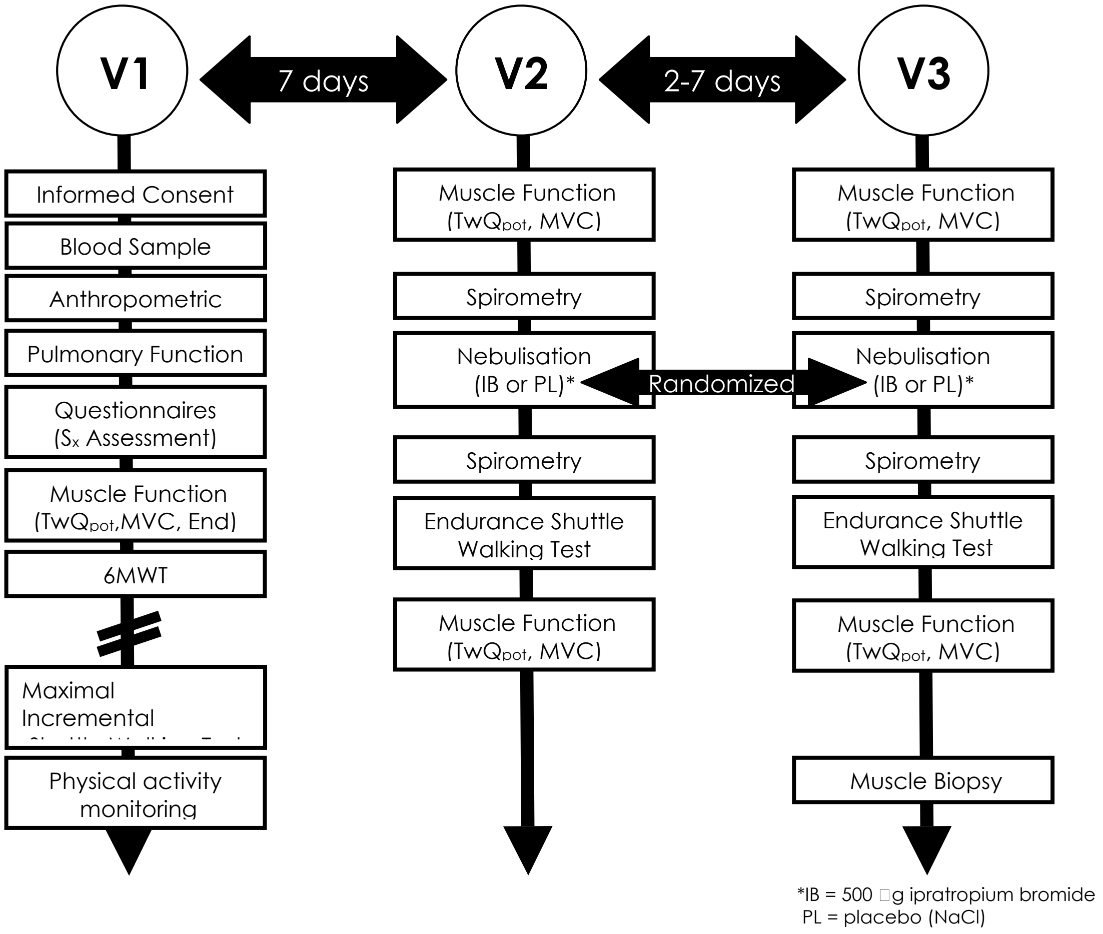

Definition of abbreviations: S_x = symptoms; TwQ_{pot} = potentiated twitch tension of the quadriceps; MVC = maximal voluntary contraction; End = endurance of the quadriceps; 6MWT = six-minute walk test.

Bronchodilation

**1st visit**	**2nd et 3rd visits (randomised order)**
Salbutamol 400 µg (*Ventolin*[MD])	Ipratropium bromide 500 [illegible]g (*Atrovent*[MD])
	or
	Placebo (NaCl)

Pulmonary function test

In the initial visit, respiratory flow, pulmonary volumes and DL_{CO} (*Spirometer, Vmax, Sensormedics, US*) will be evaluated before and after bronchodilation [*400 µg,* Salbutamol (*Ventolin*^{MD})]. In the 2nd and the 3rd visits, a spirometry will be obtained before the endurance shuttle walking test: measures will be taken at baseline and 20 min after the inhalation of whether ipratropium bromide 500 µg (*Atrovent*^{MD}) or placebo (normal saline). Importantly, subjects already following a bronchodilator treatment will be asked to interrupt their medication according to the following schedule: short-acting β2-agonists and short-acting anticholinergics, 6 hours before, long-acting β2-agonists (including fixed combination of long-acting β2-agonists and corticosteroids), 24 hours before, and theophyllines, 48 before. Due to long wash out period, patients on tiotropium will be switched to ipratropium four times a day, two weeks before the study. These subjects will then be able to follow the regular medication weaning procedures. If any short acting bronchodilator has to be taken as rescue medication within 6 hours of the study procedure, the evaluation visit will have to be rescheduled

Anthropometric measures

The weight and height will be measured to determine the body-mass index (BMI). Additionally, waist circumference and body composition via bioimpedance (*InBody 520,* Derwent Healthcare Ltd*, UK*) will be measured.

Symptomatology

The MRC dyspnea scale[36] will be the gold standard to determine whether COPD patients are symptomatic or asymptomatic. An MRC dyspnea score ≥ 2 will be define as symptomatic. The MRC scale represents a simple and validated way to quantify the impact of dyspnea on health condition[1] and predicts mortality

risk[38]. Patients presenting spirometric criteria of a GOLD stage I COPD without previous diagnosis will be evaluated by pneumologist to confirm the presence of a COPD.

The *ATS-DLD-78* questionnaire[37] will be used in order to document symptoms of chronic bronchitis (cough, expectorations, wheezing, frequency of symptoms) and to detail the respective smoking history. Finally, the *Baseline Dyspnea Index*[39], a questionnaire assessing dyspnea related to daily living activities, will be used to quantify the dyspnea degree on a scale of 0 to 12 in every subjects.

Blood and muscle samples

Prior to the different tests at the first visit, a blood sample will be taken (in a fasting condition) in order to measure hs-CRP and IL-6 levels. Levels of hs-CRP will be obtained after blood analysis by biochemistry laboratory of Hôpital Laval, while IL-6 concentration will be obtained via an ELISA analysis in our research group laboratory. Muscle oxidative stress will be quantified from the *vastus lateralis* biopsy by lipid peroxidation and oxidized proteins[35].

Maximal exercise test

During the initial visit, a maximal incremental shuttle walking test will be realized in an enclosed corridor on a flat 10-meters-long course. Patients will be asked to walk around two cones, each positioned 0.5 m from either end, following the rhythm dictated by the audio signal. Walking speed will initially be set at 0.5 meters/seconds and will subsequently increase by 0.17 meters/seconds every minute until the patients reached a symptom-limited maximum[21;40]. This test has already been validated among a COPD population[40].

The subject will breathe through a facemask, related to a portable gas exchange analyzer device (*Oxycon Mobile, Viasys Healthcare, Jaeger, Germany*) to monitor precisely (breath-by-breath) ventilatory and metabolic parameters. Ventilation ($\dot{V}_E$), oxygen consumption ($\dot{V}O_2$), CO_2 output ($\dot{V}CO_2$) and respiratory exchange ratio (RER) will be monitored and subsequently analyzed. Oxygen pulse saturation (SpO_2) will be measured at baseline and during exercise using a pulse oxymeter (*OSM2 Hexoximeter; Radiometer, Copenhagen, Denmark*). Blood pressure will be measured manually at baseline and at end-exercise. Values of dyspnea and leg fatigue will be taken at baseline, every 2-min during exercise and at end-exercise using the *modified Borg scale*[41]. Finally, an electrocardiogram (*JECG 12ch, Viasys Healthcare, Jaeger, Germany*) will be used to monitor cardiac activity during the exercise tests. Tests will be performed at Hôpital Laval and a direct medical supervision will be available for maximal exercise walking tests.

Six-minute walk test (6MWT)

The test will be performed in an enclosed corridor on a flat 30-meters-long course between two cones. Subjects will be asked to walk the greatest distance in a period of 6 minutes, with or without pause. The test will follow the procedures established by the *American Thoracic Society*[42]. Blood pressure, SpO_2, heart rate and perceived effort on the *modified Borg scale*[41] (dyspnea, leg fatigue) will be taken at baseline, at every minute and at the end of the test.

Endurance shuttle walking test

The endurance shuttle walking test will be performed in the same environment as the maximal shuttle walking test. After a 2-min warm-up period, walking speed will be set to an intensity corresponding to 80 % of $\dot{V}O_2$max, predetermined during the maximal shuttle walking test[43]. Before each endurance

test, subjects will be strongly encouraged to walk as long as possible. However, no encouragement will be provided to the subjects during the test.

Similarly to the maximal test, gas exchange and cardiac activity will be recorded during endurance walking tests (as described above). In addition, the dyspnea and leg fatigue measured by the *modified Borg scale*[41] will be recorded before, during (1-min interval) and after the endurance test. Measures of inspiratory capacity will be done at baseline and every 2-min interval during the walking test to quantify dynamic hyperinflation[44]. Finally, measures of blood pressure will be taken at baseline and at end-exercise.

Magnetic stimulation and maximal voluntary contraction

Magnetic stimulation of the femoral nerve allows objective measures of muscle fatigue: studies by Polkey and colleagues[45] have demonstrated that supramaximal twitch tension of the quadriceps (TwQ_{pot}) is a non-painful and reproducible technique. Magnetic stimulation has been validated in COPD patients and is, for several years, used among our research group[21;22;46]. Measures of TwQ_{pot} will be taken following a muscle potentiation by a brief maximal voluntary contraction (MVC) because of an improved sensibility and validity of the measure[47]. In addition, the access to a synchronized magnetic stimulator (*Magstim 200 monopulse Bistim; Magstim Co. Ltd., Whitland, Dyfed, Wales, UK*) adds to the stability of the TwQ_{pot} measures. Finally, measures of MVC of the quadriceps will be performed using the surimposed twitch technique[48]. This method increases the stability of the MVC measures.

Muscle endurance

Muscle endurance will be evaluated following the protocol previously elaborated by Allaire and colleagues in our research laboratory[49]. Subjects will be asked to maintain an isometric quadriceps tension corresponding to 60 % of their

predetermined MVC, and this to exhaustion. A visual feedback of the force will be displayed on a computer screen and will be used to help the subjects to modulate their force throughout the muscular endurance test. Subjects will be strongly encouraged to maintain the relative force required. The criterion to end the test will be a relative force output lower than 50 % of the MVC. Finally, surface electromyography (EMG) will be monitored on the *vastus lateralis* during the muscle endurance protocol following the modality already reported and validated in our research laboratory[49;50]. The isometric quadriceps tension that will be selected in healthy subjects will be paired with that of patients with COPD so that the mean isometric quadriceps tension will be equal in patients with COPD and healthy subjects.

Physical activity monitoring

Physical activity will be monitored on a 7-days period via a portable device (*SenseWear® ArmBand, Bodymedia inc., Pittsburgh, USA*). This technology allows us to quantify precisely the energy expenditure and the numbers of steps on a daily basis. It is also possible to characterize the intensity of the physical activity as it is analyzed in three main categories: light intensity (< 3 METS), moderate intensity (3-6 METS) or high intensity (> 6 METS) [51].

Muscle biopsy

Percutaneous biopsy specimens of the *vastus lateralis* will be taken at midthigh, as described by Bergström[52]. After local anaesthesia, a 6 mm skin incision will be made and muscle samples will be obtained using one or two passes with the Bergström needle. The samples will be immediately frozen in liquid nitrogen or OCT (*Tissue-Tek, Miles Inc, Elkhart, IN, USA)* embedded and frozen in pre-cooled isopentane and stored at -80°C. Our research group have already completed a number of study using this particular technique[14;49;53;54]. With the muscular samples, we will characterize fiber typing, fiber type surface

area, capillarity and the activity of certain muscular enzymes (citrate synthase[CS], 3-hydroxyacyl CoA déshydrogénase[HADH], phosphofructokinase [PFK]).

Feasibility

The sample size aimed for this project is 60 patients (20 subjects per subgroup). This sample size is based on our previous experience with a study evaluating the sensibility to bronchodilation of different exercise modalities (walking vs. cycling) in moderate to severe patients with COPD[21]. However, because our study consists in a pilot project and because limited data are available on the topic, the sample size could be readjusted during the study.

The greatest challenge of the present investigation surely resides in the identification and the recruitment of GOLD stage I COPD patients, a population that rarely attend the hospital. To do so, recruitment will be made via publicity in the newspapers and in public areas, aiming ex-smokers or active smokers aged of more than 40 years old. To identify 40 patients GOLD stage I COPD patients, we predict we will need to evaluate approximately 200 patients on the basis of our inclusion criteria. This work will be realized in collaboration with the staff of the research laboratory.

Data analysis

Inter and intra groups differences for the measures of exercise tolerance, response to bronchodilation, oxidative stress, systemic inflammation (hs-CRP, IL-6) and muscle function (force, endurance, enzymatic profile, fiber typing, capillarity) will be analyzed by a two-way ANOVA. The relationship between exercise tolerance, peripheral muscular function, systemic inflammation and physical activity levels will be evaluated by Pearson correlations. Endurance time

will be defined as the length of the endurance shuttle walking test, excluding the warm-up period. A $p < 0.05$ level of statistical significance will be used for all analyses.

Perspectives

This project could eventually lead to a better comprehension of the first manifestations of COPD. To this extent, we believe that by improving our knowledge on the origin of functional impacts of the disease, it will ultimately be possible to provide an optimal intervention for the patients at an early stage of the disease.

References

1. Rabe, K. F., S. Hurd, A. Anzueto, P. J. Barnes, S. A. Buist, P. Calverley, Y. Fukuchi, C. Jenkins, R. Rodriguez-Roisin, W. C. van, and J. Zielinski. 2007. Global strategy for the diagnosis, management, and prevention of chronic obstructive pulmonary disease: GOLD executive summary. *Am.J.Respir.Crit Care Med.* 176:532-555.

2. Pellegrino, R., G. Viegi, V. Brusasco, R. O. Crapo, F. Burgos, R. Casaburi, A. Coates, C. P. van der Grinten, P. Gustafsson, J. Hankinson, R. Jensen, D. C. Johnson, N. MacIntyre, R. McKay, M. R. Miller, D. Navajas, O. F. Pedersen, and J. Wanger. 2005. Interpretative strategies for lung function tests. *Eur.Respir.J.* 26:948-968.

3. Bridevaux, P. O., M. W. Gerbase, N. M. Probst-Hensch, C. Schindler, J. M. Gaspoz, and T. Rochat. 2008. Long-term decline in lung function, utilisation of care and quality of life in modified GOLD stage 1 COPD. *Thorax* 63:768-774.

4. Ekberg-Aronsson, M., K. Pehrsson, J. A. Nilsson, P. M. Nilsson, and C. G. Lofdahl. 2005. Mortality in GOLD stages of COPD and its dependence on symptoms of chronic bronchitis. *Respir.Res.* 6:98.

5. Mannino, D. M., D. E. Doherty, and B. A. Sonia. 2006. Global Initiative on Obstructive Lung Disease (GOLD) classification of lung disease and mortality: findings from the Atherosclerosis Risk in Communities (ARIC) study. *Respir.Med.* 100:115-122.

6. Lacasse, Y., L. Brosseau, S. Milne, S. Martin, E. Wong, G. H. Guyatt, and R. S. Goldstein. 2002. Pulmonary rehabilitation for chronic obstructive pulmonary disease. *Cochrane.Database.Syst.Rev.*CD003793.

7. Oga, T., K. Nishimura, M. Tsukino, S. Sato, T. Hajiro, and M. Mishima. 2005. Exercise capacity deterioration in patients with COPD: longitudinal evaluation over 5 years. *Chest* 128:62-69.

8. Ofir, D., P. Laveneziana, K. A. Webb, Y. M. Lam, and D. E. O'Donnell. 2008. Mechanisms of dyspnea during cycle exercise in symptomatic patients with GOLD stage I chronic obstructive pulmonary disease. *Am.J.Respir.Crit Care Med.* 177:622-629.

9. Bestall, J. C., E. A. Paul, R. Garrod, R. Garnham, P. W. Jones, and J. A. Wedzicha. 1999. Usefulness of the Medical Research Council (MRC) dyspnoea scale as a measure of disability in patients with chronic obstructive pulmonary disease. *Thorax* 54:581-586.

10. Gosselink, R., T. Troosters, and M. Decramer. 1996. Peripheral muscle weakness contributes to exercise limitation in COPD. *Am.J.Respir.Crit Care Med.* 153:976-980.

11. Maltais, F. 2001. [Peripheral muscles in chronic obstructive pulmonary disease]. *Rev.Mal Respir.* 18:S24-S26.

12. Bernard, S., P. Leblanc, F. Whittom, G. Carrier, J. Jobin, R. Belleau, and F. Maltais. 1998. Peripheral muscle weakness in patients with chronic obstructive pulmonary disease. *Am.J.Respir.Crit Care Med.* 158:629-634.

13. Hamilton, A. L., K. J. Killian, E. Summers, and N. L. Jones. 1995. Muscle strength, symptom intensity, and exercise capacity in patients with cardiorespiratory disorders. *Am.J.Respir.Crit Care Med.* 152:2021-2031.

14. Whittom, F., J. Jobin, P. M. Simard, P. Leblanc, C. Simard, S. Bernard, R. Belleau, and F. Maltais. 1998. Histochemical and morphological characteristics of the vastus lateralis muscle in patients with chronic obstructive pulmonary disease. *Med.Sci.Sports Exerc.* 30:1467-1474.

15. Mador, M. J., O. Deniz, A. Aggarwal, and T. J. Kufel. 2003. Quadriceps fatigability after single muscle exercise in patients with chronic obstructive pulmonary disease. *Am.J.Respir.Crit Care Med.* 168:102-108.

16. Maltais, F., A. A. Simard, C. Simard, J. Jobin, P. Desgagnes, and P. Leblanc. 1996. Oxidative capacity of the skeletal muscle and lactic acid kinetics during exercise in normal subjects and in patients with COPD. *Am.J.Respir.Crit Care Med.* 153:288-293.

17. Jobin, J., F. Maltais, J. F. Doyon, P. Leblanc, P. M. Simard, A. A. Simard, and C. Simard. 1998. Chronic obstructive pulmonary disease: capillarity and fiber-type characteristics of skeletal muscle. *J.Cardiopulm.Rehabil.* 18:432-437.

18. Doucet, M., R. Debigare, D. R. Joanisse, C. Cote, P. Leblanc, J. Gregoire, J. Deslauriers, R. Vaillancourt, and F. Maltais. 2004. Adaptation of the diaphragm and the vastus lateralis in mild-to-moderate COPD. *Eur.Respir.J.* 24:971-979.

19. Anthonisen, N. R., J. E. Connett, J. P. Kiley, M. D. Altose, W. C. Bailey, A. S. Buist, W. A. Conway, Jr., P. L. Enright, R. E. Kanner, P. O'Hara, and . 1994. Effects of smoking intervention and the use of an inhaled anticholinergic bronchodilator on the rate of decline of FEV1. The Lung Health Study. *JAMA* 272:1497-1505.

20. O'Donnell, D. E., P. Laveneziana, J. Ora, K. A. Webb, Y. M. Lam, and D. Ofir. 2008. Evaluation of Acute Bronchodilator Reversibility in Symptomatic GOLD Stage I COPD. *Thorax*.

21. Pepin, V., D. Saey, F. Whittom, P. Leblanc, and F. Maltais. 2005. Walking versus cycling: sensitivity to bronchodilation in chronic obstructive pulmonary disease. *Am.J.Respir.Crit Care Med.* 172:1517-1522.

22. Saey, D., R. Debigare, P. Leblanc, M. J. Mador, C. H. Cote, J. Jobin, and F. Maltais. 2003. Contractile leg fatigue after cycle exercise: a factor limiting exercise in patients with chronic obstructive pulmonary disease. *Am.J.Respir.Crit Care Med.* 168:425-430.

23. Pitta, F., T. Troosters, M. A. Spruit, V. S. Probst, M. Decramer, and R. Gosselink. 2005. Characteristics of physical activities in daily life in chronic obstructive pulmonary disease. *Am.J.Respir.Crit Care Med.* 171:972-977.

24. Prefaut, C. 1995. [Physical activity and cardiorespiratory equilibrium]. *Bull.Acad.Natl.Med.* 179:1461-1469.

25. Spencer, S., P. M. Calverley, B. P. Sherwood, and P. W. Jones. 2001. Health status deterioration in patients with chronic obstructive pulmonary disease. *Am.J.Respir.Crit Care Med.* 163:122-128.

26. Myers, J., M. Prakash, V. Froelicher, D. Do, S. Partington, and J. E. Atwood. 2002. Exercise capacity and mortality among men referred for exercise testing. *N.Engl.J Med.* 346:793-801.

27. Watz, H., B. Waschki, C. Boehme, M. Claussen, T. Meyer, and H. Magnussen. 2008. Extrapulmonary effects of chronic obstructive pulmonary disease on physical activity: a cross-sectional study. *Am.J Respir.Crit Care Med.* 177:743-751.

28. Decramer, M., S. Rennard, T. Troosters, D. W. Mapel, N. Giardino, D. Mannino, E. Wouters, S. Sethi, and C. B. Cooper. 2008. COPD as a lung disease with systemic consequences--clinical impact, mechanisms, and potential for early intervention. *COPD.* 5:235-256.

29. Dahl, M., J. Vestbo, P. Lange, S. E. Bojesen, A. Tybjaerg-Hansen, and B. G. Nordestgaard. 2007. C-reactive protein as a predictor of prognosis in chronic obstructive pulmonary disease. *Am.J Respir.Crit Care Med.* 175:250-255.

30. Man, S. F., J. E. Connett, N. R. Anthonisen, R. A. Wise, D. P. Tashkin, and D. D. Sin. 2006. C-reactive protein and mortality in mild to moderate chronic obstructive pulmonary disease. *Thorax* 61:849-853.

31. Agusti, A. G., A. Noguera, J. Sauleda, E. Sala, J. Pons, and X. Busquets. 2003. Systemic effects of chronic obstructive pulmonary disease. *Eur.Respir.J.* 21:347-360.

32. American Thoracic Society (ATS) and European Respiratory Society (ERS). 1999. Skeletal muscle dysfunction in chronic obstructive pulmonary disease. A statement of the ATS and ERS. *Am.J.Respir.Crit Care Med.* 159:S1-40.

33. Reid, M. B., D. S. Stokic, S. M. Koch, F. A. Khawli, and A. A. Leis. 1994. N-acetylcysteine inhibits muscle fatigue in humans. *J.Clin.Invest* 94:2468-2474.

34. Reid, M. B. 2001. COPD as a muscle disease. *Am.J.Respir.Crit Care Med.* 164:1101-1102.

35. Couillard, A., F. Maltais, D. Saey, R. Debigare, A. Michaud, C. Koechlin, P. Leblanc, and C. Prefaut. 2003. Exercise-induced quadriceps oxidative stress and peripheral muscle dysfunction in patients with chronic obstructive pulmonary disease. *Am.J.Respir.Crit Care Med.* 167:1664-1669.

36. Fletcher, C. M., P. C. Elmes , A. S. Fairbairn , and C. H. Wood . 1959. The significance of respiratory symptoms and the diagnosis of chronic bronchitis in a working population. *Br.Med.J.* 2:257-266.

37. Ferris, B. G. 1978. Epidemiology Standardization Project (American Thoracic Society). *Am.Rev.Respir.Dis.* 118:1-120.

38. Nishimura, K., T. Izumi, M. Tsukino, and T. Oga. 2002. Dyspnea is a better predictor of 5-year survival than airway obstruction in patients with COPD. *Chest* 121:1434-1440.

39. Mahler, D. A., D. H. Weinberg, C. K. Wells, and A. R. Feinstein. 1984. The measurement of dyspnea. Contents, interobserver agreement, and physiologic correlates of two new clinical indexes. *Chest* 85:751-758.

40. Singh, S. J., M. D. Morgan, S. Scott, D. Walters, and A. E. Hardman. 1992. Development of a shuttle walking test of disability in patients with chronic airways obstruction. *Thorax* 47:1019-1024.

41. Borg, G. A. 1982. Psychophysical bases of perceived exertion. *Med.Sci.Sports Exerc.* 14:377-381.

42. American Thoracic Society (ATS). 2002. ATS statement: guidelines for the six-minute walk test. *Am.J.Respir.Crit Care Med.* 166:111-117.

43. Revill, S. M., M. D. Morgan, S. J. Singh, J. Williams, and A. E. Hardman. 1999. The endurance shuttle walk: a new field test for the assessment of endurance capacity in chronic obstructive pulmonary disease. *Thorax* 54:213-222.

44. O'Donnell, D. E., M. Lam, and K. A. Webb. 1998. Measurement of symptoms, lung hyperinflation, and endurance during exercise in chronic obstructive pulmonary disease. *Am.J.Respir.Crit Care Med.* 158:1557-1565.

45. Polkey, M. I., D. Kyroussis, C. H. Hamnegard, G. H. Mills, M. Green, and J. Moxham. 1996. Quadriceps strength and fatigue assessed by magnetic stimulation of the femoral nerve in man. *Muscle Nerve* 19:549-555.

46. Saey, D., A. Michaud, A. Couillard, C. H. Cote, M. J. Mador, P. Leblanc, J. Jobin, and F. Maltais. 2005. Contractile fatigue, muscle morphometry, and blood lactate in chronic obstructive pulmonary disease. *Am.J.Respir.Crit Care Med.* 171:1109-1115.

47. Kufel, T. J., L. A. Pineda, and M. J. Mador. 2002. Comparison of potentiated and unpotentiated twitches as an index of muscle fatigue. *Muscle Nerve* 25:438-444.

48. Strojnik, V. and P. V. Komi. 1998. Neuromuscular fatigue after maximal stretch-shortening cycle exercise. *J.Appl.Physiol* 84:344-350.

49. Allaire, J., F. Maltais, J. F. Doyon, M. Noel, P. Leblanc, G. Carrier, C. Simard, and J. Jobin. 2004. Peripheral muscle endurance and the oxidative profile of the quadriceps in patients with COPD. *Thorax* 59:673-678.

50. Saey, D., C. H. Cote, M. J. Mador, L. Laviolette, P. Leblanc, J. Jobin, and F. Maltais. 2006. Assessment of muscle fatigue during exercise in chronic obstructive pulmonary disease. *Muscle Nerve* 34:62-71.

51. Saey, D., Mainguy, V., Maltais, F., and Provencher, S. Assessment of daily life physical activity in patients with pulmonary hypertension. *Am.J.Respir.Crit Care Med*. 177, A918. 2008.

52. Bergström, J. Muscle electrolytes in man. Determination by neutron activation analysis on needle biopsy specimens. A study on normal subjects, kidney patients and patients with chronic diarrhoea. *Scand J Clin Lab Invest* 14[1], 110. 1962.

53. Maltais, F., M. J. Sullivan, P. Leblanc, B. D. Duscha, F. H. Schachat, C. Simard, J. M. Blank, and J. Jobin. 1999. Altered expression of myosin heavy chain in the vastus lateralis muscle in patients with COPD. *Eur.Respir.J.* 13:850-854.

54. Maltais, F., P. Leblanc, F. Whittom, C. Simard, K. Marquis, M. Belanger, M. J. Breton, and J. Jobin. 2000. Oxidative enzyme activities of the vastus lateralis muscle and the functional status in patients with COPD. *Thorax* 55:848-853.
