# Supplementary material for: Cluster Analysis in Patients with GOLD 1 Chronic Obstructive Pulmonary Disease
Source: PLoS One. 2015 Apr 23;10(4):e0123626. doi: 10.1371/journal.pone.0123626 (PMC4407903; doi:10.1371/journal.pone.0123626)
Supplement: S2 Protocol — (PDF) [file pone.0123626.s003.pdf]

Clinical Trial Protocol Revision B (Including Amendments No. 1 and 2)

Doc. No.: U09-3881-01

**BI Trial No.:**	205.440<br>Including Amendments No. 1 and 2
**Investigational Product:**	Spiriva®, tiotropium bromide
**Title:**	A randomized, double-blind, placebo-controlled, 6-week, cross-over study to examine the effects of tiotropium (Spiriva® HandiHaler®, 18 μg once daily) on lung dynamic hyperinflation and physical exercise endurance in patients with early stage Chronic Obstructive Pulmonary Disease (COPD)
**Clinical Phase:**	IV
**Trial Clinical Monitor:**	Heather Bennett, BSRC, RRT<br>Boehringer-Ingelheim Pharmaceuticals, Inc.<br>900 Ridgebury Rd, PO Box 368<br>Ridgefield, CT 06877 (USA)<br>Telephone: (203) 798-5578 Fax: (203) 837-5578
**Co-ordinating Investigator:**	Richard Casaburi, PhD, MD<br>Los Angeles Biomedical Research Institute<br>Harbor-UCLA Medical Center<br>1124 W. Carson St., Building J4<br>Torrance, CA 90502<br>Telephone: (310) 222-8200 Fax: (310) 222-8249
**Date of Protocol:**	**Original:** 10 December 2009<br>**Revision A:** 25 February 2010<br>**Revision B:** 02 September 2010
**Planned Dates of Trial:**	February 2010 to April 2011

Proprietary confidential information.
© 2010 Boehringer Ingelheim International GmbH or one or more of its affiliated companies. All rights reserved.
This document may not - in full or in part - be passed on, reproduced, published or otherwise used without prior written permission.

CLINICAL TRIAL PROTOCOL REVISION PAGE

I herewith certify that this Clinical Trial Protocol Revision (Revision B) gives an accurate and complete revision of the protocol, including Amendments Nos. 1, 2.

Trial Clinical Monitor   02Sep10   [signature]

Date

Heather Bennett
Boehringer-Ingelheim Pharmaceuticals, Inc.

The official documents are the original protocol and applicable amendments. This unofficial copy of the protocol does not require signature, and therefore, the signature pages remain blank.

CLINICAL TRIAL PROTOCOL SYNOPSIS

**Name of company:**<br><br>Boehringer Ingelheim	**Tabulated Trial Protocol**	
**Name of finished product:**<br><br>Spiriva® HandiHaler®		
**Name of active ingredient:**<br><br>Tiotropium bromide		

**Protocol date**	**Trial number**	**Planned trial period**
10 DEC 2009	205.440	February 2010-April 2011

**Title of trial:**	A randomized, double-blind, placebo-controlled, 6-week, cross-over study to examine the effects of tiotropium (Spiriva® HandiHaler®, 18 µg once daily) on lung dynamic hyperinflation and physical exercise endurance in patients with early stage Chronic Obstructive Pulmonary Disease (COPD)
**Co-ordinating Investigator:**	Richard Casaburi, PhD, MD
**Trial sites :**	Multi-centre trial
**Clinical phase:**	IV
**Objectives:**	To determine the effects of tiotropium on lung dynamic hyperinflation due to airflow limitation as well as on symptom limited exercise compared to placebo in symptomatic patients with early stage COPD.<br>An additional objective is to characterize the mechanisms of exercise limitation and describe daily physical activity in patients with early stage COPD compared to Age / Gender Matched Controls.
**Methodology:**	Randomized, double-blind, two-period cross-over trial: Two six-week treatment periods are separated by four-week wash-out.
**No. of patients:**	
**total:**	A suitable number of early stage COPD subjects will be enrolled to ensure 124 COPD subjects are randomized<br>In addition, 100 Age / Gender Matched Controls will be enrolled (matched to the first 100 randomized early stage COPD subjects).
**each treatment:**	124 symptomatic early stage COPD subjects randomized to ensure 100 completed (cross-over design)
**Diagnosis and main criteria for inclusion**	**For COPD subjects:** Male or female, ≥40 years of age, smoking history ≥10 pack-years, symptomatic defined as baseline dyspnea index focal score ≤9 and / or daily cough with production of sputum for three months per year during at least two consecutive years, diagnosis of early stage COPD according to: post-bronchodilator FEV_1/FVC ratio <70%., FEV_1 ≥50% post-bronchodilator predicted normal, and a decreased Inspiratory Capacity during exercise.<br>**For Age / Gender Matched Controls:** Male or female, ≥40 years of age, non-smoker, with no significant diseases.

**Name of company:**<br><br>Boehringer Ingelheim	**Tabulated**<br>**Trial Protocol**	
**Name of finished product:**<br><br>Spiriva® HandiHaler®		
**Name of active ingredient:**<br><br>Tiotropium bromide		

**Protocol date**<br>10 DEC 2009	**Trial number**<br>205.440	**Planned trial period**<br>February 2010-April 2011

**Test product :**	Tiotropium bromide (Spiriva® HandiHaler®)
**dose:**	18 µg once daily
**mode of admin. :**	Oral inhalation with HandiHaler®
**Reference therapy:**	Placebo (HandiHaler®)
**dose:**	Not applicable
**mode of admin. :**	Oral inhalation with HandiHaler®
**Duration of treatment:**	Two six-week treatment periods, separated by four-week wash-out
**Criteria for efficacy:**	Primary:<br>The difference from baseline to Week 6 post dose in Inspiratory Capacity (IC) during constant work rate treadmill exercise test measured at isotime.<br><br>Key Secondary:<br>Difference from baseline to Week 6 post dose:<br>• Dyspnea intensity during physical exercise: Modified Borg Scale of breathing discomfort (at isotime and isoventilation).<br>• Constant Work Rate Exercise Duration: calculated as the length of time of the exercise period.<br><br>Other Secondary:<br>Difference from baseline to Week 6 post dose:<br>• Percentage of patients with respiratory limitation (as defined by Breathing Reserve <15 L/min)<br>• Shift in locus of symptom limitation (breathing limited vs. leg limited)<br>• Ventilatory and respiratory mechanics, metabolic parameters, breathing pattern and operating lung volumes during constant work rate exercise as described in Section 5.1.2<br>• Dyspnea intensity at end exercise<br>• FEV_1 (pre-dose value measured approximately 24 hours after the previous dose of study drug and 90 minutes post-study drug administration)<br>• Rescue medication use
**Criteria for safety:**	Adverse events, vital signs

**Name of company:**<br><br>Boehringer Ingelheim	**Tabulated**<br>**Trial Protocol**	
**Name of finished product:**<br><br>Spiriva® HandiHaler®		
**Name of active ingredient:**<br><br>Tiotropium bromide		

**Protocol date**<br>10 DEC 2009	**Trial number**<br>205.440	**Planned trial period**<br>February 2010-April 2011
**Statistical methods:**	The primary endpoint and continuous secondary endpoints will be analyzed using a mixed effect model for the change from baseline after six weeks of treatment, with terms of sequence, treatment, period as fixed effects, baseline as a covariate, and subject within sequence as a random effect. The locus of symptom limitation will be summarized using frequency tables and shift table. The other endpoints comparing COPD patients with age/gender matched controls will be summarized descriptively, with 95% confidence interval for continuous variables. Safety endpoints will be summarized descriptively.	

FLOW CHART

	RUN-IN			TREATMENT				FOLLOW-UP PHONE CALL VISIT
	Visit 0[**]	Visit 1***	Visit 2***	Visit 3	Visit 4	Visit 5	Visit 6	Visit 7
Study Week		-2	-1	1	6	10	16	20
Study Day	-30 to -14	-14 to -7	-7 to -3	1	42 (± 3)	70 (± 3)	112 (± 3)	+30 days after Visit 6 (± 2)
Informed Consent[*]	X							
Medical History/Demographics	X							
Pregnancy Test		X						
Smoking status[b]	X	X	X	X	X	X	X	X
Inclusion/Exclusion Criteria	X	X		X[a]				
Physical Examination		X					X	
BP & Pulse (Seated)		X	X	X	X	X	X	
12-Lead ECG (Resting)		X						
Check Patient Daily Record / Rescue albuterol use assessment[b]		X	X	X	X	X	X	
Washout compliance phone call (up to three days prior) [b]		X	X	X	X	X	X	
Medication Washout Assessment[b]		X	X	X	X	X	X	
Accelerometer Training/ Dispense[c]		X						
Collect Accelerometer[c]				X				
Dyspnea Symptom Scores: (BDI)		X						
Body plethysmography & DLco		X						
Physical Function Questionnaires: *WPAI-GH, FACIT short form, and VSAQ*		X						
Spirometry (pre & post open label albuterol)		X						
Spirometry (pre study medication ONLY)				X		X		
Spirometry (pre & post study medication)					X		X	
Incremental exercise test		X						
Constant work rate exercise test (pre study medication)			X	X		X		
Constant work rate exercise test (post study medication					X		X	
Adverse Events and Concomitant Therapy	X	X	X	X	X	X	X	X
HandiHaler® Training				X [d]				
Randomization				X [d]				
Dispense Trial Medication				X [d]		X		
Termination of Trial Medication					X		X	
Trial Completion								X

= Procedures performed by both COPD and Age/ Gender Matched Control Subjects

* ICF must be signed prior to any study related activities (including wash-out)
** Please note: Visit 0 for Early Stage COPD can be up to four weeks prior to Visit 3 as needed for medication washout. Visit 0 for potential Age / Gender Matched Controls can be as long as needed for the protocol and does not need to be restricted to four weeks prior to Visit 3.
*** There must be a minimum of three days between Visits 1 and 2.
a. Early stage COPD Subjects need to qualify for randomization at Visit 3
b. Not applicable for Age / Gender Matched Control Subjects
c. Accelerometer must be worn for two consecutive weeks during the run-in period between Visit 1 and Visit 3. The accelerometer is to be removed during Visit 2.
d. Early stage COPD subjects only are randomized, dispensed study medication and continue in the protocol past Visit 3

TABLE OF CONTENTS

Page

TITLE PAGE 1
CLINICAL TRIAL PROTOCOL REVISION PAGE 2
CLINICAL TRIAL PROTOCOL SYNOPSIS 3
FLOW CHART 6
TABLE OF CONTENTS 8
ABBREVIATIONS 11
1. INTRODUCTION 13
1.1 MEDICAL BACKGROUND 13
1.2 DRUG PROFILE 14
1.3 RATIONALE FOR PERFORMING THE TRIAL 14
1.4 BENEFIT - RISK ASSESSMENT 15
CLINICAL TRIAL PROTOCOL REVISION PAGE 16
2. TRIAL OBJECTIVES 17
2.1 GENERAL AIM - OBJECTIVE 17
2.2 PRIMARY ENDPOINT 17
2.3 KEY SECONDARY ENDPOINTS 17
2.3.1 Other secondary endpoints 17
2.4 OTHER ENDPOINTS 18
2.5 SAFETY ENDPOINTS 18
3. DESCRIPTION OF DESIGN AND TRIAL POPULATION 19
3.1 OVERALL TRIAL DESIGN AND PLAN - DESCRIPTION 19
3.2 DISCUSSION OF TRIAL DESIGN, INCLUDING THE CHOICE OF CONTROL GROUP 20
3.3 SELECTION OF TRIAL POPULATION 21
3.3.1 Inclusion criteria for all trial subjects 22
3.3.2 Exclusion criteria for all trial subjects 23
4. TREATMENTS 26
4.1 TREATMENTS TO BE ADMINISTERED 26
4.1.1 Identity of investigational products 26
4.1.2 Method of assigning patients to treatment groups 26
4.1.3 Selection of doses in the trial 27
4.1.4 Selection and timing of doses for each patient 27
4.1.5 Blinding 28
4.1.6 Packaging, labelling, and re-supply 28
4.1.7 Storage conditions 29
4.2 CONCOMITANT THERAPY 29

Page

4.2.1 Rescue medication and additional treatment 29
4.2.2 Restrictions 30
4.3 TREATMENT COMPLIANCE 31
5. OBSERVATIONS 32
5.1 EFFICACY - CLINICAL PHARMACOLOGY OR PHARMACODYNAMICS 32
5.1.1 Primary endpoints 32
5.1.2 Secondary and other endpoints 33
5.2 SAFETY 37
5.3 OTHER 39
5.4 APPROPRIATENESS OF MEASUREMENTS 39
5.5 DRUG CONCENTRATION MEASUREMENTS - PHARMACOKINETICS 40
5.6 BIOMARKER - PHARMACODYNAMIC SAMPLING 40
5.7 PHARMACOKINETIC - PHARMACODYNAMIC RELATIONSHIP 40
5.8 DATA QUALITY ASSURANCE 40
6. INVESTIGATIONAL PLAN 42
6.1 VISIT SCHEDULE 42
6.2 TRIAL PROCEDURES AT EACH VISIT 42
6.2.1 Screening and run-in phase 42
6.2.2 Treatment phases 45
6.2.3 End of trial and follow-up (Visit 7) 48
6.2.4 End of trial (premature withdrawal) 48
6.3 REMOVAL OF PATIENTS FROM THERAPY OR ASSESSMENT 49
7. STATISTICAL METHODS AND DETERMINATION OF SAMPLE SIZE 51
7.1 STATISTICAL DESIGN / MODEL 51
7.2 NULL AND ALTERNATIVE HYPOTHESES 51
7.3 PLANNED ANALYSES 51
7.3.1 Primary analyses 52
7.3.2 Secondary analyses 52
7.3.3 Safety analyses 52
7.3.4 Interim analyses 53
7.4 HANDLING OF MISSING DATA 53
7.5 RANDOMIZATION 53
7.6 DETERMINATION OF SAMPLE SIZE 53
8. ADMINISTRATIVE MATTERS 55
8.1 ETHICS 55

Page

8.1.1 Independent Ethics Committee or Institutional Review Board 55
8.1.2 Patient Information and Informed Consent 55
8.2 RECORDS 56
8.2.1 Drug accountability 56
8.2.2 Emergency code break 56
8.2.3 Case Report Forms (CRFs) 57
8.2.4 Source documents 57
8.2.5 Direct access to source data - documents 58
8.3 QUALITY ASSURANCE AUDIT 58
8.4 PROCEDURES 58
8.4.1 Adverse events 58
8.4.2 Emergency procedures 60
8.5 RULES FOR AMENDING PROTOCOL 60
8.6 DISCONTINUATION OF THE TRIAL BY THE SPONSOR 60
8.7 STATEMENT OF CONFIDENTIALITY 61
8.8 PUBLICATION POLICY 61
9. REFERENCES 62
9.1 PUBLISHED REFERENCES 62
9.2 UNPUBLISHED REFERENCES 65
10. APPENDICES 66
10.1 PATIENT'S INSTRUCTIONS FOR USE OF THE SPIRIVA® HANDIHALER® 66
10.2 INSTRUCTINS FOR USE OF THE METERED DOSE INHALER (ALBUTEROL) 77
10.3 EXERCISE TESTS 78
10.4 BORG SCALE 84
10.4.1 Instructions for the Borg Scale: intensity of dyspnea and leg discomfort 84
10.5 LOCUS OF SYMPTOM LIMITATION QUESTIONAIRE 86
10.6 VETERANS SPECIFIC ACTIVITY QUESTIONNAIRE (VSAQ) 87
10.7 WORK PRODUCTIVITY AND ACTIVITY IMPAIRMENT QUESTIONNIRE: GENERAL HEALTH V2.0 (WPAI-GH) 88
10.8 FACIT-SHORTNESS OF BREATH AND FUNCTIONAL LIMITATION SHORT FORM 90
10.9 BASELINE DYSPNEA INDEX QUESTIONAIRE 96

ABBREVIATIONS

AE	Adverse Event
BI	Boehringer Ingelheim
BDI	Baseline Dyspnea Index
CML	Clinical Monitor Local
CRA	Clinical Research Associate
CRF/eCRF	Case Report Form / electronic Case Report Form
CTMF	Clinical Trial Master File
CTP	Clinical Trial Protocol
CTR	Clinical Trial Report
CWR	Constant Work Rate
DOC	Documentation of Change
D_LCO	Diffusing Capacity for Carbon Monoxide
ECG	Electrocardiogram
EDC	Electronic Data Capture
ET	Exercise Time
FAS	Full Analysis Set
FDA	Food and Drug Administration
FEV_1	Forced expiratory volume in one second
FRC	Functional Residual Capacity
FVC	Forced vital capacity
GCP	Good Clinical Practice
GOLD	Global Initiative for Chronic Obstructive Lung Disease
HCG	Human chorionic gonadotropin
Hg	Mercury
HR	Heart Rate
EC/IEC	(Independent) Ethics Committee
IB	Investigator's Brochure
IC	Inspiratory Capacity
ICH	International Conference on Harmonisation
IET	Incremental Exercise Test
IRB	Institutional Review Board
ISF	Investigator Site File
ITT	Intent to Treat
MDI	Metered Dose Inhaler
MVV	Maximum Voluntary Ventilation
No.	Number
PFT	Pulmonary Function Test
PPS	Per Protocol Set
RDC	Remote Data Capture
RV	Residual Volume
SAE	Serious Adverse Event
SOP	Standard Operating Procedure
SpO2	Oxygen Saturation Measured By Pulse Oximetry
SRaw	Specific Airway Resistance

SVC	Slow Vital Capacity
T_e	Expiratory Time
T_i	Inspiratory Time
TLC	Total Lung Capacity
T_{tot}	Total Respiratory Time
TSAP	Trial Statistical Analysis Plan
V_E	Ventilation Exchange
VO_2	Ventilation Oxygen Consumption
V_{max}	Maximum Ventilation
V_T	Tidal Volume

1. INTRODUCTION

1.1 MEDICAL BACKGROUND

Chronic obstructive pulmonary disease (COPD) is characterized by the presence of partially reversible airflow obstruction due to chronic bronchitis, emphysema, or both. COPD is a progressive debilitating disease that leads to significant mortality; however, it is usually not diagnosed until patients complain about exertional dyspnea and the airways obstruction is moderately advanced according to FEV_1 measurement (1).

In the treatment of stable COPD, ATS/ ERS guidelines recommend maintenance therapy for subjects with persistent symptoms to prevent and control symptoms, improve health status, and reduce the severity and frequency of exacerbations. The Global Initiative for Chronic Obstructive Lung Disease (GOLD) guidelines suggests initiating the use of long acting inhaled bronchodilators as COPD maintenance therapy based on post-bronchodilator FEV_1 (FEV_1 ≤80%) and supports bronchodilators as the first line therapeutic option for treating the disease (2). Other national guidelines clearly state that “the optimal pharmacotherapy of COPD is guided on an individual basis by assessment of level of disease severity (i.e., symptoms/disability and spirometry) and frequency of acute exacerbations” and doesn’t rely on FEV_1 alone (3). However, retrospective analyses have suggested that maintenance therapy is generally not started until subjects demonstrate severe airflow obstruction and significant symptoms (1, 4).

Symptomatic GOLD stage I COPD patients can have significant physiological abnormalities beyond a pure reduction in FEV_1. Recently, O’Donnell’s research group showed that symptomatic early stage COPD patients may develop dynamic hyperinflation during cycle ergometry exercise compared to age- and gender-matched controls (5) and that the dynamic hyperinflation and exercise related dyspnea in symptomatic GOLD stage I COPD patients can be amenable to pharmacological intervention (6). It has also been demonstrated that mild COPD patients have considerable compromised health-related quality of life (7). A study by Watz et al conducted in patients with chronic bronchitis (formerly defined as GOLD stage 0) and patients with GOLD stages I-IV COPD found that there was a gradual decrease in physical activity from patients with chronic bronchitis to GOLD stage IV patients (8).

A study conducted in Sweden (9) assessed the effects of tiotropium in patients with mild COPD (FEV_1≥60% predicted). This study demonstrated significant improvement in FEV_1 and FVC in patients treated with tiotropium, compared with placebo, from baseline to 12 weeks post treatment suggesting that tiotropium is beneficial in patients with mild COPD, although changes in a generic quality of life instrument were not observed.

Retrospective analysis of two previous controlled clinical trials using constant work rate (CWR) exercise have demonstrated that treatment with tiotropium results in significant improvement in exercise duration in patients with GOLD stage II to IV disease (10, 40). Additional retrospective analysis of these two trials demonstrates significant improvement in lung dynamic hyperinflation (peak IC) in GOLD stage II patients treated with tiotropium, compared with placebo, from baseline to six weeks post treatment.

Given the positive results of previous exercise trials assessing effects of tiotropium in moderate-to-severe COPD, this study is designed to examine the impact of tiotropium on dynamic hyperinflation and exercise limitation in early stages of the disease.

1.2 DRUG PROFILE

Tiotropium bromide (Ba 679 BR) is a non-chiral, long-acting, inhaled anticholinergic bronchodilator, with a kinetic selectivity profile for the muscarinic receptor subtypes and an increased duration of action (24 hours) compared with ipratropium (4-6 hours). Complete details of the currently available information regarding the pharmacological, pharmacokinetic, toxicological and clinical profile of tiotropium bromide are available in the current Investigator's Brochures for tiotropium bromide (which can be found in the site Investigator's Site File [ISF]).

1.3 RATIONALE FOR PERFORMING THE TRIAL

Inhaled bronchodilators are recommended as the first line therapeutic option to treat COPD. Bronchodilation can be achieved by beta2-adrenergic stimulation or cholinergic blockade, the latter being of significant importance in COPD.

Tiotropium has been shown to improve exercise tolerance during CWR cycle ergometry in two similar designed trials of 6 weeks duration in a population of patients with GOLD stage II to IV COPD (10, 41). A six-month trial documented improvements in constant speed treadmill time in a study of patients who also received pulmonary rehabilitation (11); however, the population in this study was a narrower group of patients with predominantly severe and very severe disease (GOLD stages III and IV). A 12-week tiotropium study conducted in France demonstrated improvements in trough and post dose spirometry, quality of life, exertional dyspnea, and Shuttle Walk Test distance in GOLD stage III and IV COPD (40). Data are lacking on the benefits of tiotropium on exercise tolerance in a patients with early stages of COPD who are symptomatic.

For COPD patients' relief of symptoms and improved physical function may be more important outcomes of drug therapy than improvement in FEV_1. Early stage COPD patients report greater exertional dyspnea compared to age and gender matched controls (5). Ferrer and colleagues have reported that patients with mild COPD have a substantially compromised health-related quality of life (6). In addition, Carter and colleagues have shown that patients with mild COPD have a reduced physical work capacity (12).

Optimal clinical management in early stages of COPD is not established. Patients with milder ventilatory limitations (GOLD stages I/II COPD patients) may benefit from maintenance therapy and there is limited data on exercise limitation in patients with early stage COPD who are symptomatic. This study will explore the mechanisms of exercise limitation in early stage COPD and the impact of tiotropium maintenance therapy on dynamic hyperinflation, ventilatory mechanics and breathlessness during exercise.

This study is designed to first evaluate the mechanisms of breathlessness and assess physical activity limitation in early stage COPD patients compared to age and gender matched

controls and will secondly investigate the effectiveness of treatment with tiotropium in improving dyspnea during exercise and exercise duration as a result of the bronchodilation effects of tiotropium leading to a reduction of dynamic hyperinflation in Early Stage COPD patients.

1.4 BENEFIT - RISK ASSESSMENT

Tiotropium has been utilized in clinical practice since initial marketing authorization in Europe in June 2002. In the United States tiotropium has been commercially available since May 2004. The extensive safety evaluation from the four-year UPLIFT trial demonstrated that the 18 µg tiotropium inhalation powder capsule has a well understood safety profile and has a favourable benefit-risk ratio for long term, once daily, maintenance treatment of bronchospasm associated with COPD, including chronic bronchitis and emphysema (13). A recently published pre-specified subgroup analysis in UPLIFT showed that tiotropium is an effective bronchodilator and well tolerated in GOLD stage II patients (14).

Exercise trials involving over 500 COPD patients have demonstrated improvements in exercise endurance with tiotropium and that exercise testing can be conducted safely in patients with COPD (10, 40). In the present study, patients will participate in a two-period cross-over study and will be randomized to either receive tiotropium and then placebo or placebo and then tiotropium. Given the known safety profile of tiotropium we do not anticipate an increased risk for the population selected for this study.

CLINICAL TRIAL PROTOCOL REVISION PAGE

I herewith certify that this Clinical Trial Protocol Revision (Revision B) gives an accurate and complete revision of the protocol, including Amendment Nos. 1,2.

Trial Clinical Monitor ________________ ___________________________________

Date

Heather Bennett
Boehringer-Ingelheim Pharmaceuticals, Inc.

The official documents are the original protocol and applicable amendments. This unofficial copy of the protocol does not require signature, and therefore, the signature pages remain blank.

2. TRIAL OBJECTIVES

2.1 GENERAL AIM - OBJECTIVE

The primary objective of this study is to determine the effects of tiotropium on lung dynamic hyperinflation (as measured by Inspiratory Capacity) and symptom limitation (dyspnea intensity during constant work rate exercise test) compared to placebo during the randomization and treatment phase in patients with early stage COPD (GOLD stage I / II).

An additional objective of the study is to characterize the mechanisms of exercise limitation and describe daily physical activity in patients with early stage COPD compared to Age / Gender Matched Controls.

2.2 PRIMARY ENDPOINT

The primary efficacy variable will be Inspiratory Capacity (IC) recorded in ml.

The primary endpoint is defined as the difference from baseline to Week 6 post dose in Inspiratory Capacity (IC) during constant work rate treadmill exercise test measured at isotime.

For each individual patient, isotime is defined as the minimum exercise time (ET) among all the constant work rate exercise tests during the treatment periods (Visit 3-6): Isovalue is defined as the value of a specific parameter at isotime: This isovalue will be the observed end-of-exercise value for the specific exercise test with the minimum ET: For all other exercise tests, which were by definition of longer duration, the isovalue will be determined by interpolation between the values at the two time points immediately above and below isotime.

2.3 KEY SECONDARY ENDPOINTS

Difference from baseline to Week 6 post dose:

- Dyspnea intensity during physical exercise: Modified Borg Scale of breathing discomfort (at isotime and isoventilation)
- Constant Work Rate Exercise Duration: calculated as the length of time of the exercise period

2.3.1 Other secondary endpoints

Difference from baseline to Week 6 post dose:

- Percentage of patients with respiratory limitation (as defined by Breathing Reserve <15 L/min)
- Shift in locus of symptom limitation (breathing limited vs. leg limited)
- Locus of symptom limitation (breathing limited vs. leg limited vs. both)

- Ventilatory and respiratory mechanics, metabolic parameters, breathing pattern and operating lung volumes during constant work rate exercise as described in Section 5.1.2.
- Dyspnea intensity at end exercise
- FEV_1 (pre-dose value measured approximately 24 hours after the previous dose of study drug and 90 minutes post-study drug administration)
- Rescue medication use

2.4 OTHER ENDPOINTS

Other endpoints which will explore the differences between early stage COPD patients and Age / Gender Matched Controls are:

- Maximum work rate during incremental exercise test
- Percentage of patients with respiratory limitation (as defined by Breathing Reserve <15 L/min)
- Ventilatory and respiratory mechanics, metabolic parameters, breathing pattern and operating lung volumes during constant work rate exercise as described in Section 5.1.2.
- Measured diffusing capacity for carbon monoxide in early stage COPD compared to Age / Gender Matched Controls
- Dyspnea intensity during physical exercise: Modified Borg Scale (at isotime, isoventilation and at end exercise)
- Leg discomfort during physical exercise: Modified Borg Scale (at isotime, isoventilation and at end exercise)
- Locus of symptom limitation (breathing limited vs. leg limited vs. both)
- BDI dyspnea scale
- Physical activity and quality of life assessment: WPAI-GH, FACIT- short form, VSAQ and accelerometer measurements.
    - Correlation analyses of activity monitor outputs, including measures of physical activity and active energy expenditure over time with: (a) clinical endpoints, (b) respiratory symptoms scores and (c) physical activity questionnaires will be conducted.

2.5 SAFETY ENDPOINTS

Safety and tolerability endpoints will include:

- All adverse events
- Withdrawal due to AEs

3. DESCRIPTION OF DESIGN AND TRIAL POPULATION

3.1 OVERALL TRIAL DESIGN AND PLAN - DESCRIPTION

This is a 22-week, multi-centre, multi-national, randomized, double-blind, two-period cross-over study to evaluate the effects of once-daily administration of tiotropium bromide (Spiriva® HandiHaler®, 18 µg) and placebo on dynamic hyperinflation and physical exercise endurance after 6 weeks of treatment in patients with early stage Chronic Obstructive Pulmonary Disease.

An initial Screening Visit (Visit 1) will be conducted during which patients with early stage COPD and Age / Gender Matched Controls will perform a baseline incremental exercise test. These same patients enter a run-in period during which they will conduct a practice constant work rate exercise test at the beginning of the run-in (Visit 2) and a constant work rate exercise test at the end (Visit 3).

Early stage COPD patients who successfully complete Visit 3 and fulfill the inclusion and exclusion criteria will be randomized into the 2 x six-week double-blind, cross-over treatment portion of the study, in which they will receive either Spiriva® HandiHaler or placebo to be taken once daily for six weeks in a randomized sequence.

Each treatment period will be separated by a four-week wash-out period. Patients will be evaluated for an additional 30 days following completion of the last six-week period of randomized treatment, or in case of discontinuation after the final dose of study medication.

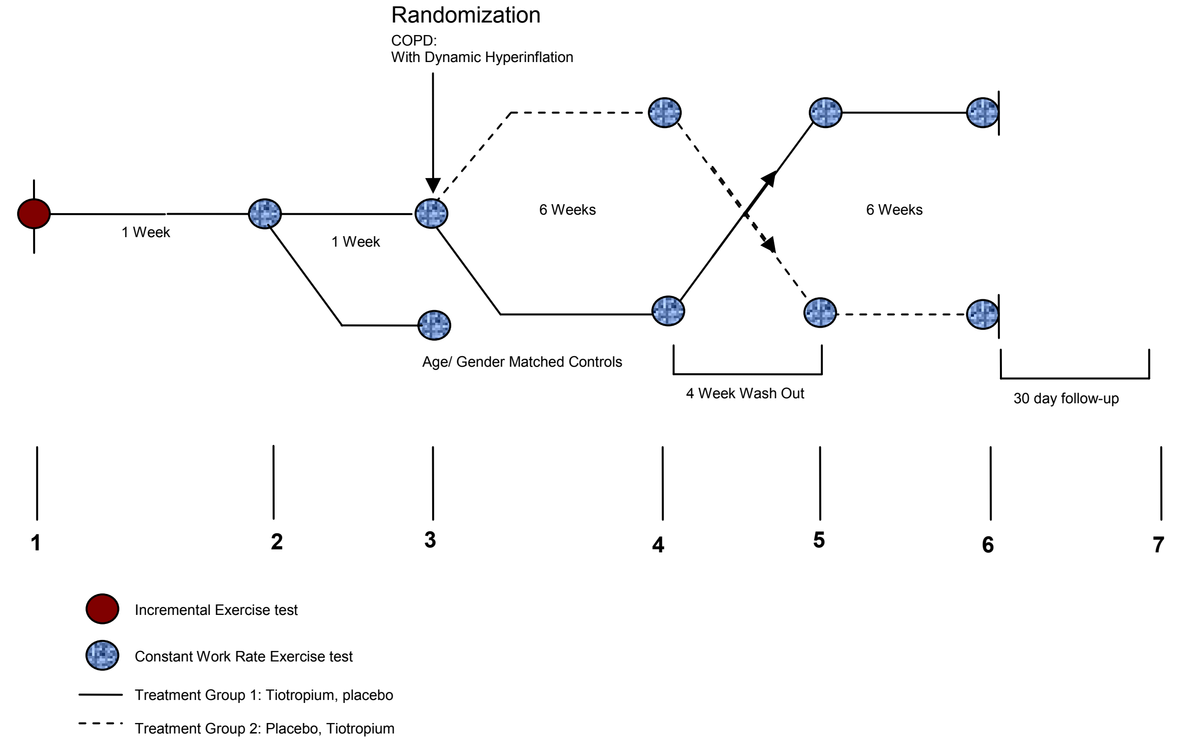

Figure 3.1: 1 Schematic of trial

The investigators selected for this trial are pulmonologists or exercise physiologists with experience in conducting exercise studies and access to the desired patient population.

At Visits 1, and 3 to 6, pulmonary function testing will be conducted in the clinic. At Visit 2, no pulmonary function testing will be conducted. Exercise tests will be conducted three times during the pre-treatment characterization phase (Visits 1, 2 and 3) and also at Visits 4, 5 and 6.

All study-related documentation will be stored in the BI's clinical trial master file (CTMF). Trial relevant documentation for the study sites will be filed in the investigator site file (ISF) at the investigational sites.

3.2 DISCUSSION OF TRIAL DESIGN, INCLUDING THE CHOICE OF CONTROL GROUP

Patients with early stage COPD and Age / Gender Matched Controls will be included in this trial. The trial design has been selected to allow for a characterization of patients with early stage COPD vs Age / Gender Matched Controls and for a comparison of the effects on exercise endurance of tiotropium to a placebo-control in early stage COPD. Comparative data from a controlled, double-blind, randomized trial will provide useful information to health care providers and patients in their choice of maintenance therapies for COPD, particularly as

one of the critical goals of medical treatment is to improve the ability of patients to engage in physical activities.

The randomized, double-blind trial design was chosen to avoid systematic differences between groups with respect to variables that could affect outcome (15). The double-blind design will be used to ensure that patients, investigators and BI personnel are unaware of each patient's assigned treatment, thus minimising any potential biases resulting from differences in management, treatment or assessment of patients, or interpretation of results that could arise as a result of patient or investigator knowledge of the assigned treatment (15).

The cross-over design was chosen because with that design patients will serve as their own control; the comparison between treatments is based on a comparison within patients rather than between patients. This means that the inter-patient variability is removed from the comparison between treatment regimens (16).

Eligible patients with COPD will be allowed to continue their permitted respiratory medications other than inhaled anticholinergics (alone or in combination). See Section 4.2.2 for the full list of permitted and restricted medications. A short-acting β2-agonist medication (albuterol HFA) will also be provided to all patients for rescue use and appropriate medications will be allowed for the treatment of acute exacerbations as medically necessary.

3.3 SELECTION OF TRIAL POPULATION

A suitable number of subjects will be screened to ensure that a minimum of 124 patients of either sex, 40 years of age or older, with a diagnosis of COPD are entered (randomized at Visit 3) into the study with at least 100 patients completing Visits 1 to 7. 100 Age / Gender Matched Controls will complete Visits 1 to 3. All patients are expected to be enrolled within twelve months of overall trial initiation (i.e., initiation of the first site). A minimum enrollment of two to four patients per month at each site is required to complete the patient numbers described above. Enrolment is competitive.

Patients are required to perform symptom-limited exercise tests on several occasions during the trial. For this reason, patients with any contraindication to exercise (as stipulated in the European Respiratory Society (ERS) guidelines (17), and recently supported by the American Thoracic Society (ATS) / American College of Chest Physician [ACCP] guidelines [18]) are excluded from participation in the trial.

A log of all patients screened will be maintained in the ISF at the investigational site.

Every effort should be made to keep patients in the study until they complete all study procedures. Patients who discontinue from randomised treatment can not be re-enrolled at a later date. A record will be kept of all patients who fail to complete all study visits and their reasons for discontinuation.

3.3.1 Inclusion criteria for all trial subjects

1. All subjects must sign an informed consent consistent with ICH-GCP guidelines prior to participation in the trial, which includes medication wash-out and restrictions.

2. Male or female, 40 years of age or older.

3. Subjects must be able to perform technically acceptable pulmonary function tests, must be able to complete multiple symptom-limited exercise tests, must be able to wear the accelerometer and must be able to maintain records (Patient Diary) during the respective study periods as required in the protocol.

4. Patients must have a Body Mass Index (BMI) between 18 and 35 kg/m^2.

3.3.1.1 Additional inclusion criteria for early stage COPD patients

5. All patients must have a diagnosis of chronic obstructive pulmonary disease (19) and must meet the following spirometric criteria:

Patients must have relatively stable airway obstruction (as defined by fewer than three COPD exacerbations in the preceding year or **NO** COPD exacerbation within the six weeks prior to planned study entry) with a post-bronchodilator FEV_1 ≥50% of predicted normal (ECSC [20]) and a post-bronchodilator FEV_1/FVC <70% at Visit 1 (See Section 5.1.2 Pulmonary Function Testing for ECSC predicted normal equations).

6. Patients must be current or ex-smokers with a smoking history of more than 10 pack-years:

$$\text{Pack-years} = \frac{\text{Number of cigarettes/day}}{\text{20 cigarettes/pack}} \times \text{years of smoking}$$

7. Patients must be symptomatic (defined by Baseline Dyspnea Index focal score ≤9 and / or daily cough with production of sputum for three months per year during at least two consecutive years).

8. Patients must exhibit at least a 100 ml decrease in IC during exercise as measured at rest (average of three acceptable ICs measured just prior to start of exercise while standing on the treadmill) compared to the last acceptable IC maneuver during 2 or more pre-randomization exercise tests (Incremental Exercise Test or Constant Work Rate Test) conducted at Visits 1 through 3.

9. Patients must be able to inhale medication in a competent manner from the HandiHaler® inhaler (Appendix 10.1) and from a metered dose inhaler (MDI)

3.3.1.2 Additional inclusion criteria for Age / Gender Matched Controls

5. Patients must be a non-smoker.

Patients will be considered a non-smoker if they have less than a one pack-year history **and** have not had a cigarette in the past two years.

3.3.2 Exclusion criteria for all trial subjects

1. Patients with a significant disease **other** than COPD; a significant disease is defined as a disease which, in the opinion of the investigator, may (i) put the patient at risk because of participation in the study, (ii) influence the results of the study, or (iii) cause concern regarding the patient's ability to participate in the study.

   Note: Additional exclusion criteria for Age / Gender Matched Controls: Patients with a significant disease **including** COPD.

2. Patients with a history of asthma.

3. Patients with any of the following restricted baseline conditions:

   - Hospitalized for heart failure within the past year
   - Known active tuberculosis
   - A malignancy for which patient has undergone resection, radiation therapy or chemotherapy within last five years (patients with treated basal cell carcinoma are allowed)
   - A history of life-threatening pulmonary obstruction
   - Interstitial lung disease
   - Cystic fibrosis
   - Clinically evident bronchiectasis
   - A history of significant alcohol or drug abuse within the past five years
   - Any contraindications for exercise testing as outlined below
   - Patients who have undergone thoracotomy with pulmonary resection (patients with a history of thoracotomy for other reasons should be evaluated as per Exclusion Criterion No. 1)

4. Patients being treated with any restricted pulmonary concomitant medications (see Table 4.2.2: 1).

5. Patients requiring the use of supplemental oxygen therapy.

6. Patients who are currently in the active phase of a pulmonary rehabilitation program or who have completed a pulmonary rehabilitation program in the thirteen weeks prior to the Screening Visit (Visit 1).

7. Patients who have a limitation of exercise performance as a result of factors other than fatigue or exertional dyspnoea, such as arthritis in the leg, angina pectoris,

claudication or other conditions.

8. Patients who have taken an investigational drug within one month or six half-lives (whichever is greater) prior to Screening Visit (Visit 1).
9. Patients with known hypersensitivity to provided rescue medication (β-adrenergic drugs), anticholinergic drugs, lactose or any other component of the HandiHaler® inhalation capsule.
10. Pregnant or nursing women.
11. Women of childbearing potential not using two effective methods of birth control (one barrier and one non-barrier). Female patients will be considered to be of childbearing potential unless surgically sterilised by hysterectomy or bilateral tubal ligation, or post-menopausal for at least two years.
12. Patients who have previously been randomized in this study or are currently participating in another interventional study.
13. Patients who are unable to comply with pulmonary medication restrictions prior to randomization.
14. Patients with known narrow-angle glaucoma.
15. Patients with prostatic hyperplasia or bladder neck obstruction. Patients with symptomatically controlled prostatic hyperplasia on medications may be included and should continue their medications.
16. A respiratory infection or COPD exacerbation in the six weeks prior to the Screening Visit (Visit 1) or during the run-in period.
17. Patients with contraindications to exercise:

    Patients are not allowed to perform an exercise challenge if they are known to have or are suspected of having one of the following contraindications to exercise (17):

    - Recent myocardial infarction (i.e., within one year or less)
    - Unstable angina
    - Unstable, life-threatening or uncontrolled cardiac arrhythmias causing symptoms, haemodynamic compromise, requiring intervention or change in drug therapy during the last year
    - Active endocarditis
    - Acute myocarditis or pericarditis
    - Symptomatic severe aortic stenosis
    - Uncontrolled heart failure
    - Acute non-cardiac disorder that may affect exercise performance or be aggravated by exercise (i.e., infection, renal failure, thyrotoxicosis)
    - History of thrombosis and / or pulmonary embolism
    - Left main coronary stenosis or its equivalent
    - Moderate stenotic valvular heart disease
    - Electrolyte abnormalities

- Severe untreated arterial hypertension (>160 mmHg systolic, >100 mmHg diastolic)
- Significant pulmonary hypertension
- Tachyarrhythmias or bradyarrhythmias
- Hypertrophic cardiomyopathy
- Mental impairment leading to inability to cooperate
- High-degree atrioventricular block

4. TREATMENTS

4.1 TREATMENTS TO BE ADMINISTERED

4.1.1 Identity of investigational products

**Substance:**	Tiotropium bromide (Spiriva®)
**Pharmaceutical formulation:**	Inhalation powder capsule
**Unit strength:**	18 µg of tiotropium
**Device:**	HandiHaler®
**Route of administration:**	Oral inhalation
**Posology:**	1 capsule of 18 µg q.d. (in the morning)

**Substance:**	Placebo (identical matching)
**Pharmaceutical formulation:**	Inhalation powder capsule
**Unit strength:**	-
**Device:**	HandiHaler®
**Posology:**	1 capsule q.d. (in the morning)

Albuterol HFA MDI 100 µg/actuation (see Appendix 10.2 for inhalation aerosol instructions) will be used as the rescue medication for patients with early stage COPD for this trial as well as for use for post-bronchodilator PFTs at Visit 1 for all patients. This will be provided to the sites in bulk, as available from the market in the respective country, and will be labelled with commercial labels. Albuterol will be dispensed at every visit as needed. Albuterol use will not be recorded as Concomitant Medication.

4.1.2 Method of assigning patients to treatment groups

All treatments will be double-blind so that the two treatments will be indistinguishable for the patient as well as for the investigator. COPD patients are assigned to treatment at Visit 3. The treatment for each patient is determined by simple random assignment. After assessment of all inclusion and exclusion criteria, each eligible patient will be assigned a patient kit with the lowest available medication number at the investigational site at the time of randomization. Note that the medication number is different from the patient number which is assigned at trial entry at Visit 1.

Each patient kit will contain two visit boxes: one labeled Visit 3 and one labeled Visit 5. Each visit box will have a unique medication number. Site personnel will enter the medication number for Visit 3 on the corresponding electronic Case Report Form (eCRF) and

also enter the medication number for Visit 5 on the corresponding eCRF. Technical and statistical features of the process of treatment allocation are provided in Section 7.5.

4.1.3 Selection of doses in the trial

In this trial patients are randomized to tiotropium 18 µg inhalation powder from the HandiHaler® or placebo inhalation powder from the HandiHaler®.

4.1.4 Selection and timing of doses for each patient

Study medication administration at clinic visits

Detailed written instructions and training for the use of the HandiHaler® will be given to eligible COPD patients at Visit 3 (see Appendix 10.1). At all subsequent visits (Visits 4-6) detailed instructions on the use of the device will be repeated as needed, but patients should **not** inhale from the training device on those visit days. The investigator or qualified study personnel will observe the inhalation procedure and will reinforce a correct inhalation technique.

At each clinic visit, the subject will self-administer the study medication via oral inhalation of the contents of one capsule using the assigned HandiHaler® while in a seated position under the direct supervision of the investigating physician or study personnel. At each clinic visit, the HandiHaler® that is in current use must be used for administration of the study medication at that visit.

At Visit 3 and 5 the study medication will be self-administered between 7:00 a.m. and 10:00 a.m. after the baseline assessments; Visit 5 study medication must be administered ±30 minutes of time of administration at Visit 3. At all subsequent clinic visits (Visits 4 to 6) all study subjects are **not** to take study medication at home prior to the clinic visit; study medication will be self-administered under the direct supervision of the investigating physician or study personnel. The clock time of the start of inhalation of test medication will be captured on the source documents and in the eCRFs.

When planning the time of the morning dose of study medication at Visit 3, site personnel should discuss with the patient about the preferred regular time of day that the patient will be taking the morning dose of study medication at home.

Study medication administration at home

Each morning between clinic visits, the subject will self-administer the study medication via oral inhalation of the contents of one capsule using the assigned HandiHaler®.

The doses of study medication will be self-administered within ±30 minutes of time of administration at Visit 3 **AND** between 7:00 a.m. and 10:00 a.m. The patient will record whether he / she took the daily dose of test medication in a patient specific diary. If a patient misses a dose, he / she should take the next dose at the next scheduled time and make a note

of the missed dose in his / her diary. Patients will be instructed to withhold their morning dose of study medication on the day of clinic visit (Visit 4-6).

Study medication return

The HandiHaler® dispensed during the treatment period will be used for approximately 42 days each. All used and unused test medication must be kept in the medication boxes and must be brought to clinic Visits 4 and 6 by the patient.

Any HandiHaler® that has been reported as malfunctioning by a patient or investigator will be returned to the Department of Drug Delivery, Boehringer Ingelheim Pharma GmbH & Co. KG (Germany), for investigation. See the ISF and Section 8.2.1 for specific instructions and for details regarding drug accountability requirements. A detail of the procedure for the return of malfunctioning inhalers is provided in the ISF.

4.1.5 Blinding

This trial incorporates a double-blind trial design. To maintain the blinding of treatment, study medications will be supplied such that the active and placebo treatments are indistinguishable. Boehringer Ingelheim will generate the randomization schedule, prepare and code the medication in a blinded fashion and provide all trial supplies.

Patients, investigators, and everyone involved in the analysis or with an interest in this double-blind trial will remain blinded with regard to the randomized treatment assignments up to database lock.

The randomization code will be kept by Clinical Trial Support (within Medical Data Services / Biostatistics and Data Management) up to database lock. The randomization code will only be released as specified in this protocol (Section 7.5).

An emergency code break will be available to the investigator. Unblinding must only be used in emergency situations when the identity of the study drug must be known by the investigator to provide appropriate medical treatment. Rules for breaking the code for an individual are described in Section 8.2.2. The Clinical Monitor Local (CML) must be contacted prior (if possible) to the site unblinding a patient. Patients unblinded to treatment will not be allowed to continue on study medication.

4.1.6 Packaging, labelling, and re-supply

Boehringer Ingelheim will arrange for the packaging and labelling of study medication.

All study medications will be contained in individual **medication boxes** identified with the trial number and a specific medication number. Each medication box will contain an appropriate amount of Spiriva® or placebo capsules, plus some reserve, for dosing until the next scheduled Visit. One HandiHaler® device will be supplied per patient at Visits 3 and 5.

Drug accountability requirements are described in Section 8.2.1.

One re-supply of trial medication is currently planned during the conduct of this study.

Boehringer Ingelheim will supply the following open-label supplies:

- HandiHalers® devices and placebo capsules for training purposes
- Albuterol HFA MDI inhalation aerosol (100 µg per actuation) for assessment of post-bronchodilator FEV_1 at Visit 1 and PRN for rescue use during the screening, treatment, and follow-up period of the trial. Albuterol will be dispensed to the patient at clinic visits as needed and must be collected following all visit procedures at Visit 6.

4.1.7 Storage conditions

Clinical supplies must be stored in a locked, secure cabinet. Further details are provided in the Investigator's Brochure (IB) and on the country-specific labels, a sample of which will be part of the ISF. A temperature log must be maintained at the site (see Section 8.2.1).

Drug supplies will be kept in their original packaging under the recommended storage conditions and may only be dispensed to trial patients according to the protocol. All trial medication (used and unused) will be returned to the trial sponsor (or designated contractor). Details of the return procedure are provided in the ISF. Throughout the trial, drug receipt, usage and return must be documented and verified. Any discrepancies in drug supplies will be noted and explained.

4.2 CONCOMITANT THERAPY

The investigator must record all medication used by the patient for the three months preceding Visit 1 and throughout the trial, on the eCRF. This record will include the name of the medication, the total daily dose, route of administration, dates when medication was started and stopped, and the indication for medication usage.

4.2.1 Rescue medication and additional treatment

Administration of rescue medication can occur at any point during the trial as deemed necessary by the patient or the investigator. Open-label albuterol HFA MDI (100 µg per puff) will be provided as rescue medication by BI; only the albuterol MDI provided by BI is allowed for rescue medication use. If the patient requires rescue medication during the pulmonary function test (PFT) days (Visits 1-6), the PFTs will be discontinued. The exercise testing should not be conducted and the entire visit should be rescheduled if possible. The medication used, route and 24-hour clock time of administration will be recorded on the Rescue Medication eCRF page.

For rescue medication use outside of the clinic visits, the patient must record the number of puffs of rescue medication used during the daytime and the night-time in his / her diary.

4.2.2 Restrictions

Table 4.2.2: 1 Permitted medications and medication restrictions

Drug Class	Sub-class	Prior to study	Study Period		
			**Baseline Period**	**Treatment Period**	**Follow up Period**
Corticosteroids	Inhaled corticosteroids[1]	not permitted	not permitted	not permitted	not permitted
	Oral corticosteroids[1]	not permitted	not permitted	not permitted	not permitted
Beta-adrenergics / Beta blockers	Inhaled short-acting beta-adrenergics[2]	permitted	**rescue**	**rescue**	permitted
	Inhaled long-acting beta-adrenergics[3]	permitted	permitted	permitted	permitted
	Oral beta-adrenergics	not permitted	not permitted	not permitted	not permitted
	Beta blockers	not permitted	not permitted	not permitted	not permitted
Anticholinergics	Short-acting anticholinergics[5] (inhalation aerosol and nasal spray)	permitted	wash-out	**not permitted**	permitted
	Long-acting anticholinergics[6]	wash-out	not permitted	**study medication**	permitted
Miscellaneous	Other investigational drugs[7]	wash-out	not permitted	not permitted	not permitted
	Cromolyn sodium / nedocromil sodium[8]	not permitted	not permitted	not permitted	not permitted
	Antihistamines, antileukotrienes	permitted	permitted	permitted	permitted
	Methylxanthines	not permitted	not permitted	not permitted	not permitted
	Mucolytics	permitted	permitted	permitted	permitted

1. Not allowed within 6 weeks prior to Visit 1
2. Must have at least an 8-hour washout prior to Visit 1 and all other visits during the protocol
3. Must have at least a 36-hour wash-out prior to Visit 1 and all other visits during the protocol
4. Must have at least a 36 hour wash-out prior to Visit 1 and all other Visits during the protocol
5. e.g. ipratropium bromide (Atrovent®): Must have at least an 8-hour washout prior to Visits 1 & 2 and must be discontinued at least 8 hours prior to Visits 3 and 5
6. e.g. tiotropium bromide (Spiriva®): at least a two week washout prior to Visit 1 and at least four week washout prior to Visit 3
7. washout of at least one month or six half lives (whichever is greater)
8. Intranasal allowed only if prescribed for conditions other than asthma

Patients using long-acting anticholinergics such as tiotropium may switch to a short-acting anticholinergic therapy such as ipratropium bromide (Atrovent®) as maintenance bronchodilator therapy during the baseline period; however, the patient must discontinue the

long-acting anticholinergics at least two weeks prior to Visit 1 (Screening Visit) and at least four weeks (± 2 days) prior to Visit 3 (Randomization Visit). The patient must discontinue ipratropium bromide at least 8 hours prior to Visit 3 (randomization visit). Patients who use ipratropium bromide during the baseline period prior to Visit 3 should resume use during the wash-out period between Visits 4 and 5 and then must discontinue ipratropium bromide again at least 8 hours prior to Visit 5.

Patients using fixed combination anticholinergic / short-acting beta adrenergic therapies such as Berodual®, Combivent® and Duovent® should be switched to the mono-product ipratropium bromide (e.g., Atrovent®) during the baseline period (without a change in the anticholinergic dose). The patient must discontinue ipratropium bromide at least eight hours prior to Visit 3 (Randomization Visit). Patients who use ipratropium bromide during the baseline period prior to Visit 3 should resume use during the wash-out period between Visits 4 and 5 and then must discontinue ipratropium bromide again at least 8 hours prior to Visit 5.

Refer to Section 5.1.2 for medication restrictions prior to PFTs.

4.3 TREATMENT COMPLIANCE

Each COPD patient will complete a diary indicating whether the study medication was taken and the number of puffs of albuterol HFA MDI used per day. The investigator or designee will review these records with the patient at each study visit to assess treatment compliance. On test days, compliance will be guaranteed by administration of the test drug under supervision of the investigating physician or designee.

Each patient will be trained on the correct inhalation using a placebo HandiHaler® at Visit 3 and additional training can be done at the discretion of the investigator and study staff at subsequent visits.

Patients will be asked to return all dispensed HandiHalers® and dry powder capsules (used and unused) to the clinic.

Randomized patients will not be discontinued for lack of compliance without prior discussion with the local clinical monitor.

5. OBSERVATIONS

5.1 EFFICACY - CLINICAL PHARMACOLOGY OR PHARMACODYNAMICS

5.1.1 Primary endpoints

The primary endpoint is defined as the post-dose difference in Inspiratory Capacity (IC) during constant work rate exercise test measured at isotime during constant work rate exercise testing at baseline to after six weeks of tiotropium as compared with after six weeks of placebo. An acceptable IC is defined as the volume between a stable end expiratory level over at least four tidal breaths prior to the maneuver AND a clear plateau at end inspiration of a full and deep inhalation.

Exercise testing:

(Specific testing procedures found in Appendix 10.3 and the Exercise Testing Manual of Procedures which can be found in the ISF)

All study participants including those subjects with early stage COPD and age / gender matched controls will perform an Incremental Exercise Test (IET) at Visit 1 followed by CWR tests at all other Visits set at 80% of the maximum work rate as determined at the initial visit (the Exercise Testing Manual of Procedures, which can be found in the ISF). Should the subject reach or exceed 150 Watts at Visit 1 the incremental test will be repeated after at least 90 minutes later using 15 Watts/min (or 20 Watts/min for control subjects) slope.

Adequacy of work rate for the CWR test will be confirmed at Visit 2. If, at Visit 2 the subject is not able to maintain this work rate for more than 4 minutes, then the CWR will be decreased for Visit 3. If at Visit 2 the exercise duration at the defined CWR exceeds 10 min, then the CWR work rate will be increased for Visit 3. This decrease or increase corresponds to the previous or next stage of the incremental protocol (Exercise Testing Manual of Procedures in the ISF).

If at Visit 3, again, the exercise duration is less than 4 minutes or exceeds 10 min (at the defined CWR), one additional adjustment is allowed and the CWR test will be repeated after at least one day no more than 3 days after Visit 3 (Visit 3r): Specifically, if at Visit 3 the subject is not able to maintain this work rate for more than 4 minutes, then the CWR will be decreased for Visit 3r. If at Visit 3 the exercise duration at the defined CWR exceeds 10 min, then the CWR work rate will be increased for Visit 3r. This decrease or increase corresponds to the previous or next stage of the incremental protocol (Exercise Testing Manual of Procedures in the ISF). If the exercise duration is less than 4 minutes or exceeds 10 minutes (at the defined CWR) at Visit 3r, the study participant should be excluded from further participation.

At Visit 3 the CWR test should be performed *after the pre-dose PFT*, but *prior to randomization and study drug administration*. At Visit 5 the CWR test should be performed *after the pre-dose PFT*, but *prior to study drug administration*. At Visits 4 and 6 the CWR test should commence 2 hours and 15 minutes (+/-10) min after inhalation of study drug.

IC maneuvers will be carried out at rest, at two-minute intervals during exercise, and at end of IET and CWR exercise tests.

During the exercise testing (IET and CWR), patients will be encouraged to continue the exercise by a blinded tester (defined as study staff member who is blinded to whether the subject has COPD or is an Age / Gender Matched Control). At peak exercise, patients will be asked their locus of symptom limitation (legs, breathing or both legs and breathing).

During all exercise testing, pulse oximetry must be recorded and documented; however, only the value prior to testing and the lowest value and time of occurrence will be entered into the eCRF. The test should be stopped if arterial oxygen saturation falls below 85% or an adverse event occurs during the test. In such cases, the investigator must carefully evaluate the subject following conclusion of the exercise study regarding further participation in the study. **If a SAE occurs during the test the patient will be excluded from further participation in the study**.

The exercise parameters (including start time, duration, maximal work capacity, and constant work load) from the Incremental Exercise test and from the CWR test will be recorded in the eCRF.

5.1.2 Secondary and other endpoints

Dyspnea intensity during physical exercise:
Prior to the start of exercise, each subject will be told they will be asked to rate the intensity of dyspnea at rest, every two minutes during exercise and at the end of exercise.

Use of the Borg Scale (21) (Appendix 10.4) to rate dyspnea will be explained to the subject. While showing the scale to the subject, the study coordinator or blinded tester will explain to the subject that the subject should relate the wording on the Borg Scale to the level of the sensation that he / she is experiencing, and then place the end of a finger on a number that best describes the intensity of the sensation; subjects are informed that placing a finger between two numbers is allowed. (Borg Scale numbers will be recorded to the nearest 0.5 units).

The study coordinator or blinded tester will anchor the numbers of the scale, explaining that “0 or nothing at all” corresponds to “no discomfort with your breathing” and “10 or maximal” corresponds to the “most severe discomfort with your breathing that you have ever experienced or could imagine experiencing.”

Subjects are to be given no further information about this sensation. If a subject requests further clarification, he / she will be told to use his / her own individual interpretation as to the meaning of the sensory descriptor. This will ensure that the sensory descriptors are presented to each subject in a standard format.

Sensation of leg discomfort:
As with the sensation of dyspnea, prior to starting exercise, each subject will be told that they will be asked to rate the intensity of leg discomfort associated with the act of walking at rest, every two minutes during exercise and at end exercise. The sensation will be described to the subject as "discomfort in your legs."

Use of the Borg Scale (21) to rate intensity of leg discomfort explained to the subject. While showing the scale to the subject, the study coordinator or blinded tester will explain that the subject should relate the wording on the Borg Scale to the level of the sensation that he / she is experiencing, and then place the end of a finger on a number that best describes the intensity of the sensation- explain that placing a finger between two numbers is allowed. (Borg Scale numbers will be recorded to the nearest 0.5 units).

The study coordinator or blinded tester will anchor the numbers of the scale, explaining that "0 or nothing at all" corresponds to "no discomfort in your legs" and "10 or maximal" corresponds to the "most severe discomfort in your legs that you have ever experienced or could imagine experiencing."

Subjects are to be given no further information about this sensation. If a subject requests further clarification, he / she will be told to use his / her own individual interpretation as to the meaning of the sensory descriptor. This will ensure that the sensory descriptors are presented to each subject in a standard format.

Constant Work Rate Exercise Duration:
Endurance time will be measured using the exercise equipment software and will equal the time after the warm-up from the start of walking to the symptom-limited end of exercise. Refer to Exercise Testing Manual of Procedures found in the ISF for detailed description of the exercise testing procedure.

Locus of symptom limitation:
After completion of each exercise test, subjects will be asked to describe the reasons for stopping exercise by completing a standard questionnaire (Appendix 10.5).

Evaluation of perception of improvement on exercise test:
After completion of exercise tests at Visit 4 and Visit 6, subjects will be asked to rate their performance on their exercise test completed that day compared to their previous exercise test (at Visit 3 and Visit 5 respectively). (Appendix 10.10)

Evaluation of ventilatory mechanics and metabolic parameters:

The relationship between the differences in lung function and exercise parameters and the difference in Borg dyspnea rating and endurance time will be examined. The following variables will be used in this analysis:

- Breathing Reserve calculated as MVV-$V_E$max (MVV will be calculated as 35 * FEV_1)
- $V_E$max/MVV (MVV will be calculated as 35 * FEV_1)
- VO_2 max

- VE/VCO_2
- Flow Volume Loop analysis: Flow and integrated volume may be recorded continuously during exercise testing and may be collected and analyzed by the coordinating investigator
- Measured mean expiratory flow rate (V_T/Te)
- Measured mean inspiratory flow rate (V_T/Ti)
- Measured inspiratory capacity at rest
- T_i/T_{TOT}
- Breathing patterns, including breathing frequency and duty cycle (T_i/T_{TOT}) at given workrate at isotime, isoventilation and end exercise
- End expiratory lung volume during exercise, calculated at TLC-IC
- End inspiratory lung volume during exercise, calculated as [(TLC-IC) + V_T]

Pulmonary Function Testing:

Routine spirometry will be performed prior to exercise testing in accordance with recommended techniques (Spirometry Manual of Procedures in the ISF). At Visit 1 only, prior to incremental exercise testing, lung volumes (TLC, FRC, RV, and SVC) will be measured with body plethysmography, the specific airway resistance (SR_{aw}) will also be assessed and, in addition, a single-breath diffusing capacity for carbon monoxide (D_LCO) will also be measured.

Spirometers and their use, including daily calibration, must meet ATS/ERS criteria (22). Spirometry will be conducted as described in the Spirometry Manual of Procedures which can be found in the ISF. At least three acceptable maneuvers need to be performed. The highest FEV_1 and FVC from an acceptable maneuver will be selected (with a maximum of five attempts).

Predicted normal FEV_1 values will be calculated for patients using the following equations (ECSC, [20]):

For height measured in inches:

Males: FEV_1 predicted (L) = 4.30 x (height (inches)/39.37)-0.029 x age (yrs)–2.49

Females: FEV_1 predicted (L) = 3.95 x (height (inches)/39.37)-0.025 x age (yrs)–2.60

For height measured in meters:

Males: FEV_1 predicted (L) = 4.30 x (height (meters))–0.029 x age (years)–2.49

Females: FEV_1 predicted (L) = 3.95 x (height (meters))-0.025 x age (years)–2.60

Ethnic adjustments may be made as appropriate as per ATS/ERS recommendations (20, 23).

If a patient is unable to complete the PFTs during a visit, the Local Clinical Monitor should be notified as soon as possible. The eCRF will be completed indicating the reason for stopping testing, rescue medication given (if any) and time of rescue medication. Patients who are unable to complete the study visit may leave the clinic only upon instruction from the supervising physician.

Medication Restrictions for Pulmonary Function Testing

- Short-acting beta-adrenergic bronchodilators: not to be taken for at least eight hours prior to each study visit
- Short-acting anticholinergic bronchodilators: not to be taken for at least eight hours prior to (Visit 1 and Visit 2) and discontinued at least eight hours prior to Visit 3.
- Long-acting anticholinergic bronchodilators: not to be taken for at least two weeks prior to Visit 1 and four weeks prior to Visit 3 (± 2 days)
- Long-acting beta-adrenergic bronchodilators and inhaled corticosteroids: must not be taken 36 hours prior to the visit (i.e., morning and evening doses not to be taken the day prior to test-day and not on the morning of test-day)
- Study medication: morning doses should not be taken prior to study visits
- A patient visit may be re-scheduled twice due to lack of medication washout compliance

Other Restrictions on Test days (Visits 1 to 6)

- Patients must remain in the building where the pulmonary function testing is performed and must return to the laboratory at least ten minutes prior to the start of each test
- Patients must refrain from strenuous activity for at least 12 hours prior to the study visit and throughout the testing period
- Smoking should be discouraged for the 12 hours prior to the study visit and throughout the study day and will not be permitted in the 60-minute period prior to study visits.
- Patients should avoid cold temperatures, environmental smoke, dust or areas with strong odors (e.g., perfumes)
    - Coffee, tea, chocolate, cola and other caffeine-containing beverages and foods, and ice-cold beverages are not allowed the morning of the study visit and throughout the testing period. Decaffeinated beverages are acceptable.

Reversibility testing (22)

For the qualifying PFT at the Screening Visit (Visit 1):

Following the completion of three acceptable pre-bronchodilator forced expiratory maneuvers, albuterol will be administered to each patient in order to document the degree of reversibility. After a gentle and incomplete expiration, a dose of 100 μg of albuterol is inhaled in one breath to TLC. The breath is then held for 5–10 s before the subject exhales. Four separate doses (total dose 400 μg) are delivered at approximately 30-s intervals (this dose ensures that the response is high on the albuterol dose–response curve).

Three additional, acceptable, post-bronchodilator forced expiratory maneuvers tests are recorded ≥10 minutes and up to 15 minutes later after the last dose of albuterol is inhaled.

The post-bronchodilator measurements must meet the inclusion criteria specified in Section 3.3.1.

Physical activity evaluations:

- At Visit 1 all subjects will complete the VSAQ Questionnaire (Appendix 10.6), then the WPAI-GH questionnaire (Appendix 10.7), followed by the FACIT-short form questionnaire (Appendix 10.8) will be administered and after that the PFTs will be performed including pre- and post-albuterol PFTs.
- Physical activity and energy expenditure over time will be determined by the Body Media Accelerometer and will be measured continuously for 14 days via accelerometer between Visits 1 to 3; except during Visit 2 the activity monitor should be removed.

At Visit 1, the site should dispense the activity monitor and review the training material with the subject so that the subject is comfortable using the device independently, e.g., wearing the activity monitor on the right upper arm, and turning the activity monitor on and off. Information on how to use the device and how to maintain the device is provided in the Body Media Investigator's Manual (which can be found in the site's ISF).

Subject Diary

COPD subjects will receive a patient diary at Visits 1 through 6. This diary will be used to record the number of rescue albuterol inhalations. The diary will be returned at each subsequent visit, and the investigator will review the information with the subjects at each visit and record the data in the eCRF

Additional Questionnaires:

- Questionnaire: Baseline Dyspnoea Index *(*Appendix 10.9*)*

This questionnaire will be administered at Visits 1.

5.2 SAFETY

All adverse events (AEs) must be recorded according to the procedures noted in Section 8.4.1.

At each visit, all adverse events, regardless of causality, will be recorded on the adverse event page for COPD subjects after review of the patient diary and discussion with the patient. Only serious adverse events will be recorded for Age / Gender Matched Control subjects. Adverse events will be recorded on the eCRFs as non-serious or serious adverse events (SAEs). Serious adverse events, whether or not considered related to the investigational drug, will be recorded on the Serious Adverse Event form located in the ISF and faxed to the Local Clinical Monitor (CML) as soon as site personnel are aware of the event. Every attempt should be made to collect discharge summaries for each hospitalization to provide further details. Additionally, all serious adverse events including all events leading to death, regardless of their relationship to drug, that occur within 30 days after the patient terminates study medication must be reported according to Boehringer Ingelheim SAE procedures.

Significant Adverse Events

According to BI Corporate Standard Operating Procedures (SOP) 05_501_03 ("Adverse Event Processing and Reporting"), "a ***significant*** adverse event is an event that is defined to not meet the serious criteria, but is outlined as "significant" in the protocol of a specific trial because of its type".

No events have been classified as "significant" for this trial.

Listed Adverse Events

- Tiotropium: cf. current Investigator's Brochure (ISF).
- Placebo: Inhalation-induced bronchospasm,
  Throat and other local irritation,
  Cough, and
  Hypersensitivity

Physical Examination

A physical examination will be completed on all patients at the Screening Visit (Visit 1) and again at the end of the second treatment phase (Visit 6).

All abnormal findings at baseline will be recorded on the Medical History / Baseline Condition page in the patient's electronic case report form (eCRF). New abnormal findings or worsening of baseline conditions detected at follow-up physical examinations will be recorded as adverse events on the appropriate eCRF page.

Pulse Rate and Blood Pressure

Pulse rate and blood pressure will be measured and recorded prior to each pulmonary function testing on each clinic day (Visits 1-6). Measurements will always be obtained immediately before pulmonary function testing, with the patient seated and rested for a minimum of five minutes. The same person using the same blood pressure instrument on the same arm should perform all recordings.

Pregnancy Testing

An urine human chorionic gonadotrophin (HCG) test will be performed on all females of child-bearing potential at baseline (Visit 1).

Electrocardiograms

A standard 12-lead electrocardiogram (ECG) will be performed on all patients at the Screening Visit (Visit 1).

The purpose of the screening and baseline ECG (Visit 1) is to obtain information about the patient's baseline condition that may have not been elicited in obtaining the medical history. Therefore, any significant findings from this examination are recorded on the Medical

History / Baseline Condition page. Clinical evaluation of ECGs is the responsibility of the investigator. Please refer to your reference manual for details on equipment set-up, transmission and reports.

5.3 OTHER

Medical History

A complete medical history by body system should be performed on all patients at Visit 1. All active and relevant historical disorders **within the past three years (for cancer within the past five years)** will be recorded on the Medical History / Baseline Conditions page at the Screening Visit (Visit 1).

Smoking Status

The smoking status (current smoker or ex-smoker) of each patient will be recorded at all visits (or at the withdrawal visit if the patient does not complete all study visits). Pack-years will also be assessed at Visit 0.

Time of Last Cigarette

On each study visit day (Visits 1-6), the 24-hour clock time of the last cigarette smoked will be recorded if the patient has smoked a cigarette during the 12 hours prior to the start of the study visit.

Concomitant Therapies

All concomitant therapies taken in the three-month period preceding the Screening Visit and throughout the study will be recorded on the Concomitant Therapy eCRF page.

The investigator must record all medication used by the patient for the three months preceding the Screening Visit and throughout the trial, on the eCRF. This record will include the name of the medication, the total daily dose, route of administration, dates when medication was started and stopped, and the indication for medication usage.

Record of Investigational and Rescue Medication

A Patient Daily Record will be kept to document the intake of the investigational drug, and the number of albuterol puffs taken (rescue medication).

5.4 APPROPRIATENESS OF MEASUREMENTS

The use of constant work rate via treadmill as a measure of exercise endurance in patients with COPD is consistent with recommendations in published guidelines (Guidance for Industry COPD: Developing Drugs for Treatment and ATS/ACCP Statement on Cardiopulmonary Exercise Testing). Constant work rate testing via treadmill better imitates routine daily activity (24) and has been demonstrated to be suitable for testing of subjects

with low exercise tolerance (25). In addition, a recent study demonstrated that a linear increase work rate treadmill test appears to be representative of the physiologic limitations of the COPD patient during ambulation and that a linear treadmill protocol may be advantageous when evaluating COPD patients (26).

There is no standard protocol for establishing the CWR on a treadmill in patients with COPD. The protocol developed for the trial is based on a linear incremental treadmill protocol developed and validated by the Los Angeles Biomedical Research Institute at Harbor-UCLA Medical Center. (Exercise Testing Manual of Procedures found in the ISF for detailed description of the exercise testing procedure).

Pulmonary function tests will be conducted at all clinic visits except Visit 2 and the follow-up phone call visit (Visit 7). Pulmonary function tests are a validated and well-established measurement tools for lung function testing (22).

An accelerometer will be used for a two-week period between Visits 1 and 3 to measure physical activity. Body Media accelerometers arm bands will be used in this study. Multiple validation and comparison studies have been conducted including studies in COPD (7, 27-39).

All other scheduled measurements are appropriate to assess drug-induced changes in terms of efficacy and safety. The endpoints are standard and accepted endpoints for evaluation of efficacy, safety and tolerability, and are widely used in this type of studies. The timing of all measurements is presented in the Flow Chart.

5.5 DRUG CONCENTRATION MEASUREMENTS - PHARMACOKINETICS

This section is not applicable to this protocol, since pharmacokinetic analyses will not be performed.

5.6 BIOMARKER - PHARMACODYNAMIC SAMPLING

This section is not applicable to this protocol, since pharmacodynamic analyses will not be performed.

5.7 PHARMACOKINETIC - PHARMACODYNAMIC RELATIONSHIP

This section is not applicable to this protocol, since pharmacokinetic-pharmacodynamic analyses will not be performed.

5.8 DATA QUALITY ASSURANCE

This trial will be conducted in accordance with the International Conference on Harmonization (ICH) Good Clinical Practice (GCP) guidelines, local regulations and the company standard operating procedures (SOPs).

An appropriate number of investigator meetings will take place prior to the initiation of the trial to ensure standardization of procedures and techniques across multiple sites.

Data will be collected using a Remote Data Capture (RDC) system. Training will be provided to all investigators, coordinators and field monitors to ensure consistency and accuracy of the data. The data will be source verified by the field monitors.

6. INVESTIGATIONAL PLAN

6.1 VISIT SCHEDULE

This study consists of two phases:

1) A pre-treatment characterization phase and
2) A randomization / treatment phase.

1. During the pre-treatment characterization phase (Visits 1-3) both eligible patients with COPD and Age / Gender Matched Controls (see Section 3.3.1 Inclusion criteria), a training constant work rate test (Visit 2) and baseline constant work rate test (Visit 3). The pre-treatment characterization phase may be extended up to four weeks for administrative reasons.

2. During the randomization and treatment phase, eligible COPD patients will be randomized into the first of two treatment periods of the study (Visit 3). Each treatment period will last six weeks with a four-week wash-out between each treatments. The follow-up visit (Visit 7) will take place 30 days after the end of last treatment period (Visit 6). A patient visit may be rescheduled twice due to lack of medication washout and/ or other restriction on test day compliance as long as it is within the acceptable time windows of the protocol. Any rescheduling of patients should be discussed with the Local Clinical Monitor.

6.2 TRIAL PROCEDURES AT EACH VISIT

6.2.1 Screening and run-in phase

Informed Consent (Visit 0)

- Informed consent will be obtained prior to patient participation in the trial, which includes any medication washout procedures or restrictions (e.g., for exercise testing). Upon obtaining consent, the patient will be instructed on the medication washout and other restrictions for the screening pulmonary function testing and incremental work rate testing (Visit 1). See Section 5.1.2 for pulmonary function test restrictions and Appendix 10.3 for exercise test restrictions.
- Demographic data (gender, race, date of birth, height, weight, pack-years, and smoking status) will be recorded.
- Relevant medical history will be recorded.

Initial Screening Visit (Visit 1)

Observations and Procedures:

- Medication wash-out compliance will be verified and the 24-hour clock time of the last cigarette smoked during the 12 hours prior to the start of pulmonary function testing will be recorded.
- Physical activity questionnaires (VSAQ, WPAI-GH and the FACIT-short form) will be completed by the subject.
- Baseline Dyspnea Index (BDI) questionnaire will be completed by the subject.
- COPD background characteristics and duration will be recorded (if applicable).
- Physical examination including vital signs (blood pressure and pulse rate) and 12-lead ECG will be conducted. The vital signs (seated) and ECG should be conducted following five minutes rest and prior to the albuterol dosing.
- Urinalysis for pregnancy testing will be conducted (if applicable).
- For the COPD patients all adverse events experienced since signed informed consent will be reviewed and recorded. For the Age / Gender Matched Controls only SAEs will be reviewed and collected.
- Concomitant therapy for the previous three months will be recorded in the eCRF as described in Section 4.2.
- Body plethysmography measurements will be conducted in the morning between 7:00 -10:00 a.m. After the baseline tests, reversibility testing will be performed (see Section 5.1). Spirometry testing will be obtained within 5 minutes before, or up to 5 minutes after the scheduled time point.
- Patients will perform an incremental test (as described in Appendix 10.3) 90 minutes after pulmonary function reversibility tests are complete (the incremental exercise test must not be <45 minutes after or >150 minutes after pulmonary function reversibility tests are complete). The test duration and the maximum work rate of each patient will be recorded in the eCRF.

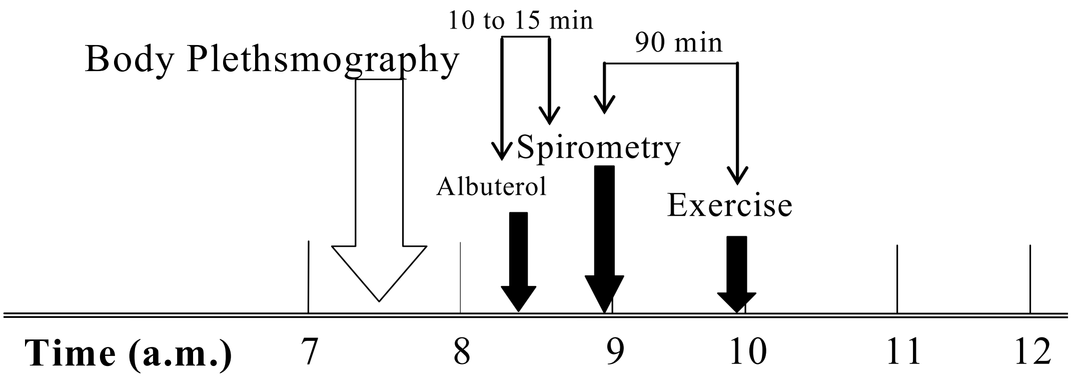

Figure 6.2.1: 1 Visit 1 timeline

- The baseline for rescue medication use will be established during the run-in period from Visit 1 to Visit 3. COPD patients qualified to enter the baseline portion of the study will be issued a patient specific diary, will receive instructions on completing the diary, and restrictions for the baseline period and subsequent visits reviewed. COPD patients qualified to enter the baseline portion of the study will also be issued albuterol HFA MDI and directions on the use of the MDI (for rescue medication use). Patients will be instructed to bring all issued medication (if applicable) and the diary to the clinic on the next scheduled visit. Additional albuterol MDIs will be issued at subsequent visits as needed.
- Both Age / Gender Matched Controls and COPD patients qualified to enter the baseline portion of the study will be given accelerometers and will be instructed how to use them.

Run –in period

Age / Gender Controls and COPD patients who qualify at Visit 0 will enter the run-in period of the trial. Each site will be asked to provide control subjects of the same gender and ±5 years of age of COPD patient randomized to receive study medication. (COPD patients and control subjects do not necessarily need to be from the same site.).

COPD patients who qualify at Visit 1 will measure and record in their diary, the number of puffs of albuterol medication used during the baseline period (Visit 1 to Visit 3).

COPD patients must satisfy **all** inclusion and exclusion criteria prior to randomization at Visit 3 (see Sections 3.2 and 3.3**).** In addition, if during this baseline period there is any indication that the patient is not stable enough to complete the study or that the patient will be non-compliant with the study medication or restrictions, then these patients should not be randomized.

If the baseline period is extended more than an additional 4 weeks, but not more than an additional 8 weeks, the baseline physical examination will be repeated. The repeat baseline examination will include vital signs (blood pressure and pulse rate), 12-lead ECG and pregnancy test. The patient should return for these evaluations two weeks prior to the rescheduled Visit 3. COPD patients should record the number of occasions of albuterol use during the 2 weeks preceding Visit 3 in their diary. All adverse events and concomitant therapies will be recorded. If the baseline period is extended more than an additional eight weeks, the Clinical Monitor should be contacted.

Details of any patient who is screened for the study but is found to be ineligible must be entered in an Enrollment Log and documented in the electronic Case Report Form.

Training Constant Work Rate Visit (Visit 2)

Observations and Procedures:

- Medication washout compliance will be verified and the 24-hour clock time of the last cigarette smoked during the 12 hours prior to the start of the study visit will be recorded.
- Patients smoking status will be recorded.

- Collect patient diary to review rescue medication use since last visit.
- Accelerometer will be removed prior to testing procedures and re-dispensed after completing all visit procedures.
- For the COPD patients all adverse events experienced since the last visit will be reviewed and recorded. For the Age / Gender Matched Controls only SAEs will be reviewed and collected.
- Vital signs (blood pressure and pulse rate).
- Concomitant therapy for the previous three months will be recorded in the eCRF as described in Section 4.2.
- Subjects will conduct the baseline constant work rate test at 80% $W_{cap.}$ (80% of peak work rate in the incremental test).
- COPD patients will be instructed to return completed patient diaries and all rescue medication to the clinic on the next scheduled visit.

6.2.2 Treatment phases

First visit of each treatment period (Visits 3 and 5)

Patients will be contacted prior to each of the visits to remind the patient of medication wash-out times.

Note: Age / Gender Matched Controls will participate through Visit 3 only; Age / Gender Matched controls will not be randomized to double-blind treatment.

Observations / Procedures:

- Medication washout compliance will be verified and the 24-hour clock time of the last cigarette smoked during the 12 hours prior to the start of pulmonary function testing will be recorded at each visit.
- Patients smoking status will be recorded.
- Collect patient diaries to review rescue medication use since last visit.
- Accelerometers will be collected if applicable at Visit 3 only.
- Any adverse events and changes in concomitant therapies will be recorded at each visit.
- **Note:** For **Age / Gender Matched Controls** only SAEs with be recorded**.**
- Vital signs (blood pressure and pulse rate) will be measured. They will always be measured immediately before PFTs and with the patient seated and rested for at least five minutes.
- Pre-study drug spirometry (FEV_1 and FVC) will be performed between 7:00 and 10:00 a.m. Spirometry testing will be obtained within 5 minutes before, or up to 5 minutes after the scheduled time point. Note: All subsequent visit PFTs (Visits 4 to 6) must be conducted within 30 minutes of the spirometry at Visit 3.
- Patients will also conduct a baseline constant work rate endurance test 60 minutes after pulmonary function tests are complete(the constant work rate endurance test must not be <45 minutes after or >150 minutes after pulmonary function tests are complete). The CWR test will be conducted at 80% of the maximum work rate (80% W_{cap}) obtained during the incremental exercise test as described in Appendix 10.3.

- At Visit 3, ONLY COPD patients will be randomized into the cross-over treatment phase of the study after all the inclusion and exclusion criteria have been met.
- Randomized patients will receive instructions on use of the HandiHaler® and the MDI, and restrictions for the treatment period and subsequent visits. The HandiHaler® training device can be used to train patients. Patients will be instructed to bring all study-related treatments to the clinic at each visit.
- Test medications will be dispensed to the randomized patient (see Section 4.1.3) for use the morning of the visit.

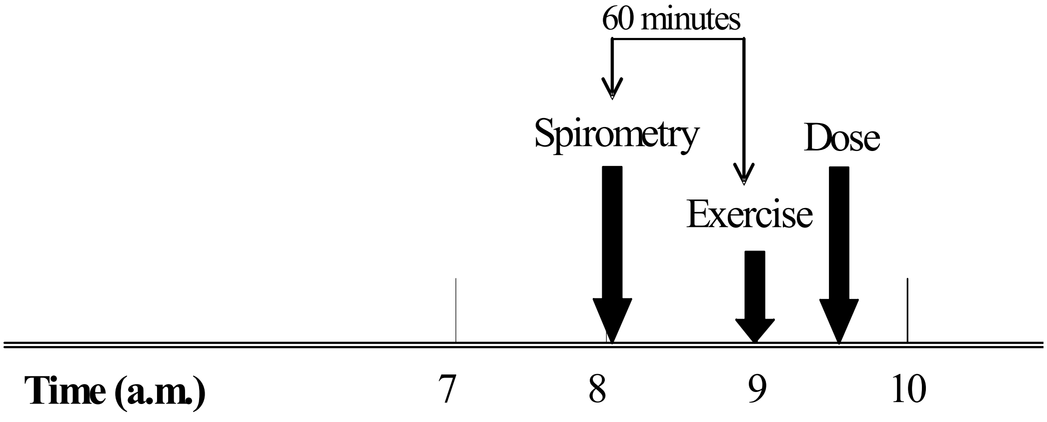

Figure 6.2.2: 1 Visit 3 and 5 timeline

- Randomized patients will be dispensed rescue medication as needed.
- Patients will be issued a paper diary to record trial medication administration and rescue medication use on a daily basis. Patients will be instructed to return to the clinic with the diary at the next scheduled visit so that the diary can be reviewed for treatment compliance.

If the patient is unable to complete the entire test-day visit, the electronic Case Report Form will be completed indicating the reason for stopping testing, rescue medication given and time of rescue medication. Patients who are unable to complete the test-day visit may leave the clinic only upon instruction from the supervising physician. The exact date and clock time of the last drug administration must be recorded in the eCRF (Termination of Trial Medication).

Clinic visits after 6 weeks of each treatment period (Visits 4 and 6)

Observations / Procedures:

Patients will be contacted prior to each of the visits to remind the patient of medication wash-out times, exercise test restrictions and to bring back all study medication at their next visit.

- Medication washout compliance will be verified and the 24-hour clock time of the last cigarette smoked during the 12 hours prior to the start of pulmonary function testing will be recorded at each visit
- Smoking status will be recorded
- Collect patient diary to review rescue medication use and treatment compliance since last visit
- Collect all study medication dispensed at the last visit
- Any adverse events and changes in concomitant therapies will be recorded at each visit
- At Visit 6 only a physical exam will be completed. At both Visits 4 & 6 vital signs (blood pressure and pulse rate) will be measured immediately before spirometry and with the patient seated and rested for at least five minutes
- Spirometry (FEV_1 and FVC) will be performed at the following times relative to the morning dose of study medication:

  Pre-dose: 15 minutes prior to the dose of study medication

  Inhalation of study medication dose

  Post-dose: 1 hour 20 minutes after study medication dose

  - measurements scheduled at 15 minutes prior to the dose will be obtained within ±5 minutes of the specified time-points, and 1 hour 20 minutes post-dose will be obtained within ±15 minutes of the specified time-points

- Patients will conduct a constant work rate endurance test at 2 hours 15 minutes ±15 minutes post dose of trial medication. The CWR test will be conducted at 80% of the maximum work load (80% W_{cap}) obtained during the incremental exercise test as described in Appendix 10.3.

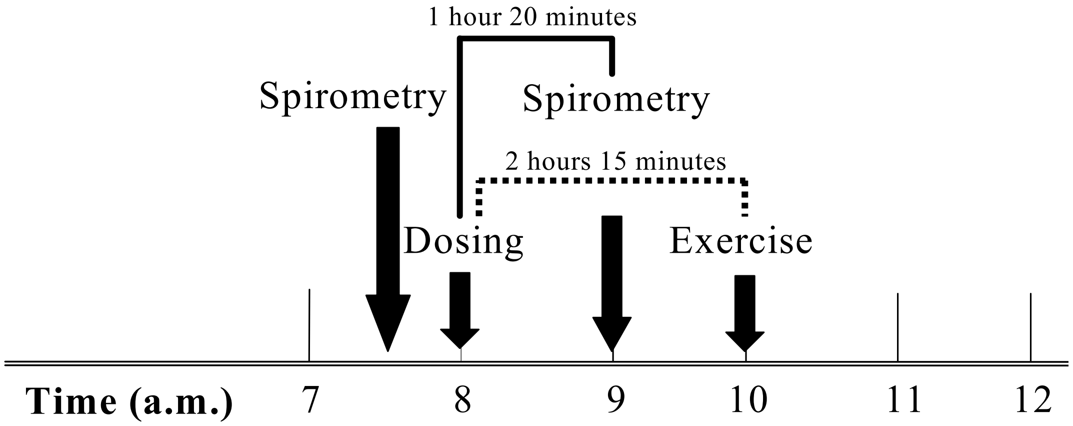

Figure 6.2.2: 2 Visit 4 and 6 timeline

- At Visit 4, but not at Visit 6, patients will be issued a paper diary to record trial medication administration and rescue medication use on a daily basis. Patients will be instructed to return to the clinic with the diary at the next scheduled visit so that the diary can be reviewed for treatment compliance.
- No trial medication will be dispensed at this visit.
- At Visit 4 dispense rescue medication as needed for wash-out periods.

If the patient is unable to complete the entire test-day visit, the electronic Case Report Form will be completed indicating the reason for stopping testing, rescue medication given and time of rescue medication. Patients, who are unable to complete the test-day visit, may leave the clinic only upon instruction from the supervising physician. The exact date and clock time of the last drug administration must be recorded in the eCRF (Termination of Trial Medication).

6.2.3 End of trial and follow-up (Visit 7)

Following the end of the 2 x six-week cross-over period, patients will be followed up for an additional 30 days. They will be phoned at the end of this period (Visit 7) and their smoking status, adverse events and concomitant therapies will be recorded.

The clinical monitor must be consulted on all persistently abnormal tests and SAEs until it is agreed that follow-up is no longer necessary.

6.2.4 End of trial (premature withdrawal)

The investigator should make every effort to perform a follow-up phone call and collect all used and unused study medication from any patient who withdraws prematurely.

The local clinical monitor must be consulted on all SAEs until it is agreed that follow-up is no longer necessary. Additionally, all SAEs that occur within 30 days after a patient terminates study medication must be reported according to Boehringer Ingelheim SAE procedures.

6.3 REMOVAL OF PATIENTS FROM THERAPY OR ASSESSMENT

Patients may be withdrawn from the trial prior to completion if any of the following criteria are observed:

1. Intercurrent illness or an adverse event, which requires discontinuation of treatment per protocol.

2. An acute COPD exacerbation (defined as an increase or new onset of more than one of the following respiratory symptoms [cough, sputum, sputum purulence, wheezing, dyspnea] with a duration of three or more days requiring treatment with antibiotics and / or systemic [oral, intramuscular or intravenous] steroids).

3 Required use of supplemental oxygen therapy.

4. Oxygen desaturation below 85% for at least 30 seconds during exercise testing.

5. Actively participating in a pulmonary rehabilitation program.

6. Request by the patient to withdraw from the study.

7. Administrative reasons (protocol violations, persistent non-compliance).

8. Decision by Boehringer Ingelheim to discontinue one or all patients.

9. During the treatment period, if the investigator judges that the patient requires initiation of chronic maintenance treatment with tiotropium.

No patient should be discontinued from the trial for a protocol violation before discussion with the clinical monitor.

Dropouts and Withdrawals

To be considered complete, a patient must complete all study visits as specified in the protocol without violations of the protocol so significant as to obscure the response to treatment.

Patients who fail to complete all study visits and all of the testing as specified in the protocol will **not** be considered complete and may **not** be enrolled at a later date and will not be replaced. A record will be kept of all patients who fail to complete all study visits and their reasons for discontinuation.

Once a patient is randomized and treated, all observations outlined in the protocol should be performed, unless the patient withdraws consent at any time (without having to justify the decision). All available data from patients who discontinued during the trial, for whatever reason, will be included in the safety analysis. As noted in Sections 7.3 and 7.4, some data on incomplete patients will also be included in the efficacy analyses. Premature terminations will be reported to the sponsor.

7. STATISTICAL METHODS AND DETERMINATION OF SAMPLE SIZE

7.1 STATISTICAL DESIGN / MODEL

This is a 22-week, multi-centre, multi-national, randomized, double-blind, two-way cross-over study to evaluate the effects of the once-daily administration of tiotropium (Spiriva® HandiHaler®, 18 µg) and placebo on dynamic hyperinflation and physical exercise tolerance after six weeks of treatment in patients with early stage COPD. A mixed effect model for the IC change from baseline after six weeks of treatment, with terms of sequence, treatment, period as fixed effects, baseline as a covariate and subject within sequence as a random effect will be used for primary analysis. Detailed specifications will be provided in Section 7.3.

7.2 NULL AND ALTERNATIVE HYPOTHESES

The treatment difference with tiotropium to placebo will be tested for isotime IC change from baseline at the level of α=0.05 (two-sided). The primary hypothesis can be written as follows:

H_0: Mean change from baseline of isotime IC after six weeks of treatment with tiotropium = Mean change from baseline of isotime IC after six weeks of treatment with placebo

H_1: Mean change from baseline of isotime IC after six weeks of treatment with tiotropium ≠ Mean change from baseline of isotime IC after six weeks of treatment with placebo

Different baseline values will be used for the two treatment periods of the cross-over study.

Visit 3 will be the baseline for the first period, while Visit 5 (after wash-out) will be the baseline for the second period.

7.3 PLANNED ANALYSES

The intent-to-treat (ITT) principle will be used to include as many patient periods as possible in the analysis. All randomized patients with at least one baseline data (at Visit 3 or Visit 5) and two non-missing post-dosing data (at Visit 4 and 6) for the primary endpoint will be included in the full analysis set (FAS). The primary analysis will be carried out on the FAS.

The subset of patients who complied with the protocol in essential criteria will be included in the per-protocol set (PPS). Patients included in the FAS who have important protocol violations will be excluded from PPS. A protocol violation will be considered important if it can be expected to have a distorting influence on the assessment of the primary endpoint. The final decision as to which patients are included in the FAS and PPS will be made prior to unblinding. Further details will be given in the trial statistical analysis plan (TSAP).

All patients treated with at least one dose of study drug (the treated set) will be included in the safety analyses.

A Trial Statistical Analysis Plan (TSAP) will be developed in which the details of the statistical analyses are specified.

7.3.1 Primary analyses

The primary analysis is a mixed effect model comparing the change from baseline of isotime IC after six weeks of treatment, with terms of sequence, treatment, period as fixed effects, baseline as a covariate, and subject within sequence as a random effect.

Adjusted mean values as well as treatment contrasts will be presented together with the 95% confidence intervals.

The primary analysis will be performed on the FAS.

A sensitivity analysis for the primary endpoint will be conducted for the PPS. This will only be done if the FAS and per-protocol set differ by more than 10% in size of the FAS population.

7.3.2 Secondary analyses

The mixed effect model as described for the primary endpoint will be performed for all continuous secondary endpoints. The adjusted mean values as well as the treatment contrasts will be presented together with the 95% confidence intervals.

For constant work rate exercise duration, log transformation will be applied before taking the difference from baseline.

For evaluation of perception of improvement on the exercise test, analysis will exploratory only and will be summarized discriptively.

For categorical endpoint (the locus of symptom limitation), frequency tables by visit as well as a shift table of baseline versus six weeks after treatment will be provided.
All other data, including baseline demographics, baseline disease data, concomitant medication use, will be summarised descriptively using appropriate methods.

All secondary analyses will be performed on the FAS.

The other endpoints comparing COPD patients with Age / Gender Matched Controls will be summarized descriptively, with 95% confidence interval for continuous variable. The first 100 randomized patients and the Age / Gender Matched Controls will be included in the analyses.

7.3.3 Safety analyses

All treated patients will be included in the safety analysis. In general, safety analyses will be descriptive in nature and will be based on BI standards. No hypothesis testing is planned prospectively.

The number of patients with adverse events during the treatment periods will be summarized. An adverse event will be assigned to the treatment at onset. Any adverse events, which occur in Period 1 or wash-out, will be assigned to the treatment in Period 1. Any adverse events, which occur in Period 2 or within 30 days after the last study drug intake in Period 2, will be

assigned to the treatment in Period 2. AEs before Period 1 will be assigned to screening. AEs after 30 days from last study drug intake will be assigned to post-treatment.

Frequency, severity, and causal relationship of adverse events will be tabulated by system organ class and preferred term after coding to the current version of MedDRA.

Blood pressure and pulse rate will be presented for each treatment using descriptive statistics.

Physical examination findings (i.e., vital signs) determined at the end of the double-blind treatment period will be compared with those recorded at the beginning of the study.

7.3.4 Interim analyses

No interim analysis is planned during the course of the study.

7.4 HANDLING OF MISSING DATA

Every effort should be made to collect complete data on each test day. Missing value from Visit 4 and 6 will not be imputed. Missing value of baseline (Visit 3 or 5) will be imputed with the value from the other baseline if available. If not available the patient will not be included in the FAS.

Methods to handle any exceptional cases will be considered only before unblinding and will be documented.

With respect to safety evaluation, it is not planned to impute missing data.

7.5 RANDOMIZATION

This is a cross-over trial with two treatment periods. Patients who meet the in- and exclusion criteria will be randomized in blocks to double-blind treatment sequences (AB and BA) with a ratio of 1:1. BI will arrange for the randomization and the packaging and labeling of study medication.

The randomization list will be generated using a validated system, which involves a pseudo-random number generator so that the resulting treatment will be both reproducible and non-predictable. The block size will be documented in the CTR. Access to the codes will be controlled and documented.

Practical aspects of the treatment allocation process and methods to carry out blinding are detailed in Sections 4.1.2 and 4.1.5.

7.6 DETERMINATION OF SAMPLE SIZE

Based on the post-hoc analyses of the GOLD stage II patients in previous parallel Studies 205.131 and 205.223, and recent publication (4), it is deemed reasonable to assume that the effect between once-daily administration of tiotropium with placebo on IC will be 100 ml in GOLD stage I / II patients, and the standard deviation of change from baseline will be 340 ml. By assuming a correlation, r between the changes from baseline of the two treatment periods for the same patient, the variance of the within-patient difference can be calculated as: var1+var2 -2*r*sqrt(var1*var2)=$(340ml)^2+(340ml)^2-$ 2*r*(340 ml*340 ml), where var1 and var2 are the variances of the change from baseline in

the first and second treatment periods, respectively. Various combinations of correlation and power are used to feed in nQueryAdvisors 6.01 MOT0-1 (one-sample t-test) with the following results:

Table 7.6: 1 Calculated power and associated sample size (SD=340 ml, delta=100 ml)

Correlation	SD of within-subject difference between two periods	Effect size	Power	N
0.3	402	0.249	90	172
0.4	372	0.268	90	148
0.5	340	0.294	80	93
0.5	340	0.294	85	106
0.5	340	0.294	90	124
0.6	304	0.329	80	75
0.6	304	0.329	85	85
**0.6**	**304**	**0.329**	**90**	**100**

In a previous cross-over exercise Study 205.334, which comparing eight-weeks treatment with tiotropium plus salmeterol versus the fixed combination of fluticasone and salmeterol inpatients with COPD, only the first baseline value was collected. If the second baseline value is assumed to be the same as the first baseline, it showed a correlation of 0.5 between the two periods in terms of the change from baseline. However, this study had a relatively low correlation between the two post-dose IC values. Based on the information, it is deemed reasonable to assume a correlation of 0.6 between the changes from baseline of the two treatment periods.

Using a correlation of 0.6 and a type one error rate of 0.05 (two-sided), the sample size with 90% power to detect a 100 ml difference in IC change from baseline requires 100 patients in the analysis set. Assuming a drop-out rate of 20%, a total of 124 patients need to be randomized to ensure 100 completed, as well as to accommodate the block size of four in randomization.

8. ADMINISTRATIVE MATTERS

The trial will be carried out in compliance with the protocol, the principles laid down in the Declaration of Helsinki, version as of October 1996 (as long as local laws do not require to follow other versions), in accordance with the ICH Harmonised Tripartite Guideline for Good Clinical Practice (GCP) and relevant BI SOPs. Standard medical care (prophylactic, diagnostic and therapeutic procedures) remains in the responsibility of the treating physician of the patient.

Insurance Cover: The terms and conditions of the insurance cover are made available to the investigator and the patients via documentation in the ISF (Investigator Site File).

8.1 ETHICS

8.1.1 Independent Ethics Committee or Institutional Review Board

The trial will not be initiated before the protocol and informed consent and patient information form have been reviewed and received approval / favourable opinion from the local Institutional Review Board (IRB) / Independent Ethics Committee (IEC) and, for other countries, as required by local laws and regulations approval by / notification to the regulatory authority. Should a CTP amendment be made that needs IRB / IEC approval and authority notification/approval, the changes in the CTP will not be instituted until the amendment and revised informed consent (if appropriate) have been reviewed and received approval / favourable opinion from the local IRB / IEC and, for other countries, as required by local laws and regulations the regulatory authority has been notified / provided approval. A CTP amendment intended to eliminate an apparent immediate hazard to patients may be implemented immediately providing that the regulatory authority and IRB / IEC are notified as soon as possible and an approval is requested. CTP amendments exclusively for logistical or administrative changes may be implemented with notification to the IRB / IEC.

The constitution of the IRB / IEC must meet the requirements of ICH GCP and of the participating countries. A list of the IRB / IEC members who attended the meeting when the CTP / CTP amendment was discussed, including names and qualifications, needs to be provided by the IRB / IEC to the investigator and to the sponsor. The investigator and the sponsor must provide to the regulatory authorities the name and address of the IRB / IEC along with a statement from the IRB / IEC that it is organised according to GCP and the applicable laws and regulations. The IRB / IEC must perform all duties outlined by the requirements of ICH GCP and of the participating countries.

8.1.2 Patient Information and Informed Consent

Prior to patient participation in the trial, written informed consent must be obtained from each patient (or the patient’s legally accepted representative) according to ICH GCP and to the regulatory and legal requirements of the participating country. Each signature must be personally dated by each signatory and the informed consent and any additional patient-information form retained by the investigator as part of the trial records. A signed

copy of the informed consent and any additional patient information must be given to each patient or the patient's legally accepted representative.

The patient must be informed that his / her personal trial-related data will be used by Boehringer Ingelheim in accordance with the local data protection law. The level of disclosure must also be explained to the patient.

The patient must be informed that his / her medical records may be examined by authorised monitors (CML / CRA) or Clinical Quality Assurance auditors appointed by Boehringer Ingelheim, by appropriate IRB / IEC members, and by inspectors from regulatory authorities.

Should a CTP amendment become necessary, the patient consent form and patient information form may need to be revised to reflect the changes to the CTP. It is the responsibility of the investigator and the sponsor to ensure that an amended consent form is reviewed and has received approval / favourable opinion from the IRB / IEC and for other regions, as required by ICH GCP and by local laws and regulations and the regulatory authority has provided approval / has been notified, and that it is signed by all patients subsequently entered in the trial and those currently in the trial, if affected by the amendment.

8.2 RECORDS

8.2.1 Drug accountability

Drug supplies, which will be provided by the sponsor, must be kept in a secure, limited access storage area under the storage conditions defined by the sponsor. Where necessary, a temperature log must be maintained to make certain that the drug supplies are stored at the correct temperature at all times.

The investigator and/or pharmacist and/or investigational drug storage manager must maintain records of the product's delivery to the trial site, the inventory at the site, the use by each patient, and the return to the sponsor or alternative disposition of unused product(s). These records will include dates, quantities, batch/serial numbers, expiry ('use by') dates, and the unique code numbers assigned to the investigational product(s) and trial patients. The investigator / pharmacist / investigational drug storage manager will maintain records that document adequately that the patients were provided the doses specified by the Clinical Trial Protocol (CTP) and reconcile all investigational product(s) received from the sponsor. At the time of return to the sponsor, the investigator / pharmacist / investigational drug storage manager must verify that all unused or partially used drug supplies have been returned by the clinical trial patient and that no remaining supplies are in the investigator's and/ or patient's possession.

8.2.2 Emergency code break

For blinded trials an emergency code break will be available to the investigator / pharmacist / investigational drug storage manager. This code break may only be opened in emergency situations when the identity of the trial drug must be known to the investigator in order to provide appropriate medical treatment or if required to assure safety of trial participants. If the code break for a patient is opened, the sponsor must be informed immediately. The reason

for opening the code break must be documented on the envelope and appropriate electronic Case Report Form (eCRF) page along with the date and the initials of the person who broke the code.

8.2.3 Case Report Forms (CRFs)

All of the clinical data will be captured via electronic data capture (EDC) using the Oracle Clinical Remote Data Capture system, a web-based tool. The investigator site staff will enter and edit the data via a secure network, with secure access features (username, password and secure identification or username and password – an electronic password system). A complete electronic audit trail will be maintained. The investigator will approve the data using an electronic signature (Ref: 21 CFR Part 11), and this approval is used to confirm the accuracy of the data recorded.

Electronic CRFs (eCRFs) will be used for all patients. The investigator’s data will be accessible from the investigator’s site throughout the trial. Relevant medical history prior to enrolment will be documented at the baseline visit. Thereafter during the trial, narrative statements relative to the patient’s progress during the trial will be maintained. The electronic CRFs must be kept current to reflect patient status at each phase during the course of the trial. The patients must not be identified on the electronic CRF by name. Appropriate coded identification (i.e., Patient Number) must be used. The investigator must make a separate confidential record of these details (patient identification code list) to permit identification of all patients enrolled in a clinical trial in case follow-up is required. While a trial is ongoing and until the access to the database has been terminated, there will be no Documentation of Changes (DOCs). All changes will be requested from the investigator through the EDC system. If a change is necessary once the investigator has no further access to the database, a DOC will be sent to the investigator for confirmation of the change. The investigator’s signature is requested to show he / she agrees with the change that was made. The original DOC is kept by the investigator.

Copies of the electronic CRF together with all data changes made will be supplied to the investigator at the end of the trial. The investigator will be responsible for retaining all records pertaining to the trial as specified in the appropriate contract.

8.2.4 Source documents

Source documents provide evidence for the existence of the patient and substantiate the integrity of the data collected. Source documents are filed at the investigator’s site.

Data reported on the CRFs or entered in the eCRFs that are transcribed from source documents must be consistent with the source documents or the discrepancies must be explained. The investigator may need to request previous medical records or transfer records, depending on the trial; also current medical records must be available.

For eCRFs all data must be derived from source documents.

- Patient identification (gender, date of birth)
- Patient participation in the trial (substance, trial number, patient number, date patient was informed)
- Dates of patient's visits, including dispensing of trial medication
- Medical history (including trial indication and concomitant diseases, if applicable)
- Medication history
- Adverse events (onset date [mandatory], and end date [if available])
- Serious adverse events (onset date [mandatory], and end date [if available])
- Concomitant therapy (start date, changes)
- Originals or copies of PFT, Exercise Tests, 12-lead ECG recording (Visit 1) and laboratory results (in validated electronic format, if available).
- Originals or copies of questionnaires (BDI Dyspnea index focal score, Modified Borg Scale, VSAQ, WPAI questionnaire and FACIT short form) and patient diary.
- Conclusion of Patient's Participation in the trial

8.2.5 Direct access to source data - documents

The investigator / institution will permit trial-related monitoring, audits, IRB / IEC review and regulatory inspection, providing direct access to all related source data / documents. CRFs / eCRFs and all source documents, including progress notes and copies of laboratory and medical test results must be available at all times for review by the sponsor's clinical trial monitor, auditor and inspection by health authorities (e.g., FDA). The Clinical Research Associate (CRA) / on-site monitor and auditor may review all CRFs / eCRFs, and written informed consents. The accuracy of the data will be verified by reviewing the documents described in Section 8.2.4.

8.3 QUALITY ASSURANCE AUDIT

A quality assurance audit of this trial may be conducted by the sponsor or sponsor's designees. The quality assurance auditor will have access to all medical records, the investigator's trial-related files and correspondence, and the informed consent documentation of this clinical trial.

8.4 PROCEDURES

8.4.1 Adverse events

An adverse event (AE) is defined as any untoward medical occurrence, including an exacerbation of a pre-existing condition, in a patient in a clinical investigation who received a pharmaceutical product. The event does not necessarily have to have a causal relationship with this treatment.

All adverse events occurring with COPD patients during the course of the clinical trial (i.e., from signing the informed consent onwards through the observational phase) will be collected, documented and reported to the sponsor by the investigator according to the

specific definitions and instructions detailed in the 'Adverse Event Reporting' section of the Investigator Site File.

A SAE is defined as any AE which results in death, is immediately life-threatening, results in persistent or significant disability / incapacity, requires or prolongs patient hospitalisation, is a congenital anomaly / birth defect, or is to be deemed serious for any other reason if it is an important medical event when based upon appropriate medical judgement which may jeopardise the patient and may require medical or surgical intervention to prevent one of the other outcomes listed in the above definitions.

For Age / Gender Matched Control subjects only serious adverse events (SAEs) will be collected

All adverse events, serious and non-serious, will be fully documented on the appropriate eCRFs. For each adverse event, the investigator will provide the onset, end, intensity, treatment required, outcome, seriousness and action taken with the investigational drug. The investigator will determine the relationship of the investigational drug to all AEs as defined in the 'Adverse Event Reporting' Section of the Investigator Site File.

The basis for judging the intensity of the AE as well as the causal relationship between the investigational product and the AE is described below.

Intensity of event

- Mild: Awareness of sign(s) or symptom(s) which is / are easily tolerated
- Moderate: Enough discomfort to cause interference with usual activity
- Severe: Incapacitating or causing inability to work or to perform usual activities

Causal relationship

Medical judgment should be used to determine the relationship, considering all relevant factors, including pattern of reaction, temporal relationship, de-challenge or re-challenge, confounding factors such as concomitant medication, concomitant diseases and relevant history. Assessment of causal relationship should be recorded in the case report forms.

- Yes: There is a reasonable causal relationship between the investigational drug administered and the AE.
- No: There is no reasonable causal relationship between the investigational drug administered and the AE.

If a SAE is reported from a still-blinded trial, the causal relationship must be provided by the investigator for all potential trial drugs, i.e., the BI trial drug and for all other trial drugs (i.e., any active comparator or placebo according to the trial design).

The investigator has the obligation to report AEs during the specified observational phase. If defined in the CTP, the investigator also has the responsibility to report AEs occurring in a certain period after a patient completes the trial. Any AEs reported to the sponsor during this phase must be documented in the safety database.

If not stipulated differently in the ISF, SAEs are to be reported to the sponsor using the BI Serious Adverse Event Report Form including a documented causal relationship assessment and providing as much detail regarding the SAE as possible. With receipt of follow-up information, all remaining fields on the SAE form are to be completed or updated.

Any serious or significant AE, whether or not considered related to the investigational product, and whether or not the investigational product has been administered, must be reported immediately by telephone / fax to the sponsor. Expedited reporting of serious adverse events, e.g. suspected unexpected serious adverse reactions (SUSARs), will be done according to local regulatory requirements. Further details regarding this reporting procedure are provided in the ISF.

Following every such telephone / fax report, the Clinical Monitor must provide a written report of the serious or significant AE and any sequelae to Corporate Drug Safety according to the appropriate Corporate SOP(s). These narratives, which confirm the information collected by telephone, may give additional information not available at the time of the initial report.

8.4.2 Emergency procedures

Not applicable.

8.5 RULES FOR AMENDING PROTOCOL

All CTP amendments must be documented, dated and signed by all signatories (or their successors) of the original protocol. This also applies to any local amendment that may become necessary. Amendments (excluding those exclusively for administrative or logistical changes) need to be submitted to the IRB / IEC for review/approval or for other countries and to the regulatory authority for approval / notification. (Local Amendments will only be submitted in the countries / centres concerned.)

8.6 DISCONTINUATION OF THE TRIAL BY THE SPONSOR

Boehringer Ingelheim reserves the right to discontinue the trial at any time for the following reasons:

1. Failure to meet expected enrolment goals overall or at a particular trial site,
2. Emergence of any efficacy / safety information that could significantly affect continuation of the trial,
3. Violation of GCP, the CTP, or the contract by a trial site or investigator, disturbing the appropriate conduct of the trial.

The investigator / the trial site will be reimbursed for reasonable expenses incurred in case of trial termination (except in case of the third reason).

8.7 STATEMENT OF CONFIDENTIALITY

Individual patient medical information obtained as a result of this trial is considered confidential and disclosure to third parties is prohibited with the exceptions noted below. Patient confidentiality will be ensured by using patient identification code numbers.

Treatment data may be given to the patient's personal physician or to other appropriate medical personnel responsible for the patient's welfare. Data generated as a result of the trial need to be available for inspection on request by the participating physicians, the sponsor's representatives, by the IRB / IEC and the regulatory authorities.

8.8 PUBLICATION POLICY

Boehringer Ingelheim is as much as possible dedicated to support process of free exchange of relevant scientific information. Any publication of the result of this trial must be consistent with the Boehringer Ingelheim publication policy. The rights of the investigator and of the sponsor with regard to publication of the results of this trial are described in the investigator contract. As a general rule, no trial results should be published prior to finalisation of the Clinical Trial Report (CTR).

9. REFERENCES

9.1 PUBLISHED REFERENCES

1. Adams SG, Anzueto A, Briggs DD Jr, Menjoge SS, Kesten S. Tiotropium in COPD patients not previously receiving maintenance respiratory medications. Respir Med. 2006. 100(9): 1495-503. [P06-08635]

2. Global Initiative for Chronic Obstructive Lung Disease: Global Strategy for the Diagnosis, management and prevention of Chronic Obstructive Pulmonary Disease (Updated 2008). http://www.goldcopd.org/Guidelineitem.asp?l1=2&l2=1&intId=2003. [P09-02891]

3. O'Donnell DE, Aaron S, Bourbeau J, Hernandez P, Marciniuk DD, Balter M, et al. Canadian Thoracic Society recommendations for management of chronic obstructive pulmonary disease-2007 update. Can Respir J. 2007. 14 (Suppl B): 5B-32B. [P08-12032]

4. Kornmann O, Beeh KM, Beier J, Geis UP, Ksoll M, Buhl R. Global Initiative for Obstructive Lung Disease. Newly diagnosed chronic obstructive pulmonary disease. Clinical features and distribution of the novel stages of the Global Initiative for Obstructive Lung Disease. Respiration. 2003. 70(1): 67-75. [R09-1613]

5. Ofir D, Laveneziana P, Webb KA, Lam YM, O'Donnell DE. Mechanisms of dyspnea during cycle exercise in symptomatic patients with GOLD stage I chronic obstructive pulmonary disease. Am J Respir Crit Care Med. 2008. 177(6): 622-9. [R09-1638]

6. O'Donnell DE, Laveneziana P, Ora J, Webb KA, Lam YM, Ofir D, et al. Evaluation of acute bronchodilator reversibility in patients with symptoms of GOLD stage I COPD. Thorax. 2009. 64: 216-223. [P09-04204]

7. Ferrer M, Alonso J, Morera J, Marrades RM, Khalaf A, Aguar MC, et al. Chronic obstructive pulmonary disease stage and health-related quality of life: The Quality of Life of Chronic Obstructive Pulmonary Disease Study Group. Ann. Intern. Med. 1972. 127: 1072-1079. [R08-5483]

8. Watz H, Waschki B, Meyer T, Magnussen H. Physical activity in patients with COPD. Eur Respir J. 2009. 33: 262–272. [R09-1614]

9. Johansson G, Lindberg A, Romberg K, Nordstrom L, Gerken F, Roquet A Bronchodilator efficacy of tiotropium in patients with mild to moderate COPD Prim Care Resp J. 2008. 17 (3): 169-175. [P08-08332]

10. O'Donnell DE, Fluege T, Gerken F, Hamilton A, Webb K, Aguilaniu B, et al. Effects of tiotropium on lung hyperinflation, dyspnoea and exercise tolerance in COPD. Eur Respir J. 2004. 23 (6): 832-40. [P04-05869]

11. Casaburi R, Kukafka D, Cooper CB, Witek TJ, Kesten S. Improvement in exercise tolerance with the combination of tiotropium and pulmonary rehabilitation in patients with COPD. Chest. 2005. 127 (3): 809-817. [P05-02413]

12. Carter R, Nicotra B, Huber G. Differing effects of airway obstruction on physical work capacity and ventilation in men and women with COPD. Chest. 1994. 106: 1730-1739. [R09-1615]

13. Celli B, Decramer M, Kesten S, Liu D, Mehra S, Tashkin DP. Mortality in the 4 Year Trial of Tiotropium (UPLIFT) in Patients with COPD. Am J Respir Crit Care Med. 2009. 180: 948-955. [P09-13779]

14. Decramer M, Celli B, Kesten S, Lystig T, Mehra S, Tashkin DP; UPLIFT investigators. Effect of tiotropium on outcomes in patients with moderate chronic obstructive pulmonary disease (UPLIFT): a prespecified subgroup analysis of a randomised controlled trial. Lancet. 2009. 374(9696): 1171-8. [P09-11278]

15. Draft consensus guideline: choice of control group in clinical trials (released for consultation at step 2 of the ICH process on 07 May 1999 by the ICH steering committee). 1999. [R03-2273]

16. Chow SC, Liu JP, editors. Design and Analysis of Bioavailability and Bioequivalence Studies. New York: Marcel Dekker Inc. 1992. [R94-1529]

17. Roca J, Whipp BJ, Agusti AGN, Anderson SD, Casaburi R, Cotes JE, et al. European Respiratory Society. ERS Task Force on Standardization of Clinical Exercise Testing: clinical exercise testing with reference to lung diseases: indications, standardization and interpretation strategies. Eur Respir J. 1997. 10: 2662-2689. [R98-0973]

18. ATS/ACCP statement on cardiopulmonary exercise testing. Am J Respir Crit Care Med. 2003. 167(2): 211-277. [P03-01262]

19. Global Initiative for Chronic Obstructive Lung Disease: Global Strategy for the Diagnosis, management and prevention of Chronic Obstructive Pulmonary Disease http://www.goldcopd.org/Guidelineitem.asp?l1=2&l2=1&intId=2003. 2008. [P09-02891]

20. Quanjer PH, Tammeling GJ, Cotes JE, Pedersen OF, Peslin R, Yernault JC. Lung volumes and forced ventilatory flows. Report Working Party Standardization of Lung Function Tests, European Community for Steel and Coal. Official Statement of the European Respiratory Society. Eur Respir J. 1993. 6(Suppl 16): 5-40. [R94-1408]

21. Mahler DA. Comparison of clinical dyspnoea ratings and psychological measurements of respiratory sensation in obstructive airway disease. Am Rev Respir Dis. 1987. 135(6): 1229-1233. [R98-2318]

22. Miller MR, Hankinson J, Brusasco V, Burgos F, Casaburi R, Coates A, et al. Standardisation of spirometry. Eur Respir J. 2005. 26(2): 319-338. [P05-12782]

23. Pellegrino R, Viegi G, Brusasco V, Crapo RO, Burgos F, Casaburi R, et al. Interpretative strategies for lung function tests. Eur Respir J. 2005. 26(5): 948-968. [P05-12646]

24. WassermanK, Hansen J, Sue D, Stringer W, Whipp B. Principles of Exercise Testing and Interpretation: Including Pathophysiology and Clinical Applications. Lippincott Williams and Wilkins. Philadelphia. 2005. [R09-1616]

25. Porszasz J, Casaburi R, Somfay A, Woodhouse LJ, Whipp BJ. A treadmill ramp protocol using simultaneous changes in speed and grade. Med Sci Sports Exerc. 2003. 35(9): 1596-1603. [R09-1617]

26. Hsia D, Casaburi R, Pradhan A, Torres E, Porszasz J. Physiologic responses to linear treadmill and cycle ergometer exercise in COPD. ERJ. March 2009. [R09-1618]

27. Langer D, Dobbels F, Pitta F, Burtin C, Verleden G, Dupont L, et al. 479: Physical Activities in Daily Life Remain Abnormal 1 Year after Lung Transplantation. J Heart and Lung Transplantation. 2008. 27(2): S232. [R09-1619]

28. Watz H, Waschki B, Boehme C, Claussen M, Meyer T, Magnussen H. Extrapulmonary effects of chronic obstructive pulmonary disease on physical activity: a cross-sectional study. Am J Respir Crit Care Med. 2008. 177(7): 675-6. [R09-1620]

29. Camillo CA, Pitta F, Possani HV, Barbosa MV, Marques DS, Cavalheri V, et al. Heart rate variability and disease characteristics in patients with COPD. Lung. 2008. 186(6): 393-401. [R09-1621]

30. Pitta F, Takaki MY, de Oliveria N, Sant'Anna T, Fontana AD, Kovelis D, et al. Relationship between pulmonary function and physical activity in daily life in patients with COPD. Respiratory Medicine. 2008. 102: 1203-1207. [R09-1622]

31. Crisafulli E, Bortolotti V, Beneventi C, Bellantone T, Prato F, Eutropio E, et al. Energy Expenditure at Rest and during Walking Test in COPD Patients on Long-Term Oxygen. Validation Study of a Body-monitoring Device. Presented at the European Respiratory Society 18th Annual Congress, Berlin. Abstract P3325. 03-07 October 2008. [R09-1623]

32. Hill K, Woon L, Dolmage T, Goldstein R, Brooks D. The SenseWear Armband Yields Valid and Responsive Measurements of Energy Expenditure in Patients With COPD. Presented at the European Respiratory Society 18th Annual Congress, Berlin. Abst P3324. 03-07 October 2008. [R09-1624]

33. Wilcock E, Cox V, Singh S. Validity and Reproducibility of a Multisensor Accelerometer at Slow Walking Speeds in COPD. Presented at the European Respiratory Society 18th Annual Congress, Berlin. Abst P3326. 03-07 October 2008. [R09-1625]

34. Patel S, Benzo R, Slivka W, Sciurba F. Activity Monitoring and Energy Expenditure in COPD Patients: A Validation Study. COPD: Journal of Chronic Obstructive Pulmonary Disease. 2007. 4(2): 107-12. [R09-1626]

35. Battaglia S, Martino L, Spatafora M, Bellia V. Correlates of Energy Expenditure and Sedentary Life in Free-Living COPD. Presented at the American Thoracic Society Annual Meeting. 2006. [R09-1627]

36. Pitta F, Troosters T, Probst VS, Spruit MA, Decramer M, Gosselink R. Quantifying physical activity in daily life with questionnaires and motion sensors in COPD. Eur Respir J. 2006. 27(5): 1040-55. [R09-1628]

37. Battaglia S, Martino L, Spatafora M, Bellia V. Correlates of Energy Expenditure and Sedentary Life in Free-Living COPD. Presented at the American Thoracic Society Annual Meeting. 2006. [R09-1627]

38. Patel S, Sciurba. Emerging Concepts in Outcome Assessment in COPD Clinical Trials. Seminars in Respir Crit Care Med. 2005. 26: 253-262. [R09-1630]

39. Patel S, Slivka W, Sciurba F. Validation of a Wearable Body Monitoring Device in COPD. Amer J of Respir Crit Care Med. 2004. 169(7): A771. [R09-1631]

40. Verkindre C, Bart F, Aguilaniu B, Fortin F, Guerin JC, le Merre C, et al. The effect of tiotropium on hyperinflation and exercise capacity in chronic obstructive pulmonary disease. Respiration. 2006. 73(4): 420-427. [P06-08536]

9.2 UNPUBLISHED REFERENCES

41. O'Donnell D, Tonge S, Menjoge S, Hart L, Hamilton A. Effect of tiotropium on exercise tolerance and static and dynamic lung volumes in COPD patients (a randomized, double-blind, placebo controlled, parallel group study) (205.223) 22 April 2004. [U04-3016]

10. APPENDICES

10.1 PATIENT'S INSTRUCTIONS FOR USE OF THE SPIRIVA® HANDIHALER®

Spiriva® HandiHaler®
(tiotropium bromide inhalation powder)

Important Information: Do not swallow SPIRIVA capsules. SPIRIVA capsules should only be used with the HandiHaler device. SPIRIVA HandiHaler should only be inhaled by mouth (oral inhalation).

First read the Patient Information that comes with SPIRIVA HandiHaler for important information about using SPIRIVA HandiHaler.

Read these Patient’s Instructions for Use before you start to use SPIRIVA HandiHaler and each time you refill your prescription. There may be new information.

For more information, ask your healthcare provider or pharmacist.

SPIRIVA HandiHaler comes with SPIRIVA capsules and a HandiHaler device. The HandiHaler device is an inhalation device that is for use only with SPIRIVA capsules. Do not use the HandiHaler device to take any other medicine.

**Becoming familiar with SPIRIVA HandiHaler:**	
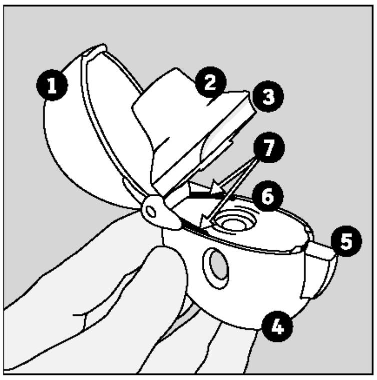<br>Figure A	Remove the HandiHaler device from the pouch and become familiar with its components. (Figure A)<br><br>1. dust cap<br>2. mouthpiece<br>3. mouthpiece ridge<br>4. base<br>5. green piercing button<br>6. center chamber<br>7. air intake vents

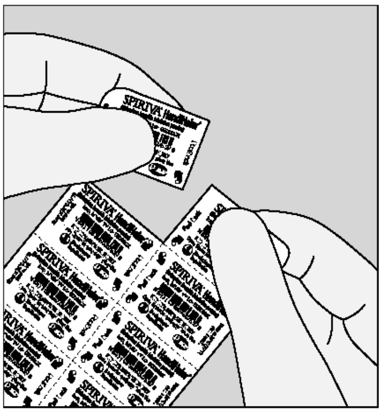<br>Figure B	Each SPIRIVA capsule is packaged in a blister. Each blister can be separated from the blister card by tearing along the perforation. (Figure B)

How do I take my SPIRIVA HandiHaler using the HandiHaler device?

Taking your dose of medicine using the HandiHaler device has four main steps:

1. **Open** the HandiHaler device and the blister
2. **Insert** the SPIRIVA capsule
3. **Press** the green piercing button
4. **Breathe in (inhale)** your medicine

(See below for details)

Opening the HandiHaler device:

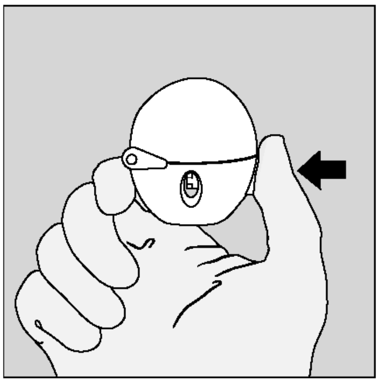<br>Figure 1	**1. Open** the dust cap by pressing the green piercing button. (Figure 1)

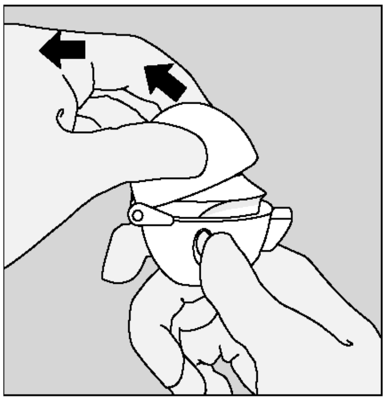 Figure 2	Pull the dust cap upwards to expose the mouthpiece. (Figure 2)

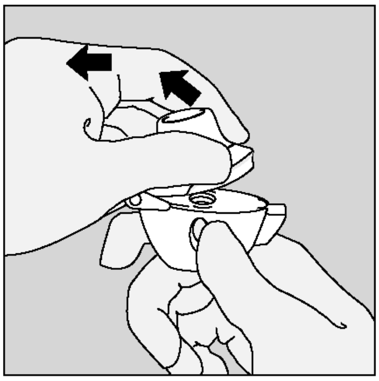 Figure 3	Open the mouthpiece by pulling the mouthpiece ridge upwards away from the base. (Figure 3)
**Removing a SPIRIVA capsule:**	

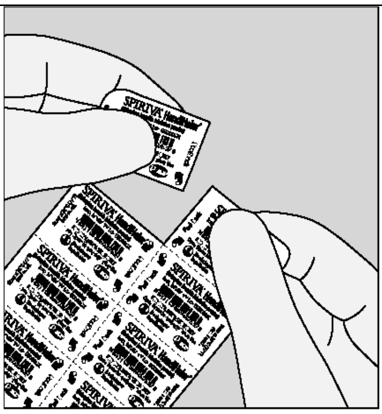 Figure 4	Before removing a SPIRIVA capsule from the blister, separate one of the blisters from the blister card by tearing along the perforation. (Figure 4)<br><br>**Do not swallow Spiriva capsules.**<br><br>**Always store SPIRIVA capsules in the sealed blisters. Remove only one SPIRIVA capsule from the blister right before use. Do not store SPIRIVA capsules in the HandiHaler device. Inhale the contents of the SPIRIVA capsule using the HandiHaler device right away after the blister packaging of an individual SPIRIVA capsule is opened, or else it may not work as well.**

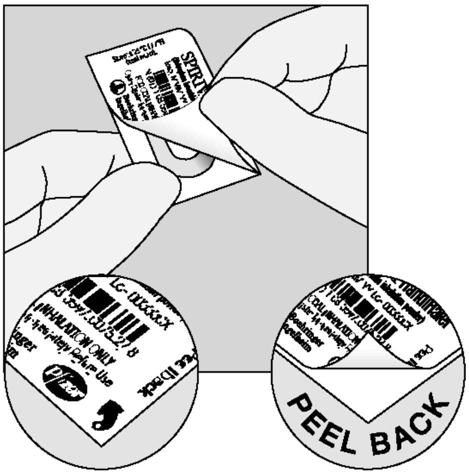<br><br>Figure 5	Right before you are ready to use your SPIRIVA HandiHaler:<br><br>Bend back and forth one of the corners of the blister that has an arrow and then with your finger separate the aluminum foil layers. Carefully peel back the printed foil until you can see the whole SPIRIVA capsule. (Figure 5)<br><br>Turn the blister upside down and tip the SPIRIVA capsule out, tapping the back of the blister, if needed.<br><br>**Do not cut the foil or use sharp instruments to take out the SPIRIVA capsule from the blister.**<br><br>**If more SPIRIVA capsules are opened to air, they should not be used and should be thrown away.**

**Inserting the SPIRIVA capsule into the HandiHaler device:**	
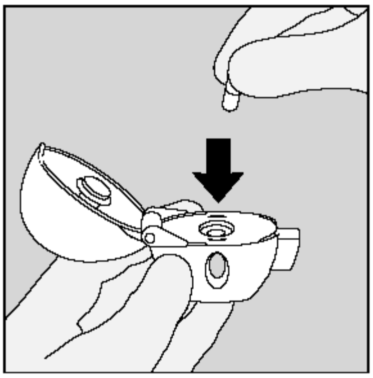<br>Figure 6	**2. Insert** (put) the SPIRIVA capsule in the center chamber of the HandiHaler device. It does not matter which end of the SPIRIVA capsule you put in the chamber. (Figure 6)
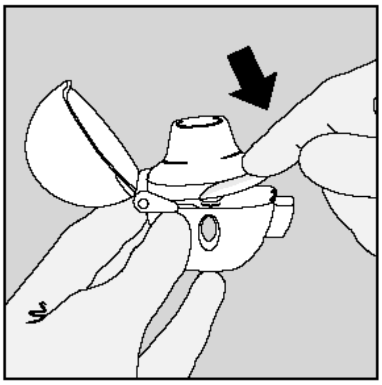<br>Figure 7	Close the mouthpiece **until you hear a click**, but leave the dust cap open. (Figure 7)<br><br>Be sure that you have the mouthpiece sitting firmly against the gray base.

Taking your dose using the HandiHaler device:

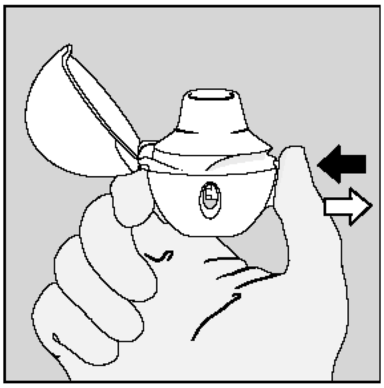

Figure 8

Hold the HandiHaler device with the mouthpiece upright. It is important that you hold the HandiHaler device in an upright position (Figure 8) when pressing the green piercing button.

3. Press the green piercing button until it is flat (flush) against the base, and release. This is how you make holes in the SPIRIVA capsule so that you get the medicine when you breathe in.

Do not press the green button more than one time.

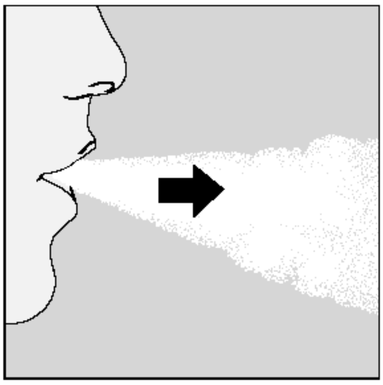

Figure 9

Breathe out completely. (Figure 9)

Important: Do not breathe (exhale) into the mouthpiece of the HandiHaler device at any time.

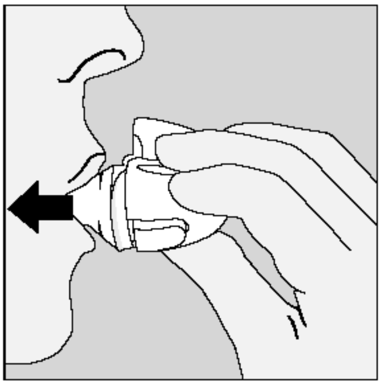

Figure 10

4. Breathe in (inhale)

- Hold the HandiHaler device by the gray base. Do not block the air intake vents.
- Raise the HandiHaler device to your mouth and close your lips tightly around the mouthpiece.
- **Keep your head in an upright position. The HandiHaler device should be in a horizontal position.** (Figure 10)
- Breathe in **slowly and deeply** so that you **hear or feel the SPIRIVA capsule vibrate**.
- Breathe in until your lungs are full.
- Hold your breath as long as is comfortable and at the same time take the HandiHaler device out of your mouth. Breathe normally again.

**To make sure you get the full dose, you must breathe out completely, and inhale again as in step 4 above (Figure 10).** ***Do not press the green piercing button again.***	
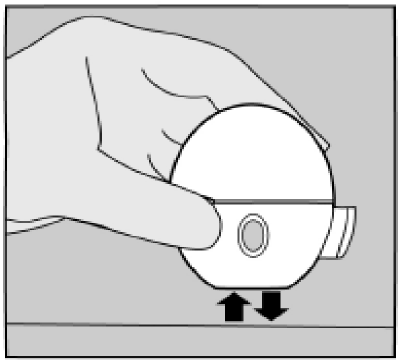 Figure 11	If you do not hear or feel the SPIRIVA capsule vibrate, **do not press the green piercing button again.** Instead, hold the HandiHaler device in an upright position and tap the HandiHaler device gently on a table. (Figure 11)<br><br>Check to see that the mouthpiece is completely closed. Then, breathe in again – slowly and deeply.<br><br>If you still do not hear or feel the SPIRIVA capsule vibrate after repeating the above steps, throw away the SPIRIVA capsule. Open the base by lifting the green piercing button and check the center chamber for pieces of the SPIRIVA capsule (SPIRIVA capsule fragments). SPIRIVA capsule fragments in the center chamber can cause a SPIRIVA capsule not to vibrate. Turn the HandiHaler device upside down and gently tap to remove the SPIRIVA capsule fragments. Call your doctor for instructions.
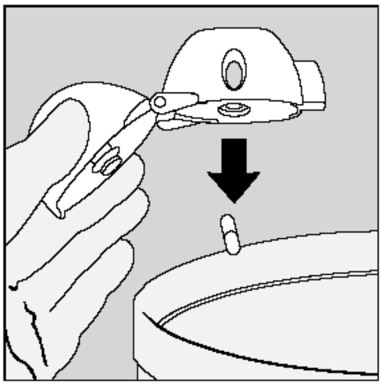 Figure 12	After you finished taking your daily dose of SPIRIVA HandiHaler, open the mouthpiece again. Tip out the used SPIRIVA capsule and throw it away. (Figure 12)

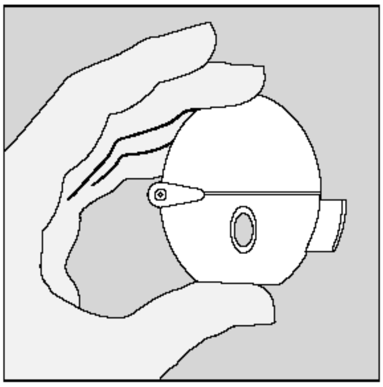

Figure 13

Close the mouthpiece and dust cap for storage of your HandiHaler device. (Figure 13)

Do not store used or unused SPIRIVA capsules in the HandiHaler device.

When and how should I clean my HandiHaler Device?

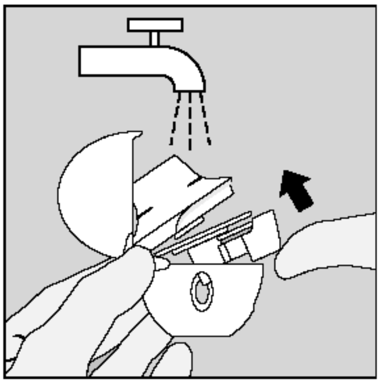

Figure 14

Clean the HandiHaler device one time each month or as needed. (Figure 14)

- Open the dust cap and mouthpiece.
- Open the base by lifting the green piercing button.
- Look at the center chamber for SPIRIVA capsule fragments or powder residue.
- Rinse the HandiHaler device with warm water. Check that any powder buildup or SPIRIVA capsule fragments are removed.
- Do not use cleaning agents or detergents.
- Do not place the HandiHaler device in the dishwasher for cleaning.
- Dry the HandiHaler device well by tipping the excess water out on a paper towel. Air-dry afterwards, leaving the dust cap, mouthpiece, and base open.
- Do not use a hair dryer to dry the HandiHaler device.
- **It takes 24 hours to air dry, so clean the HandiHaler device right after you use it so that it will be ready for your next dose.**
- Do not use the HandiHaler device when it is wet. If needed, you may clean the outside of the mouthpiece with a clean damp cloth.

Distributed by:
Boehringer Ingelheim Pharmaceuticals, Inc.
Ridgefield, CT 06877 USA

Marketed by:
Boehringer Ingelheim Pharmaceuticals, Inc.
Ridgefield, CT 06877 USA
and
Pfizer Inc
New York, NY 10017 USA

Licensed from:
Boehringer Ingelheim International GmbH

SPIRIVA® and HandiHaler® are registered trademarks and are used under license from Boehringer Ingelheim International GmbH.

©Copyright 2009 Boehringer Ingelheim International GmbH
ALL RIGHTS RESERVED

SPIRIVA® (tiotropium bromide inhalation powder) is covered by U.S. Patent Nos. RE38,912, RE39,820, 5,478,578, 6,777,423, 6,908,928, 7,070,800 and 7,309,707 with other patents pending. The HandiHaler® device is covered by U.S. Design Patent No. D355,029 with other patents pending.

IT1600TC1109

10004551/06
65626-06
Revised March 2009

Patient Information

SPIRIVA® (speh REE vah) HandiHaler®
(tiotropium bromide inhalation powder)

Important Information: Do not swallow SPIRIVA capsules. SPIRIVA capsules should only be used with the HandiHaler device. SPIRIVA HandiHaler should only be inhaled by mouth (oral inhalation).

Read the information that comes with your SPIRIVA HandiHaler before you start using it and each time you refill your prescription. There may be new information. This leaflet does not take the place of talking with your doctor about your medical condition or your treatment.

What is SPIRIVA HandiHaler?

SPIRIVA HandiHaler is a prescription medicine that you use one time every day (a maintenance medicine) to control symptoms of chronic obstructive pulmonary disease (COPD). SPIRIVA HandiHaler helps make your lungs work better for 24 hours. SPIRIVA HandiHaler relaxes your airways and helps keep them open. You may start to feel like it is easier to breathe on the first day, but it may take longer for you to feel the full effects of the medicine. SPIRIVA HandiHaler works best and may help make it easier to breathe when you use it every day.

SPIRIVA HandiHaler is **not** a rescue medicine and should not be used for treating sudden breathing problems. Your doctor may give you other medicine to use for sudden breathing problems.

SPIRIVA HandiHaler has not been studied in children.

Who should not take SPIRIVA HandiHaler?

Do not use SPIRIVA HandiHaler if you:

- are allergic to any of the ingredients in SPIRIVA capsules.
- have had an allergic reaction to atropine or any medicines like it, such as ipratropium (Atrovent®).

What should I tell my doctor before using SPIRIVA HandiHaler?

Before taking SPIRIVA HandiHaler, tell your doctor about all your medical conditions, including if you:

- have kidney problems.
- have glaucoma. SPIRIVA HandiHaler may make your glaucoma worse.
- have an enlarged prostate, problems passing urine, or a blockage in your bladder. SPIRIVA HandiHaler may make these problems worse.
- are pregnant or plan to become pregnant. It is not known if SPIRIVA HandiHaler could harm your unborn baby.
- are breast-feeding or plan to breast feed. It is not known if SPIRIVA HandiHaler passes into breast milk. You and your doctor will decide if SPIRIVA HandiHaler is right for you while you breast-feed.

Tell your doctor about all the medicines you take, including prescription and non-prescription medicines and eye drops, vitamins, and herbal supplements. Some of your other medicines or supplements may affect the way SPIRIVA HandiHaler works. SPIRIVA HandiHaler is an anticholinergic medicine. You should not take other anticholinergic medicines while using SPIRIVA HandiHaler, including ipratropium. Ask your doctor or pharmacist if you are not sure if one of your medicines is an anticholinergic.

Know the medicines you take. Keep a list of your medicines with you to show your doctor and pharmacist when you get a new medicine.

How should I take SPIRIVA HandiHaler?

- Use SPIRIVA HandiHaler exactly as prescribed. Use SPIRIVA HandiHaler one time every day.
- Read the “Patient’s Instructions for Use” at the end of this leaflet before you use SPIRIVA HandiHaler. Talk with your doctor if you do not understand the instructions.
- **Do not swallow SPIRIVA capsules.**
- **Only use SPIRIVA capsules with the HandiHaler device.**
- **Do not use the HandiHaler device to take any other medicine**.
- SPIRIVA HandiHaler comes as a powder in a SPIRIVA capsule that fits the HandiHaler device. Each SPIRIVA capsule, containing only a small amount of SPIRIVA powder, is one full dose of medicine.
- Separate one blister from the blister card. Then take out one of the SPIRIVA capsules from the blister package right before you use it.
- After the capsule is pierced, take a complete dose of SPIRIVA HandiHaler by breathing in the powder by mouth two times, using the HandiHaler device (take 2 inhalations from one SPIRIVA capsule). See the “Patient’s Instructions for Use” at the end of this leaflet.
- Throw away any SPIRIVA capsule that is not used right away after it is taken out of the blister package. Do not leave the SPIRIVA capsules open to air; they may not work as well.
- If you miss a dose, take it as soon as you remember. Do not use SPIRIVA HandiHaler more than one time every 24 hours.
- If you use more than your prescribed dose of SPIRIVA HandiHaler, call your doctor or a poison control center.

What should I avoid while using SPIRIVA HandiHaler?

Do not let the powder from the SPIRIVA capsule get into your eyes. Your vision may get blurry and the pupil in your eye may get larger (dilate). If this happens, call your doctor.

What are the possible side effects of SPIRIVA HandiHaler?

SPIRIVA HandiHaler can cause serious side effects. If you get any of the following side effects, stop taking SPIRIVA HandiHaler and get medical help right away.

- **Allergic reaction.** Symptoms may include: itching, rash, swelling of the lips, tongue, or throat (trouble swallowing).
- **Sudden narrowing and blockage of the airways into the lungs (bronchospasm).** Your breathing suddenly gets worse.
- **New or worsened increased pressure in the eyes (glaucoma).** Symptoms of acute narrow-angle glaucoma may include: eye pain, blurred vision, seeing halos (visual halos) or colored images along with red eyes.

Common side effects with SPIRIVA HandiHaler include:

- dry mouth
- constipation
- upper respiratory infection
- increased heart rate
- blurry vision
- glaucoma (new onset or worsening)
- trouble passing urine

These are not all the possible side effects with SPIRIVA HandiHaler. Tell your doctor if you have any side effect that bothers you or that does not go away.

Call your doctor for medical advice about side effects. You may report side effects to FDA at 1-800-FDA-1088.

How do I store SPIRIVA HandiHaler?

- **Do not store SPIRIVA capsules in the HandiHaler device.**
- Store SPIRIVA capsules in the sealed blister package at room temperature [68°–77°F (20°–25°C)].
- Keep SPIRIVA capsules away from heat and cold (do not freeze).
- Store SPIRIVA capsules in a dry place. Throw away any unused SPIRIVA capsules that have been open to air.

Ask your doctor or pharmacist if you have any questions about storing your SPIRIVA capsules.

Keep SPIRIVA HandiHaler, SPIRIVA capsules, and all medicines out of the reach of children.

General information about SPIRIVA HandiHaler

Medicines are sometimes prescribed for purposes other than those listed in Patient Information leaflets. Do not use SPIRIVA HandiHaler for a purpose for which it has not been prescribed. Do not give SPIRIVA HandiHaler to other people even if they have the same symptoms that you have. It may harm them.

For more information about SPIRIVA HandiHaler, talk with your doctor. You can ask your doctor or pharmacist for information about SPIRIVA HandiHaler that is written for health professionals.

For more information about SPIRIVA HandiHaler, you may also call 1-800-542-6257 or (TTY) 1-800-459-9906.

What are the ingredients in SPIRIVA HandiHaler?

Active ingredient: tiotropium
Inactive ingredient: lactose monohydrate

What is COPD (Chronic Obstructive Pulmonary Disease)?

COPD is a serious lung disease that includes chronic bronchitis, emphysema, or both. Most COPD is caused by smoking. When you have COPD, your airways become narrow. So, air moves out of your lungs more slowly. This makes it hard to breathe.

Distributed by:
Boehringer Ingelheim Pharmaceuticals, Inc.
Ridgefield, CT 06877 USA

Marketed by:
Boehringer Ingelheim Pharmaceuticals, Inc.
Ridgefield, CT 06877 USA
and
Pfizer Inc
New York, NY 10017 USA

Licensed from:
Boehringer Ingelheim International GmbH

SPIRIVA® and HandiHaler® are registered trademarks and are used under license from Boehringer Ingelheim International GmbH.

©Copyright 2009 Boehringer Ingelheim International GmbH
ALL RIGHTS RESERVED

SPIRIVA® (tiotropium bromide inhalation powder) is covered by U.S. Patent Nos. RE38,912, RE39,820, 5,478,578, 6,777,423, 6,908,928, 7,070,800, and 7,309,707 with other patents pending. The HandiHaler® device is covered by U.S. Design Patent No. D355,029 with other patents pending.

IT1600TC1109

10004551/06

65626-06

Revised March 2009

A copy of these instructions and patient information will be given to the patient.

10.2 INSTRUCTINS FOR USE OF THE METERED DOSE INHALER (ALBUTEROL)

Patients will be instructed for the use of the MDI as follows:

1. SHAKE THE INHALER WELL immediately before each use. **Then remove the cap from the mouthpiece. Check mouthpiece for foreign objects prior to use.** Make sure the canister is fully inserted into the actuator.

2. As with all aerosol medications, it is recommended to prime the inhaler before using for the first time and in cases where the inhaler has not been used for more than 2 weeks. Prime by releasing four "test sprays" into the air, away from your face.

3. BREATHE OUT FULLY THROUGH THE MOUTH, expelling as much air from your lungs as possible. Place the mouthpiece fully into the mouth, holding the inhaler in its upright position and closing the lips around it.

4. WHILE BREATHING IN DEEPLY AND SLOWLY THROUGH THE MOUTH, FULLY DEPRESS THE TOP OF THE METAL CANISTER with your index finger.

5. HOLD YOUR BREATH AS LONG AS POSSIBLE, up to 10 seconds. Before breathing out, remove the inhaler from your mouth and release your finger from the canister.

6. If your physician has prescribed additional puffs, wait 1 minute, shake the inhaler again, and repeat steps 2 through 4. Replace the cap after use.

7. KEEPING THE PLASTIC MOUTHPIECE CLEAN IS EXTREMELY IMPORTANT TO PREVENT MEDICATION BUILDUP AND BLOCKAGE. THE MOUTHPIECE SHOULD BE WASHED, SHAKEN TO REMOVE EXCESS WATER, AND AIR DRIED THOROUGHLY AT LEAST ONCE A WEEK. INHALER MAY STOP SPRAYING IF NOT PROPERLY CLEANED.

A copy of the patient instructions for each participating country will be filed in the ISF. A copy of these instructions will be given to the patient.

10.3 EXERCISE TESTS

During the initial Screening Visit (Visit 1), an incremental exercise test (IET) will be performed on a treadmill to a symptom-limited maximum (maximum work capacity). In following visits (V2, V3, V4, V5 and V6), a constant work rate exercise endurance test, (symptom-limited endurance exercise test) will be performed in a similar manner at the same constant work rate equal to 80% of the maximum work rate achieved at the IET. During all exercise tests, including the incremental test and all constant work rate tests, the extent of dynamic hyperinflation will be assessed by serial inspiratory capacity measurements (IC). Specific details of the exercise tests are described in the Exercise Testing and Quality Control Manual of Procedures filed in the ISF.

General Considerations for Exercise Testing
Exercise testing equipment will be standardized across study centers to minimize the between-laboratory variability and the technical oversight and quality control can be conducted efficiently. Standardization will eliminate the need for developing technical details for the use of different equipment. In this study all exercise testing will be conducted on treadmill using the Linear Treadmill Protocol that has been developed at the Los Angeles Biomedical Research Institute. In this trial, patients with COPD will perform 10 W/min and the age / gender matched controls will perform 15 W/min ramp test. The constant work rate will be 80% of peak work rate in both populations.

Treadmill equipment

- It is a requirement that the treadmill used in this protocol can be electronically connected to the metabolic and cardiac monitoring equipment so that the subject-specific protocol can be programmed and the actual treadmill parameters be read by the computer of the monitoring equipment.
- The treadmill must have a speed range of up to 16 km/h in 0.2 km/h increments; the slope range is from 0-25% grade; carrying capacity of 180 kg and the walking surface is about 56 cm by 160 cm.
- Calibration of the treadmill must be performed prior to the study (within 6 months). In addition, calibration should be performed if the treadmill is moved or when unusual response profiles raise concerns about the equipment, and/or after any service is done on the treadmill. Documentation of calibration of the equipment must be on file.
- Quality control of the exercise equipment will be performed using a healthy, non-smoking member of the laboratory staff on a regular basis. (See the Exercise Testing and Quality Control Manual of Procedures filed in the ISF for details.) The physiological validation will be done on two constant work rate levels as it is detailed in the Exercise Testing and Quality Control Manual of Procedures filed in the ISF. The results of the physiological validation studies will be used for two purposes: a.) to ensure that all equipments (treadmills and CPET systems) used at all centers are accurate and b.) to establish the precision at each laboratory and across laboratories in the study. The physiological validation studies will be part of source documentation.

ECG and Safety monitoring

- ECG recordings will be monitored during all exercise challenges.
- Blood pressure will be determined before exercise, at the peak of exercise and five minutes after termination of exercise and when, for clinical reasons, it is required. At the same time points heart rate will be determined from ECG.

Personnel qualifications

- Exercise challenges should be conducted by adequately trained personnel with a basic knowledge of exercise physiology.
- Technicians familiar with normal and abnormal responses during exercise and trained in CPR should be present throughout the test.
- Technicians must be certified by Boehringer-Ingelheim staff (or their designee) to perform exercise testing for this clinical trial. A copy of each site staff's certification must be filed in the ISF.
- The supervising physician must be readily available to respond as needed. The degree of subject supervision should be increased if warranted by the clinical status of the subject.

Safety issues

Subjects will not be allowed to perform an exercise challenge if they are known to have or are suspected of having one of the following contra-indications:

- Recent myocardial infarction (i.e., within one year or less)
- Unstable angina
- Unstable, life-threatening or uncontrolled cardiac arrhythmias causing symptoms, haemodynamic compromise, requiring intervention or change in drug therapy during the last year
- Active endocarditis
- Acute myocarditis or pericarditis
- Symptomatic severe aortic stenosis
- Uncontrolled heart failure
- Acute non-cardiac disorder that may affect exercise performance or be aggravated by exercise (i.e., infection, renal failure, thyrotoxicosis)
- History of thrombosis and / or pulmonary embolism
- Left main coronary stenosis or its equivalent
- Moderate stenotic valvular heart disease
- Electrolyte abnormalities
- Severe untreated arterial hypertension (>160 mmHg systolic, >100 mmHg diastolic)
- Significant pulmonary hypertension
- Tachyarrhythmias or bradyarrhythmias
- Hypertrophic cardiomyopathy
- Mental impairment leading to inability to cooperate
- High-degree atrioventricular block

- Cardiac (bradyarrhythmias, ventricular tachycardia, myocardial infarction, heart failure, hypotension, and shock) and non-cardiac (musculoskeletal trauma, severe fatigue,

dizziness, fainting, body aches) complications of exercise challenges have been reported. Consequently during the test, study personnel should be alert to any abnormal event.

- Indications to stop the test must be clearly established and known by the personnel involved in testing:

  Symptoms such as:
    - acute chest pain,
    - sudden pallor,
    - loss of co-ordination,
    - mental confusion,
    - extreme dyspnea.

  Signs such as:
    - clinically significant abnormalities in ECG recordings during exercise. This includes horizontal depression of ST segment > 0.2 mV, T-wave inversion, sustained ventricular tachycardia, polymorphic and / or frequent premature ventricular beats, couplets or triplets particularly of multifocal origin.
    - progressive fall in systolic pressure of 20 mmHg or more less than the previous measurement or below the resting value during exercise.
    - Exercise induced hypertension >250 mmHg systolic and/or >115 mm Hg diastolic.
- If the exercise test has been stopped for one of these reasons, the subject should be monitored in the laboratory until signs/symptoms/ECG abnormalities have completely cleared. Full CPR equipment should be available in the laboratory. In case the exercise test was stopped because of any of the above detailed medical reasons **the subject will be withdrawn from the study**.

Subject preparation

- Subjects should not eat, and should avoid caffeine-containing products, for at least 2 hours before any exercise challenge.
- Subjects should dress appropriately for the exercise challenge.
- On arrival in the laboratory, a detailed explanation of the testing procedure and equipment should be given to the subject, outlining the risks involved and potential complications (see *safety issues* section above).
- Before exercise, ECG electrodes are carefully placed and secured after preparing the skin to ensure good recordings (if necessary, the area of the electrode placement needs to be shaved).
- The subject is informed that it is acceptable to swallow with the mouth piece in place and that he / she must signal if he /she anticipates that within the next minute it will be necessary to stop exercising. However, should any unexpected difficulty arise at any time during the test; it should be signaled by “thumbs down”. This will be used as signal to terminate the test. The subject is advised to point to the site of discomfort if chest or leg pain is experienced.
- Good communication with the subject throughout the whole procedure increases the subjects’ confidence and helps to ensure a good effort by the subject. It is also necessary to restrict any other interaction between the attending personnel.

INCREMENTAL EXERCISE CHALLENGE

In addition to adhering to the general considerations for exercise challenges described above, the following specific procedures should also be adhered to while preparing the test at Visit 1. The incremental ramp protocol for the COPD patients will be uniformly 10 W/min and 15 W/min for the age/ gender matched control subjects. The individualization will be programmed into the RDC system. This will assure that the laboratories will be provided with the parameters of the appropriate protocol the parameters of which will be entered in testing system.

The programmed exercise challenge test protocol provides a total of 15 steps. The target work rate (100 Watts in the COPD group and 150 Watts in the control group) is reached in the 10th step and additional 5 steps for further incrementing the work rate with the same slope. This design allows for each subject to perform the same work rate increment utilizing the same speed-range, regardless of the subject's weight, thus providing a comfortable walking speed throughout the test for everybody.

The speed will be incremented linearly from 0.6 mph to 2.2 mph in the expected range and reaches a maximum of 4 mph in the extended range. The steps of the inclination are calculated based on linearly incremented speed and work rate steps; resulting in a non-linear increase in the treadmill grade.

Entering the appropriate values in the appropriate page on the eCRF, the protocol parameters are automatically calculated and will be displayed on the screen in a table. This table should be saved as source document and will be used to select the treadmill parameters for the constant work rate test (see below).

Should the subject reach or exceed 150 Watts (step 15), the incremental test will be repeated after at least 90 minutes later using 15 Watts/min (or 20 Watts/min for control subjects) slope. The repeat Incremental Exercise Test will recalculate the slope to 15 Watts/minute with a speed range of 0.6–3.0 mph and between 0 and 150 Watts. Similarly, a protocol with 20 Watt/min ramp slope will be generated for the control subjects.

Phases of incremental test:

Start Time (T_{START}): Marks the beginning of the Incremental Exercise Test. T_{START} begins after the 3 minute warm up, when the treadmill registers the speed and grade steps.

Time to Maximal Effort (T_{MAX}): The subject will be encouraged to continue exercising for as long as possible and should be encouraged to complete the time point at which they begin to fatigue. Subjects should continue to exercise (i.e., to exhaustion or maximal exercise) until:

- Limited by symptoms (i.e., is unable to continue exercising because of the discomfort associated with the exercise) or desaturation (oxygen saturation ≤85% for at least 30 seconds)
- Unable to continue safely (in the opinion of the supervising physician).

The Incremental Exercise Time is the duration from T_{START} until T_{MAX} (maximal effort). This excludes the warm-up and recovery periods. (Incremental Exercise Time

(Duration)=T_{MAX} - T_{START} (in minutes and seconds) that will be taken from the report. Subjects who are not able to walk more than 2 minutes due to severe dyspnea or leg pain are excluded from further study. If the subject is able to walk up to or exceed 15 minutes, the test will be repeated using a 15 or 20 Watt/min incremental protocol as described above.

Recovery: After the maximal effort is reached, the subject should continue to walk at the lowest speed (0.6 mph=1.0 km/h) and 0% grade for at least 3 minutes to prevent pooling the blood in the legs and to accelerate lactate removal. If the subject is unable to walk it is possible to offer sitting down on a chair put on the treadmill with feet put up horizontally.

Determining the peak work rate: The peak work rate is equivalent to the work rate of the step in which the subject requested to stop exercising as long as it was possible to complete that step for at least 30 seconds. Otherwise the peak work rate will be equivalent to the work rate of the last completed step.

CONSTANT WORK RATE EXERCISE TEST

The constant work rate will be 80% of the peak work rate rounded to the nearest 5 Watt. The treadmill parameters equivalent to the desired work rate will be calculated in the RDC system. If the desired work rate is between two work rates, both the speed and inclination will be linearly interpolated (average) between the parameters of the neighboring steps.

On Visit 2, the subjects will perform constant walk exercise tests at the determined speed and inclination. During the last minute of the warm-up period, the treadmill inclination will be set to the predetermined grade.

If, the subject is not able to maintain this work rate for more than 4 minutes or is able to maintain this work rate longer than 10 minutes, adjustment of the target work rates will be necessary. The adjustment will be 10% increase or decrease or 5 Watts, whichever is greater. The resulting 10% lower or higher work rate will then be rounded to the nearest 5 Watt. The necessary treadmill parameters will be read off from the same table that was used to design the incremental test.

Data collection during the constant work rate treadmill test
The data collection will be accomplished in part by the cardiopulmonary exercise gas exchange measurement system. The system is able to provide the following variables: Speed, inclination, TMWatt (calculated from the treadmill parameters recorded electronically from the treadmill and the subject's weight), HR, VO2, VCO2, VE, VT, IC, SpO2, Ttot, Ti, Te, Vmax, V_T/Ti, V_T/Te (can be calculated if not provided). Because there is no way to predict the effect of the therapeutic intervention on the exercise endurance, the data collection plan has to adopt a strict timing. Only this will assure that the physiological responses will be comparable at isotime, i.e., at the time point equal to the duration of the shortest test.

In addition to adhering to the general considerations for exercise challenges described above, the following specific procedures should be adhered to during the constant work rate exercise tests:

- The subject will exercise until:
  1. limited by symptoms (i.e., is unwilling to continue exercising because of the discomfort associated with the exercise) <u>OR</u>

2. unable to continue safely (in the discretion of the supervising technician).

At the end of exercise, the time of exercise is recorded (minutes and seconds); the actual endurance will be taken form the cardiopulmonary test report.

- Patients will be asked to rate the intensity of dyspnea using the Borg Category Ratio Scale before the start of the exercise test, every 2 minutes during exercise and at peak exercise.

Changes in end-expiratory lung volume (EELV) will be estimated from inspiratory capacity maneuvers. These will be assessed in 2 minute intervals, in the second half (i.e., 31-59 seconds) of the minute. Techniques for performing and accepting IC measurements are described in detail in the Exercise Testing and Quality Control Manual of Procedures filed in the ISF.

- Symptom intensity ratings (Borg category ratio scale for shortness of breath and leg fatigue) will be assessed in the preceding 30 seconds (i.e., 01–29 sec) prior to the IC measurement. In the 30 sec period after the IC maneuver the blood pressure will be taken.
- ECG monitoring will be done continuously during the exercise test. Heart rate will be recorded from the ECG at 1, 2, 3, 5, 7, 10 minutes and end of exercise whatever comes first.

Recovery:

- ECG monitoring will continue for 5 minutes into the recovery period. Heart rate will be recorded from the ECG at 5 minutes of recovery.
- If possible, the last IC (at peak exercise) should be collected before the subject stops exercising even if it is out of sequence. Subjects will estimate the intensity of discomfort with breathing and leg tiredness at maximal exercise using the modified Borg scale (Appendix 10.4). Large print Borg Scales will be provided.
- Subjects will subsequently be asked to indicate why they stopped exercising (Appendix 10.5)
- At Visits 4 and 6 only, subjects will be asked to rate their performance on the exercise test completed during that visit compared to their performance on the exercise test completed during the previous visit (Visit 3 and 5 respectively). (Appendix 10.10)
- During the recovery period, patients will rate the intensity of dyspnea and perform an IC maneuver at 1, 3 and 5 minutes of recovery. This will allow to study the normalization of hyperinflation.

10.4 BORG SCALE

BORG CATEGORY RATIO SCALE	
10	***Maximal****
9	***Very, very severe*****
8	
7	***Very severe***
6	
5	***Severe***
4	***Somewhat severe***
3	***Moderate***
2	***Slight***
1	***Very slight***
0.5	***Very, very slight******
0	***Nothing at all***

* ***The most severe that you have ever experienced or could imagine experiencing***

** ***Almost maximal***

*** ***Just noticeable***

10.4.1 Instructions for the Borg Scale: intensity of dyspnea and leg discomfort

The directions provided to the subject regarding the rating of the intensity of dsypnea and leg discomfort were standardized. Prior to the start of exercise, each subject was told that he / she would be asked to rate the intensity of two sensations at rest, during exercise, and at peak exercise. The two sensations were described to the subject as:

- Discomfort with your breathing
- Discomfort in your legs

Directions for the use of the Borg Scale (see Section 10.4.1) to rate these sensations was explained to the subject in a standardized manner. While showing the scale to the subject, the study coordinator or blinded tester explained that the subject should relate the wording on the Borg Scale to the level of the sensation that he / she was experiencing, and then place the end of a finger on a number that best described the intensity of the sensation, also explaining that placing a finger between two numbers was allowed. (Borg Scale numbers were recorded to the nearest 0.5 units).

The study coordinator or blinded tester then anchored the endpoints of the scale for both sensations. The study coordinator or blinded tester explained that for the sensation of "discomfort with your breathing", "0 or nothing at all" corresponded to "no discomfort with your breathing" and "10 or maximal" corresponded to the "most severe discomfort with your breathing that you have ever experienced or could imagine experiencing". The study coordinator or blinded tester then explained that for the sensation of "discomfort with your legs", "0 or nothing at all" again corresponded to "no discomfort with your legs" and "10 or maximal" corresponded to the "most severe discomfort with your legs that they you have ever experienced or could imagine experiencing".

In order to ensure that the sensory descriptors were presented to each subject in a standard format, subjects were given no further information about these sensations. If a subject requested further clarification, he / she was told to use his/her own individual interpretation as to the meaning of the sensory descriptors.

10.5 LOCUS OF SYMPTOM LIMITATION QUESTIONAIRE

- Did you stop exercising because of:

  A. Discomfort with your legs?
  B. Discomfort with your breathing?
  C. Both discomfort with your legs and discomfort with your breathing?
  D. None of the above.

- Did you stop exercising because of pain in your chest?

  Yes No

- Did you stop exercising for any other reason?

  Yes No

  If you answered "yes" to question 3, describe the reason:

10.6 VETERANS SPECIFIC ACTIVITY QUESTIONNAIRE (VSAQ)

Before beginning your treadmill test today, we need to estimate what your usual limits are during daily activities. The following is a list of activities which increase in difficulty as you read down the page. Think carefully, then underline the first activity that, if you performed¯it for a period of time, would typically cause fatigue, shortness of breath, chest discomfort, or otherwise cause you to want to stop. If you do not normally perform a particular activity, try to imagine what it would be like if you did.

**1 MET:**	-	**Eating, getting dressed, working at a desk.**
**2 METs:**	-	**Taking a shower, shopping, cooking,<br>Walking down eight steps**
**3 METs:**	-	**Walking slowly on a flat surface for one or two blocks.**
	-	**A moderate amount of work around the house, like vacuuming, sweeping the floors or carrying groceries.**
**4 METs:**	-	**Light yard work, i.e., raking leaves, weeding, sweeping, or pushing a power mower; painting or light carpentry.**
**5 METs:**	-	**Walking briskly, social dancing, washing the car.**
**6 METs:**	-	**Play nine holes of golf carrying your own –clubs, heavy carpentry, mow lawn with push mower.**
**7 METs:**	-	**Carry 60 pounds, perform heavy outdoor work, i.e., digging, spading soil, etc.**
	-	**Walking uphill.**
**8 METs:**	-	**Carry groceries upstairs, move heavy furniture.<br>Jog slowly on flat surface, climb stairs quickly.**
**9 METs:**	-	**Bicycling at a moderate pace, sawing wood, jumping rope slowly.**
**10 METs:**	-	**Brisk swimming, bicycle up a hill, jog six miles per hour.**
**11 METs:**	-	**Carry a heavy load (i.e., a child or firewood) up two flights of stairs.**
	-	**Cross country ski, bicycling briskly, continuously.**
**12 METs:**	-	**Running briskly, continuously, (level ground, eight minutes per mile).**
**13 METs:**	-	**Any competitive activity, including those which involve intermittent sprinting.<br>Running competitively, rowing competitively, bicycle racing.**

10.7 WORK PRODUCTIVITY AND ACTIVITY IMPAIRMENT QUESTIONNIRE: GENERAL HEALTH V2.0 (WPAI-GH)

The following questions ask about the effect of your health problems on your ability to work and perform regular activities. By health problems we mean any physical or emotional problem or symptom. *Please fill in the blanks or circle a number, as indicated.*

1. Are you currently employed (working for pay)? ____ NO ____ YES
   If NO, check "NO" and skip to question 6.

The next questions are about the **past seven days**, not including today.

2. During the past seven days, how many hours did you miss from work because of your health problems? *Include hours you missed on sick days, times you went in late, left early, etc., because of your health problems. Do not include time you missed to participate in this study.*

   _____HOURS

3. During the past seven days, how many hours did you miss from work because of any other reason, such as vacation, holidays, time off to participate in this study?

   _____HOURS

4. During the past seven days, how many hours did you actually work?

   _____HOURS *(If "0", skip to question 6.)*

5. During the past seven days, how much did your health problems affect your productivity while you were working?

   Think about days you were limited in the amount or kind of work you could do, days you accomplished less than you would like, or days you could not do your work as carefully as usual. If health problems affected your work only a little, choose a low number. Choose a high number if health problems affected your work a great deal.

Consider only how much health problems affected productivity while you were working.

Health problems had no effect on my work	0	1	2	3	4	5	6	7	8	9	10	Health problems completely prevented me from working

CIRCLE A NUMBER

6. During the past seven days, how much did your health problems affect your ability to do your regular daily activities, other than work at a job?

By regular activities, we mean the usual activities you do, such as work around the house, shopping, childcare, exercising, studying, etc. Think about times you were limited in the amount or kind of activities you could do and times you accomplished less than you would like. If health problems affected your activities only a little, choose a low number. Choose a high number if health problems affected your activities a great deal.

Consider only how much health problems affected your ability to do your regular daily activities, other than work at a job.

Health problems had no effect on my daily activities	0	1	2	3	4	5	6	7	8	9	10	Health problems completely prevented me from doing my daily activities

CIRCLE A NUMBER

10.8 FACIT-SHORTNESS OF BREATH AND FUNCTIONAL LIMITATION SHORT FORM

Part I. Over the past 7 days, how short of breath did you get with each of these activities?
Please mark one box per line to indicate your response.

		No shortness of breath	Mildly short of breath	Moderately short of breath	Severely short of breath	I did not do this in the past 7 days
1.	Dressing yourself without help.......	☐	☐	☐	☐	☐

Please indicate why you did not do this in the past 7 days:

(Mark one)

☐	☐
I have stopped trying, or knew I could not do this activity because of my shortness of breath.	I did not do this activity for some other reason (including not having a chance to do it, other health issues etc).

		No shortness of breath	Mildly short of breath	Moderately short of breath	Severely short of breath	I did not do this in the past 7 days
2.	Making a bed	☐	☐	☐	☐	☐

Please indicate why you did not do this in the past 7 days:

(Mark one)

☐	☐
I have stopped trying, or knew I could not do this activity because of my shortness of breath.	I did not do this activity for some other reason (including not having a chance to do it, other health issues etc).

		**No shortness of breath**	**Mildly short of breath**	**Moderately short of breath**	**Severely short of breath**	**I did not do this in the past 7 days**
3.	Lifting something weighing 10-20 lbs (about 4.5-9 kg, like a large bag of groceries)	☐	☐	☐	☐	☐

Please indicate why you did not do this in the past 7 days:

(Mark one)

☐	☐
I have stopped trying, or knew I could not do this activity because of my shortness of breath.	I did not do this activity for some other reason (including not having a chance to do it, other health issues etc).

		**No shortness of breath**	**Mildly short of breath**	**Moderately short of breath**	**Severely short of breath**	**I did not do this in the past 7 days**
4.	Scrubbing the floor or counter........	☐	☐	☐	☐	☐

Please indicate why you did not do this in the past 7 days:

(Mark one)

☐	☐
I have stopped trying, or knew I could not do this activity because of my shortness of breath.	I did not do this activity for some other reason (including not having a chance to do it, other health issues etc).

		**No shortness of breath**	**Mildly short of breath**	**Moderately short of breath**	**Severely short of breath**	**I did not do this in the past 7 days**
5.	Sweeping or mopping..................	☐	☐	☐	☐	☐

Please indicate why you did not do this in the past 7 days:

(Mark one)

☐	☐
I have stopped trying, or knew I could not do this activity because of my shortness of breath.	I did not do this activity for some other reason (including not having a chance to do it, other health issues etc).

		**No shortness of breath**	**Mildly short of breath**	**Moderately short of breath**	**Severely short of breath**	**I did not do this in the past 7 days**
6.	Carrying something weighing 10-20 lbs (about 4.5-9 kg, like a large bag of groceries, from one room to another)	☐	☐	☐	☐	☐

Please indicate why you did not do this in the past 7 days:

(Mark one)

☐	☐
I have stopped trying, or knew I could not do this activity because of my shortness of breath.	I did not do this activity for some other reason (including not having a chance to do it, other health issues etc).

		**No shortness of breath**	**Mildly short of breath**	**Moderately short of breath**	**Severely short of breath**	**I did not do this in the past 7 days**

7.	Walking (faster than your usual speed) for 50 steps without stopping	☐	☐	☐	☐	☐

Please indicate why you did not do this in the past 7 days:

(Mark one)

☐	☐
I have stopped trying, or knew I could not do this activity because of my shortness of breath.	I did not do this activity for some other reason (including not having a chance to do it, other health issues etc).

		**No shortness of breath**	**Mildly short of breath**	**Moderately short of breath**	**Severely short of breath**	**I did not do this in the past 7 days**
8.	Moderate-intensity leisure activity (bicycling on level terrain)	☐	☐	☐	☐	☐

Please indicate why you did not do this in the past 7 days:

(Mark one)

☐	☐
I have stopped trying, or knew I could not do this activity because of my shortness of breath.	I did not do this activity for some other reason (including not having a chance to do it, other health issues etc).

		**No shortness of breath**	**Mildly short of breath**	**Moderately short of breath**	**Severely short of breath**	**I did not do this in the past 7 days**
9.	Walking up 30 stairs (3 flights) without stopping	☐	☐	☐	☐	☐

Please indicate why you did not do this in the past 7 days:

(Mark one)

☐	☐
I have stopped trying, or knew I could not do this activity because of my shortness of breath.	I did not do this activity for some other reason (including not having a chance to do it, other health issues etc).

		No shortness of breath	Mildly short of breath	Moderately short of breath	Severely short of breath	I did not do this in the past 7 days
10.	Walking (faster than your usual speed) for at least 1 mile (a little more than 1.5 km) without stopping	☐	☐	☐	☐	☐

Please indicate why you did not do this in the past 7 days:

(Mark one)

☐	☐
I have stopped trying, or knew I could not do this activity because of my shortness of breath.	I did not do this activity for some other reason (including not having a chance to do it, other health issues etc).

Part II: Functional Limitation. Considering your shortness of breath over the past 7 days, rate the amount of difficulty you had when doing the following activities:

		No difficulty	A little difficulty	Some difficulty	Much difficulty
1.	Dressing yourself without help	☐	☐	☐	☐
2.	Making a bed	☐	☐	☐	☐
3.	Lifting something weighing 10-20 lbs (about 4.5-9 kg, like a large bag of groceries)	☐	☐	☐	☐
4.	Scrubbing the floor or counter	☐	☐	☐	☐
5.	Sweeping or mopping	☐	☐	☐	☐
6.	Carrying something weighing 10-20 lbs (about 4.5-9 kg, like a large bag of groceries, from one room to another)	☐	☐	☐	☐
7.	Walking (faster than your usual speed) for 50 steps without stopping	☐	☐	☐	☐
8.	Moderate-intensity leisure activity (bicycling on level terrain)	☐	☐	☐	☐
9.	Walking up 30 stairs (3 flights) without stopping	☐	☐	☐	☐
10.	Walking (faster than your usual speed) for at least 1 mile (a little more than 1.5 km) without stopping	☐	☐	☐	☐

10.9 BASELINE DYSPNEA INDEX QUESTIONAIRE

Baseline Functional Impairment

_____Grade 4	No Impairment	Able to carry out usual activities and occupation without shortness of breath.
_____Grade 3	Slight Impairment	Distinct impairment in at least one activity but no activities completely abandoned. Reduction, in activity at work or in usual activities, that seems slight or not clearly caused by shortness of breath.
_____Grade 2	Moderate Impairment	Subject has changed jobs and/or has abandoned at least one usual activity due to shortness of breath.
_____Grade 1	Severe Impairment	Subject unable to work or has given up most or all usual activities due to shortness of breath.
_____Grade 0	Very Severe Impairment	Unable to work and has given up most or all usual activities due to shortness of breath.
_____W	Amount Uncertain	Subject is impaired due to shortness of breath, but amount cannot be specified. Details are not sufficient to allow impairment to be categorised.
_____X	Unknown	Information unavailable regarding impairment.
_____Y	Impaired for Reasons Other than Shortness of Breath	For example, musculoskeletal problem or chest pain.

Usual activities refer to requirements of daily living, maintenance or upkeep of residence, yard work, gardening, shopping, etc.

Baseline Magnitude of Task

_____Grade 4	Extraordinary	Becomes short of breath only with extraordinary activity such as carrying very heavy loads on the level, lighter loads uphill, or running. No shortness of breath with ordinary tasks.
_____Grade 3	Major	Becomes short of breath only with such major activities as walking up a steep hill, climbing more than three flights of stairs, or carrying a moderate load on the level.
_____Grade 2	Moderate	Becomes short of breath with moderate or average tasks such as walking up a gradual hill, climbing fewer than three flights of stairs, or carrying a light load on the level.
_____Grade 1	Light	Becomes short of breath with light activities such as walking on the level, washing, or standing.
_____Grade 0	No Task	Becomes short of breath at rest, while sitting, or lying down.
_____W	Amount Uncertain	Subject's ability to perform tasks is impaired due to shortness of breath, but amount cannot be specified. Details are not sufficient to allow impairment to be categorised.
_____X	Unknown	Information unavailable regarding limitation of magnitude of task.
_____Y	Impaired for Reasons Other than Shortness of Breath	For example, musculoskeletal problem or chest pain.

Baseline Magnitude of Effort

_____Grade 4	Extraordinary	Becomes short of breath only with the greatest imaginable effort. No shortness of breath with ordinary effort.
_____Grade 3	Major	Becomes short of breath with effort distinctly submaximal, but of major proportion. Tasks performed without pause unless the task requires extraordinary effort that may be performed with pauses.
_____Grade 2	Moderate	Becomes short of breath with moderate effort. Tasks performed with occasional pauses and requiring longer to complete than the average person.
_____Grade 1	Light	Becomes short of breath with little effort. Tasks performed with little effort or more difficult tasks performed with frequent pauses and requiring 50-100% longer to complete than the average person might require.
_____Grade 0	No Effort	Becomes short of breath at rest, while sitting, or lying down.
_____W	Amount Uncertain	Subject's exertional ability is impaired due to shortness of breath, but amount cannot be specified. Details are not sufficient to allow impairment to be categorised.
_____X	Unknown	Information unavailable regarding limitation of effort.
_____Y	Impaired for Reasons Other than Shortness of Breath.	For example, musculoskeletal problems, or chest pain

Appendix 10.10: PGI OF IMPROVEMENT

Patient Global Impression of Improvement

In comparison with your previous test, how would you rate your performance on today's exercise test using the present scale?

1 = Very much improved
2 = Much improved
3 = Minimally improved
4 = No change
5 = Minimally worse
6 = Much worse
7 = Very much worse
